# Identification of intra-group, inter-individual, and gene-specific variances in mRNA expression profiles in the rheumatoid arthritis synovial membrane

**DOI:** 10.1186/ar2485

**Published:** 2008-08-22

**Authors:** René Huber, Christian Hummert, Ulrike Gausmann, Dirk Pohlers, Dirk Koczan, Reinhard Guthke, Raimund W Kinne

**Affiliations:** 1 Experimental Rheumatology Unit, Department of Orthopedics, University Hospital Jena, Waldkrankenhaus 'Rudolf Elle', Klosterlausnitzer Str. 81, 07607 Eisenberg, Germany; 2 Institute for Clinical Chemistry, Hannover Medical School, Carl-Neuberg-Str. 1, 30625 Hannover, Germany; 3 Systems Biology/Bioinformatics Group, Department of Molecular and Applied Microbiology, Leibniz Institute for Natural Product Research and Infection Biology – Hans Knöll Institute, Beutenbergstr. 11a, 07745 Jena, Germany; 4 Genome Analysis, Leibniz Institute for Age Research – Fritz Lipmann Institute, Beutenbergstr. 11, 07745 Jena, Germany; 5 Proteome Center Rostock, University of Rostock, Schillingallee 69, 18055 Rostock, Germany

## Abstract

**Introduction:**

Rheumatoid arthritis (RA) is a chronic inflammatory and destructive joint disease characterized by overexpression of pro-inflammatory/pro-destructive genes and other activating genes (for example, proto-oncogenes) in the synovial membrane (SM). The gene expression in disease is often characterized by significant inter-individual variances via specific synchronization/desynchronization of gene expression. To elucidate the contribution of the variance to the pathogenesis of disease, expression variances were tested in SM samples of RA patients, osteoarthritis (OA) patients, and normal controls (NCs).

**Method:**

Analysis of gene expression in RA, OA, and NC samples was carried out using Affymetrix U133A/B oligonucleotide arrays, and the results were validated by real-time reverse transcription-polymerase chain reaction. For the comparison between RA and NC, 568 genes with significantly different variances in the two groups (*P *≤ 0.05; Bonferroni/Holm corrected Brown-Forsythe version of the Levene test) were selected. For the comparison between RA and OA, 333 genes were selected. By means of the *Kyoto Encyclopedia of Genes and Genomes*, the pathways/complexes significantly affected by higher gene expression variances were identified in each group.

**Results:**

Ten pathways/complexes significantly affected by higher gene expression variances were identified in RA compared with NC, including cytokine–cytokine receptor interactions, the transforming growth factor-beta pathway, and anti-apoptosis. Compared with OA, three pathways with significantly higher variances were identified in RA (for example, B-cell receptor signaling and vascular endothelial growth factor signaling). Functionally, the majority of the identified pathways are involved in the regulation of inflammation, proliferation, cell survival, and angiogenesis.

**Conclusion:**

In RA, a number of disease-relevant or even disease-specific pathways/complexes are characterized by broad intra-group inter-individual expression variances. Thus, RA pathogenesis in different individuals may depend to a lesser extent on common alterations of the expression of specific key genes, and rather on individual-specific alterations of different genes resulting in common disturbances of key pathways.

## Introduction

Human rheumatoid arthritis (RA) is characterized by chronic inflammation and destruction of multiple joints, perpetuated by an abnormally transformed and invasive synovial membrane (SM), forming the so-called pannus tissue [1]. Many activated cell types contribute to the development and progression of RA. Monocytes/macrophages, dendritic cells, T and B cells, endothelial cells, and synovial fibroblasts are major components of the pannus [2-8] and participate in maintaining joint inflammation, degradation of extracellular matrix (ECM) components, and invasion of cartilage and bone [2,4] as well as fibrosis of the affected joints [9].

The extended analysis of gene expression profiles in RA SM during the last decades has revealed several relevant gene groups affecting development and progression of the disease. Central transcription factors involved as key players in RA pathogenesis are AP-1, NF-κB, Ets-1, and SMADs [10-12]. These factors show binding activity for their cognate recognition sites in the promoters of inflammation-related cytokines (for example, tumor necrosis factor-alpha [TNF-α], interleukin [IL]-1β, and IL-6 [3]) and matrix-degrading enzymes (for example, matrix metalloproteinase [MMP]-1 and MMP-3 [13,14]). The latter contribute to tissue degradation by destruction of ECM components, including aggrecan or collagen type I-IV, X, and XI [15].

The analysis of those comprehensive expression data has become feasible due to the implementation of microarray-based methods [16]. Therefore, a variety of comparisons can be performed, including differences in gene expression among different groups and/or individuals. In contrast to conventional differential gene expression analyses, the determination of inter-individual gene expression variances, often affecting gene expression of members of the same patient/donor group, is generally not considered in rheumatology, although those variances are known to be a characteristic of many diseases. In trisomy 21, for instance, inter-individual expression variances affect a number of tightly regulated genes. In addition, the variances are independent of the respective level of gene expression, and although only a minority of genes are affected, these genes are thought to be involved in the symptoms of trisomy 21 with the highest phenotypical differences [17]. Significant inter-individual expression variances have also been reported to affect the expression of telomerase subunits in malignant glioma [18] as well as protein tyrosine kinases and phosphatases in human basophils in asthma and inflammatory allergy [19]. The latter implies that such alterations may also play an important role within inflammatory diseases, reflected in either synchronization (that is, a loss of inter-individual gene expression variances) or desynchronization (that is, increased inter-individual gene expression variances) of gene expression within a group of different individuals/patients.

In RA, differences in gene expression profiles for specific genes among two subgroups of RA patients have been reported, but within these subgroups, the differences are limited to distinct expression levels without significant intra-subgroup expression variances [12]. To the best of our knowledge, there are as yet no reports on broad intra-group inter-individual gene expression variations among RA patients.

Interestingly, although the majority of reports show expression variances in tissues from patients with different diseases, variances have also been reported in normal tissues (for example, the human retina [20] or human B-lymphoblastoid cells [21]). In contrast to expression variations in diseases, the variations in normal donors are generally limited to a small number of genes (for example, 2.6% in the human retina [20]). To analyze inter-individual mRNA expression variances in RA, the occurrence of gene-specific expression differences in the SM was analyzed using the Bonferroni/Holm corrected Brown-Forsythe version of the Levene test for variance analysis [22-24] on the basis of genome-wide mRNA expression data in RA (n = 12), osteoarthritis (OA) (n = 10), and normal control (NC) (n = 9) synovial tissue.

## Materials and methods

### Patients and tissue samples

SM samples were obtained within 10 minutes following tissue excision upon joint replacement/synovectomy from RA (n = 12) and OA (n = 10) patients at the Department of Orthopedics, University Hospital Jena, Waldkrankenhaus 'Rudolf Elle' (Eisenberg, Germany). Tissue samples from joint trauma surgery (n = 9) were used as NCs (Table 1). After removal, tissue samples were frozen and stored at -70°C. Informed patient consent was obtained and the study was approved by the Ethics Committee of University Hospital Jena (Jena, Germany). RA patients were classified according to the American College of Rheumatology criteria [25], OA patients according to the respective criteria for OA [26].

**Table 1:** Clinical characteristics of the patients at the time of synovectomy/sampling

Patients, total	Gender, male/female	Age, years	Disease duration, years	Rheumatoid factor, +/-	ESR, mm/hour	CRP^a^, mg/L	Number of ARA criteria for RA	Concomitant medication (number)
Rheumatoid arthritis								
12	3/9	65.9 ± 2.9	15.8 ± 4.2	10/2	42.6 ± 6.2	31.9 ± 7.2	5.3 ± 2.1	MTX (5)
								Prednis. (10)
								Sulfas. (3)
								NSAIDs (9)
Osteoarthritis								
10	2/8	71.9 ± 2.0	6.2 ± 2.7	1/9	22.9 ± 4.0	7.6 ± 2.9	0.1 ± 0.1	NSAIDs (4)
								None (7)
Normal controls								
9	7/2	49.9 ± 6.7	0.4 ± 0.3	ND	ND	ND	0.0 ± 0.0	None

^a^Normal range: <5 mg/L. For the parameters of age, disease duration, erythrocyte sedimentation rate (ESR), C-reactive protein (CRP), and number of American Rheumatism Association (ARA) (now American College of Rheumatology) criteria for rheumatoid arthritis (RA), mean ± standard error of the mean is given. For the remaining parameters, numbers are provided. +/-, positive/negative; MTX, methotrexate; ND, not determined; NSAID, nonsteroidal anti-inflammatory drug; Prednis., prednisolone; Sulfas., sulfasalazine.

### Isolation of total RNA

Tissue homogenization, total RNA isolation, treatment with RNase-free DNase I (Qiagen, Hilden, Germany), and cDNA synthesis were performed as described previously [27].

### Microarray data analysis

RNA probes were labeled according to the instructions of the supplier (Affymetrix, Santa Clara, CA, USA). Analysis of gene expression was carried out using U133A/B oligonucleotide arrays. Hybridization and washing procedures were performed according to the supplier's instructions and microarrays were analyzed by laser scanning (Hewlett-Packard Gene Scanner; Hewlett-Packard Company, Palo Alto, CA, USA). Background-corrected signal intensities were determined using the MAS 5.0 software (Affymetrix). Subsequently, signal intensities were normalized among arrays to facilitate comparisons between different patients. For this purpose, arrays were grouped according to patient/donor groups (RA, n = 12; OA, n = 10; and NC, n = 9). The arrays in each group were normalized using quantile normalization [28]. Original data from microarray analyses were deposited in the Gene Expression Omnibus of the National Center for Biotechnology Information (Bethesda, MD, USA) (accession number GSE12021 [29]).

### Real-time reverse transcription-polymerase chain reaction

The data obtained by Affymetrix microarrays were validated for six selected genes (*IL13*, *MAPK8*, *SMAD2*, *IL2RG*, *PLCB1*, and *ATF5*) using real-time reverse transcription-polymerase chain reaction (RT-PCR). PCRs were performed as previously described using a Mastercycler^® ^ep realplex (Eppendorf, Hamburg, Germany) and SYBR-green. To normalize the amount of cDNA in each sample, the expression of the housekeeping gene *GAPDH *(glyceraldehyde 3-phosphate dehydrogenase) was determined [27]. Product specificity was confirmed by (a) melting curve analysis, (b) agarose gel electrophoresis, and (c) cycle sequencing of the PCR products.

### Statistical analysis of gene expression variance

This analysis did not concentrate on differently expressed genes, but on genes with different variances in the three patient groups [30]. The assumption of homogeneity of variance can be rejected by a variance analysis according to Levene [22]. The Brown-Forsythe version of this test was used [23]. For independent groups of data, the null hypothesis (that is, variances are equal) was tested.

To control the stability of the variance, the variance calculation was tested for 2, 3, 5, 7, and 10 samples per group. For fewer than 5 samples, the calculation did not reach stable results, but stable results were achieved for more than 5 patients. In addition, the results of the statistical tests were influenced by the number of samples in each group (that is, small groups did not reach statistical significance).

The *P *value can be obtained by calculating the value of the cumulative distribution function at the point *F*. This is equivalent to the integral of the probability density function of the normal distribution over the interval [0, *F*]. To prevent the accumulation of false-positives due to multiple comparisons, the very strict Bonferroni correction was used [31]. Alternatively, the less conservative Holm correction was applied for the correction of the data [24]. The application of the Holm correction yielded results comparable to those obtained by Bonferroni correction and pointed out only very few new genes.

The variance-fold is defined as the quotient of the variance of one group (for example, OA patients) and the variance of another group (for example, RA patients). If the variance in the second group is higher than 1, the result is the multiplicative inverse and the algebraic sign is inverted. This way, all groups can be compared:

$$VarFold=\{\begin{array}{l} {\mathrm{var}}_{x}\geq {\mathrm{var}}_{y}:{\mathrm{var}}_{x}/{\mathrm{var}}_{y}\\ {\mathrm{var}}_{x}<{\mathrm{var}}_{y}:-1*({\mathrm{var}}_{y}/{\mathrm{var}}_{x}) \end{array}$$

The application of a variance filter before testing of the data (excluding variance-fold values between 2.5 and -2.5 from the analysis) yielded equivalent results compared with the initial data analysis including the *a posteriori *application of the Bonferroni or the Holm correction. Following *Kyoto Encyclopedia of Genes and Genomes *(KEGG) analysis (see below), the same pathways/complexes were indicated and only the ranking of selected pathways/complexes was changed (for example, the ranking of cytokine–cytokine receptor interactions and the mitogen-activated protein kinase [MAPK] pathway were inverted).

### Analysis of inter-individual gene expression variances

Relevant genes were selected using different criteria: (a) a significance level of *P *≤ 0.05 (Bonferroni/Holm corrected Brown-Forsythe version of the Levene test) for variance-fold values and (b) a cutoff value for absolute variance-fold levels of greater than 2.5 for higher variances in RA, OA, and NC, respectively. Using these criteria, 568 genes were selected for the comparison between RA and NC (307 with higher variances in RA and 261 with higher variances in NC) while 542 genes were used for the comparison OA versus NC (314 with higher variances in OA and 228 with higher variances in NC). Finally, 333 genes were selected for the comparison between RA and OA (186 with higher variances in RA and 147 with higher variances in OA). All selected genes are presented in Supplementary Table 1 (sorted according to absolute variance-fold values). Inter-individual variances of gene expression among the different groups were analyzed using predefined pathways and functional categories annotated by KEGG [32].

### Mapping of probesets onto gene names

Gene names used for KEGG inputs follow the nomenclature of the HUGO Genome Nomenclature Committee [33] and are mostly derived from the Affymetrix annotation feature 'Gene Symbol' for the respective probeset. If required, corresponding RefSeqs were manually inspected.

### Statistical KEGG analysis

To ensure that only KEGG pathways with a significant enrichment of more variant genes were obtained for further analyses, the χ^2 ^test statistic was used. Following the calculation of the expected frequency of affected genes in each pathway, the difference between the expected frequency and the absolute frequency was determined. All pathways with a difference of less than 2 were ignored. As a second criterion of the multilevel test, *P *values of less than or equal to 0.15 were considered statistically significant [34]. Pathways with insignificant *P *values were examined in detail and subdivided into two or more sub-pathways if possible. In some cases, *P *values for selected sub-pathways decreased considerably.

## Results

### Analysis of inter-individual gene expression variances in rheumatoid arthritis, osteoarthritis, and normal control synovial membrane

**Figure 1:**
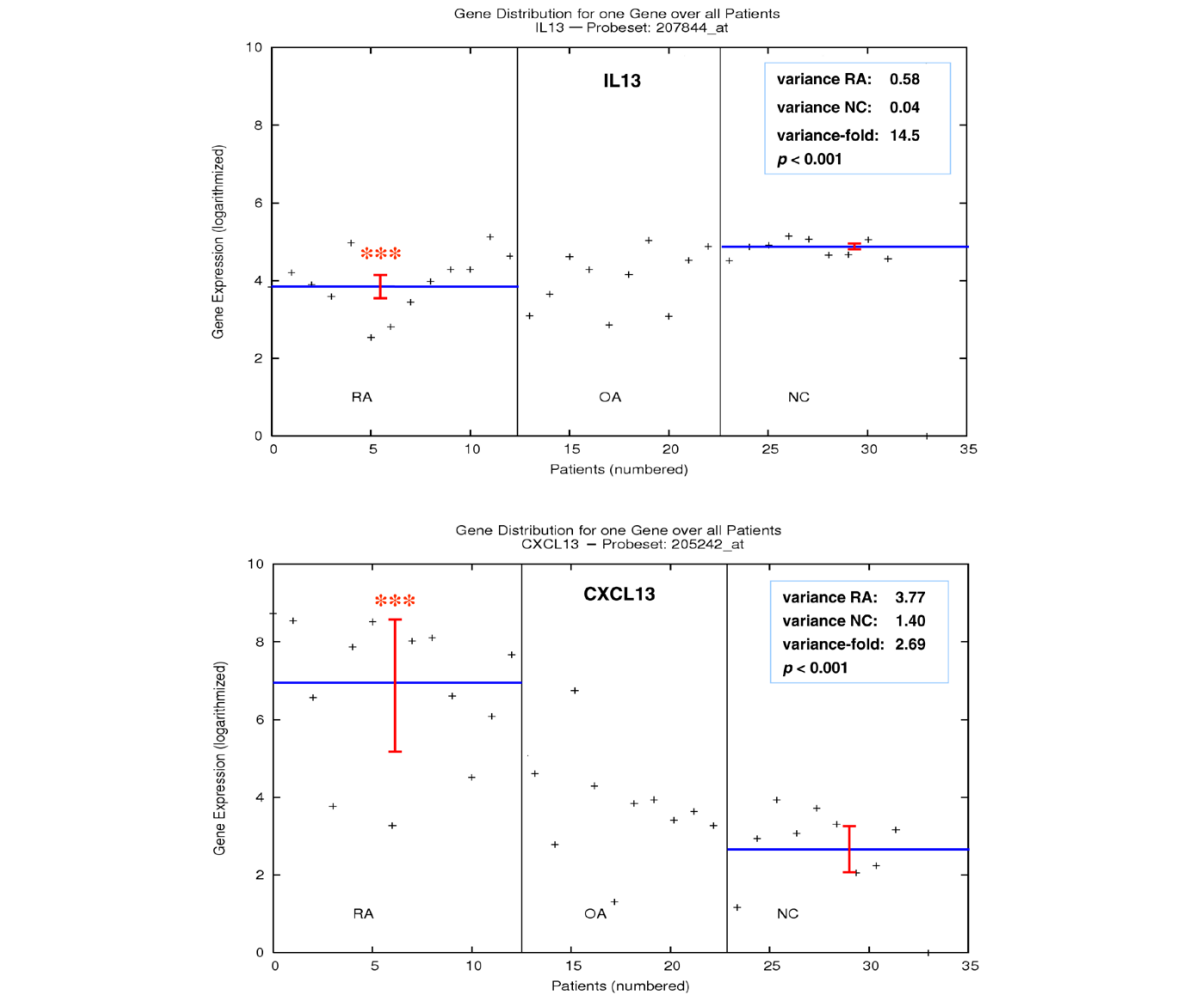
Gene-specific inter-individual gene expression variances. The graph shows the individual gene expression level of rheumatoid arthritis (RA) (n = 12) and osteoarthritis (OA) (n = 10) patients as well as normal control (NC) donors (n = 9) for *IL13 *and *CXCL13 *(cytokine–cytokine receptor interactions). The mean gene expression (blue line) and the intra-group inter-individual variances in RA and NC synovial membrane (red bar) are indicated, resulting in significantly enhanced variances among patients within the RA group (*P *< 0.001, Bonferroni/Holm corrected Brown-Forsythe version of the Levene test).

For the comparison of inter-individual gene expression variances between RA SM (n = 12) and NC SM (n = 9), 568 genes were used (307 with significantly higher variances in RA and 261 with significantly higher variances in NC; *P *≤ 0.05, Bonferroni/Holm corrected Brown-Forsythe version of the Levene test), resulting in the identification of 129 affected KEGG pathways/complexes in total (Supplementary Table 1a; shown for *IL13 *and *CXCL13 *in Figure 1). These pathways include 10 pathways significantly affected by higher gene expression variances in RA and 6 pathways significantly affected by higher gene expression variances in NC (in both cases *P *≤ 0.15, χ^2 ^test).

For the comparison of OA (n = 10) and NC (n = 9) SM, 542 genes were used (314 with significantly higher variances in OA and 228 with significantly higher variances in NC; Supplementary Table 1b). A total of 128 affected KEGG pathways/complexes were identified, including 7 pathways significantly affected by higher gene expression variances in OA and 4 pathways significantly affected by higher gene expression variances in NC.

The comparison of RA (n = 12) and OA (n = 10) SM was performed with 333 genes (186 with significantly higher variances in RA and 147 with significantly higher variances in OA; Supplementary Table 1c). This comparison culminated in the identification of 114 pathways, 3 of which were significantly affected by higher gene expression variances in RA and 4 of which were significantly affected by higher gene expression variances in OA.

### Real-time reverse transcription-polymerase chain reaction validation

Validation of the microarray data by real-time RT-PCR was attempted in RA, OA, and NC samples for the genes *IL13*, *MAPK8*, *SMAD2*, *IL2RG*, *PLCB1*, and *ATF5*. In three cases (50%), the results of microarray analyses and real-time RT-PCR were equivalent for RA versus NC *(MAPK8*: variance-fold 9.8 versus 5.2; *IL2RG*: variance-fold 5.6 versus 8.9; *ATF5*: variance-fold 1.7 versus 2.3); in addition, two cases (33%) tended to result in comparable variance-fold values for microarray and real-time RT-PCR (*IL13*: variance-fold 12 versus 1.3; *SMAD2*: variance-fold 5 versus 1.1). In only one case (*PLCB1*; 17%), microarray analyses and real-time RT-PCR validation showed contradictory results (higher variance in NC versus higher variance in RA). For OA versus NC, comparable results were achieved (only *IL2RG *and *ATF5 *showed contradictory results).

### KEGG pathways identified in the comparison between rheumatoid arthritis and normal control

#### Pathways significantly affected by inter-individual gene expression variances in rheumatoid arthritis

**Figure 2:**
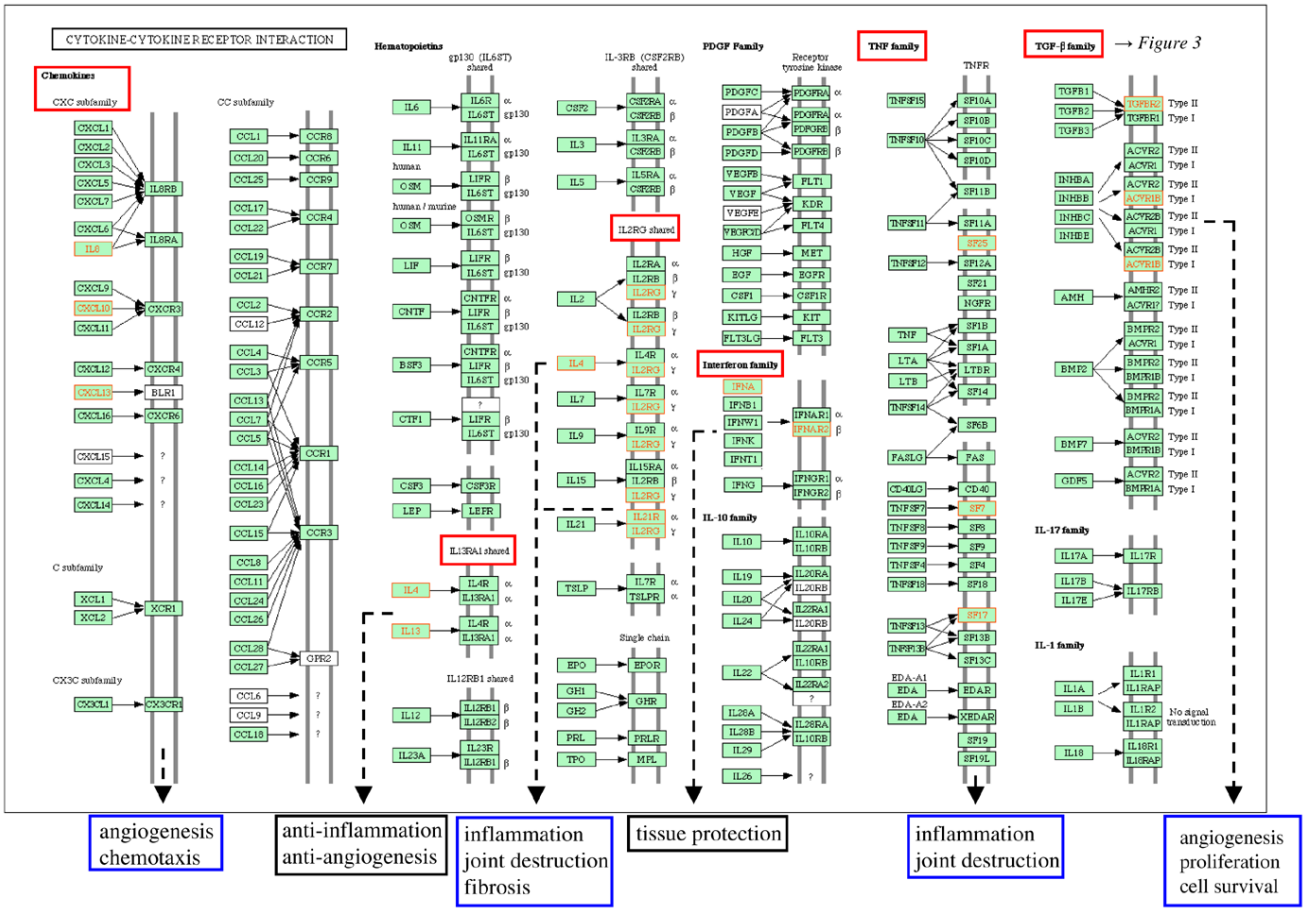
Inter-individual mRNA expression variances among cytokine–cytokine receptor interactions in rheumatoid arthritis (RA) compared with normal control (NC). The graph shows genes affected by significant intra-group inter-individual mRNA expression variances in RA compared with NC (*P *≤ 0.05; Bonferroni/Holm corrected Brown-Forsythe version of the Levene test; labeled in red) among *Kyoto Encyclopedia of Genes and Genomes *(KEGG) cytokine–cytokine receptor interactions, including the respective sub-pathways (*P *≤ 0.15, χ^2 ^test; labeled in red). Cellular processes with potential influence on or relevance for RA pathogenesis (for example, inflammation, proliferation, and cell survival) are labeled in blue, and anti-inflammatory/anti-destructive processes are labeled in black.

**Figure 3:**
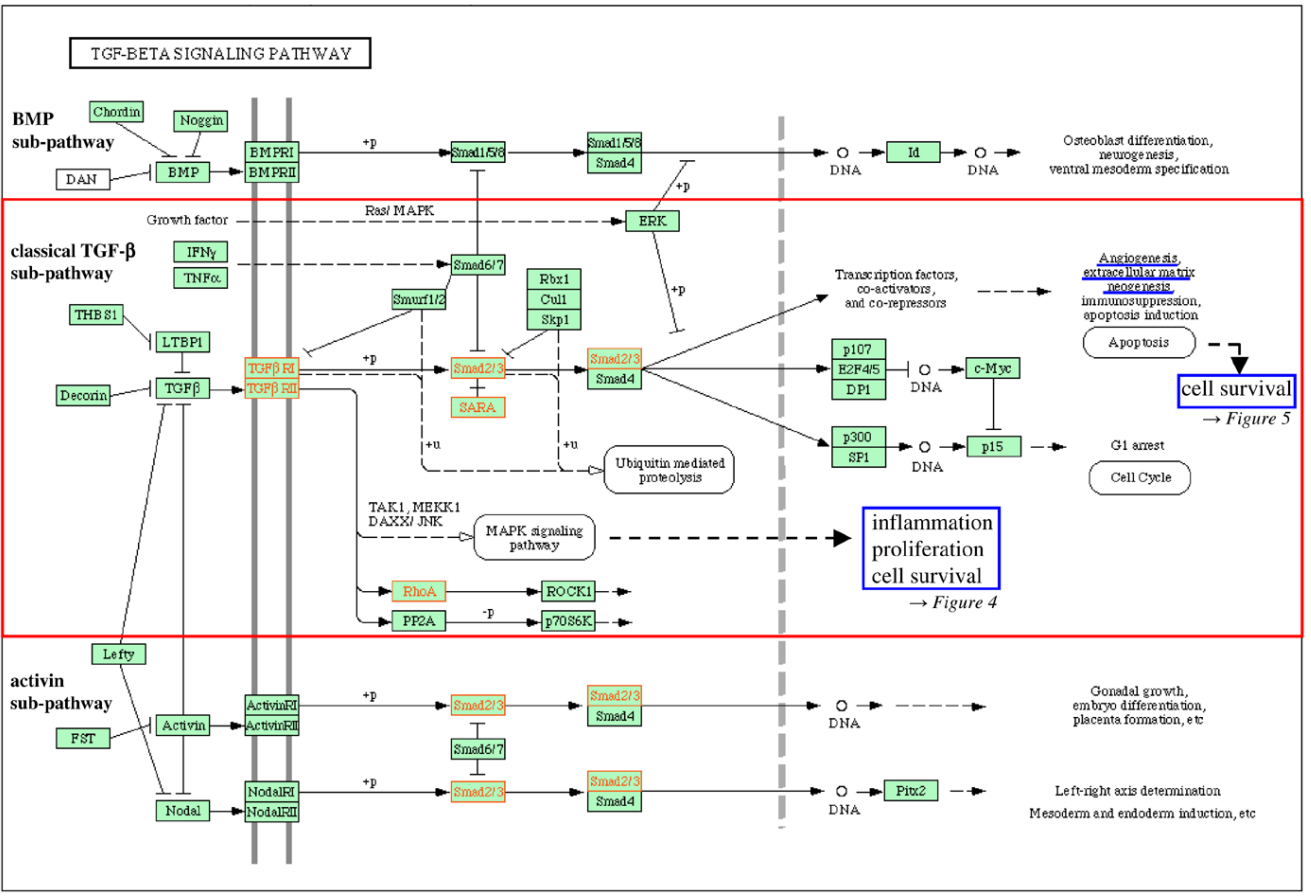
Inter-individual mRNA expression variances in the transforming growth factor-beta (TGF-β) signaling pathway in rheumatoid arthritis (RA) compared with normal control (NC). The graph shows genes affected by significant intra-group inter-individual mRNA expression variances in RA compared with NC (*P *≤ 0.05; Bonferroni/Holm corrected Brown-Forsythe version of the Levene test; labeled in red) in the *Kyoto Encyclopedia of Genes and Genomes *(KEGG) TGF-β signaling pathway. Among the three TGF-β family sub-pathways, the classical TGF-β sub-pathway is significantly affected by gene expression variances (*P *≤ 0.15, χ^2 ^test; indicated in red). TGF-β-regulated cellular processes with potential influence on or relevance for RA pathogenesis (for example, angiogenesis and cell survival) are labeled in blue.

**Figure 4:**
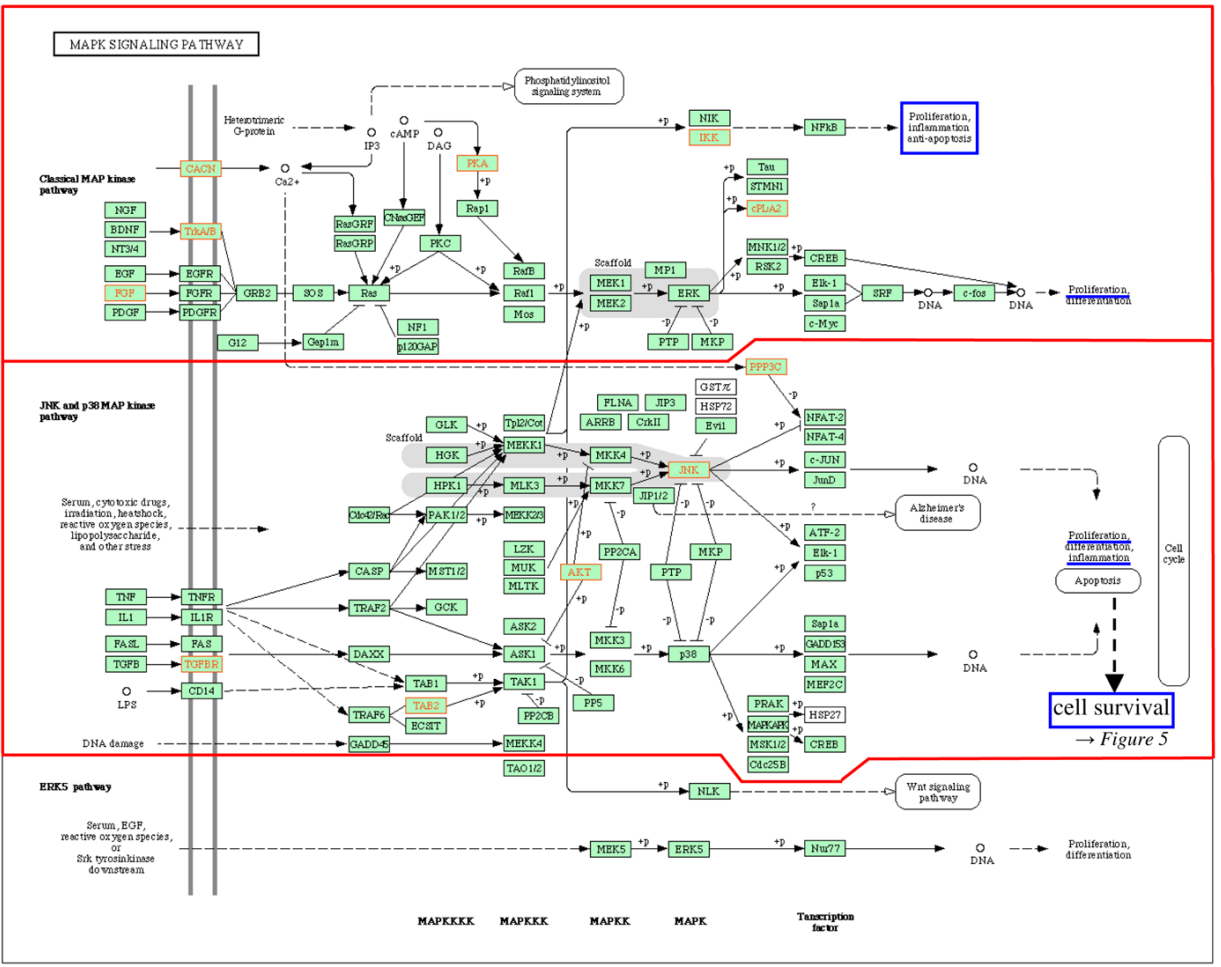
Inter-individual mRNA expression variances in the mitogen-activated protein kinase (MAPK) signaling pathway in rheumatoid arthritis (RA) compared with normal control (NC). The graph shows genes affected by significant intra-group inter-individual mRNA expression variances in RA compared with NC (*P *≤ 0.05; Bonferroni/Holm corrected Brown-Forsythe version of the Levene test; labeled in red) in the *Kyoto Encyclopedia of Genes and Genomes *(KEGG) MAPK signaling pathway. Among the three MAPK family sub-pathways, the classical and the c-jun kinase (JNK)/p38 MAPK sub-pathways were significantly affected by gene expression variances (*P *≤ 0.15, χ^2 ^test; indicated in red). MAPK-regulated cellular processes with potential influence on or relevance for RA pathogenesis (for example, proliferation, inflammation, and anti-apoptosis) are labeled in blue.

**Figure 5:**
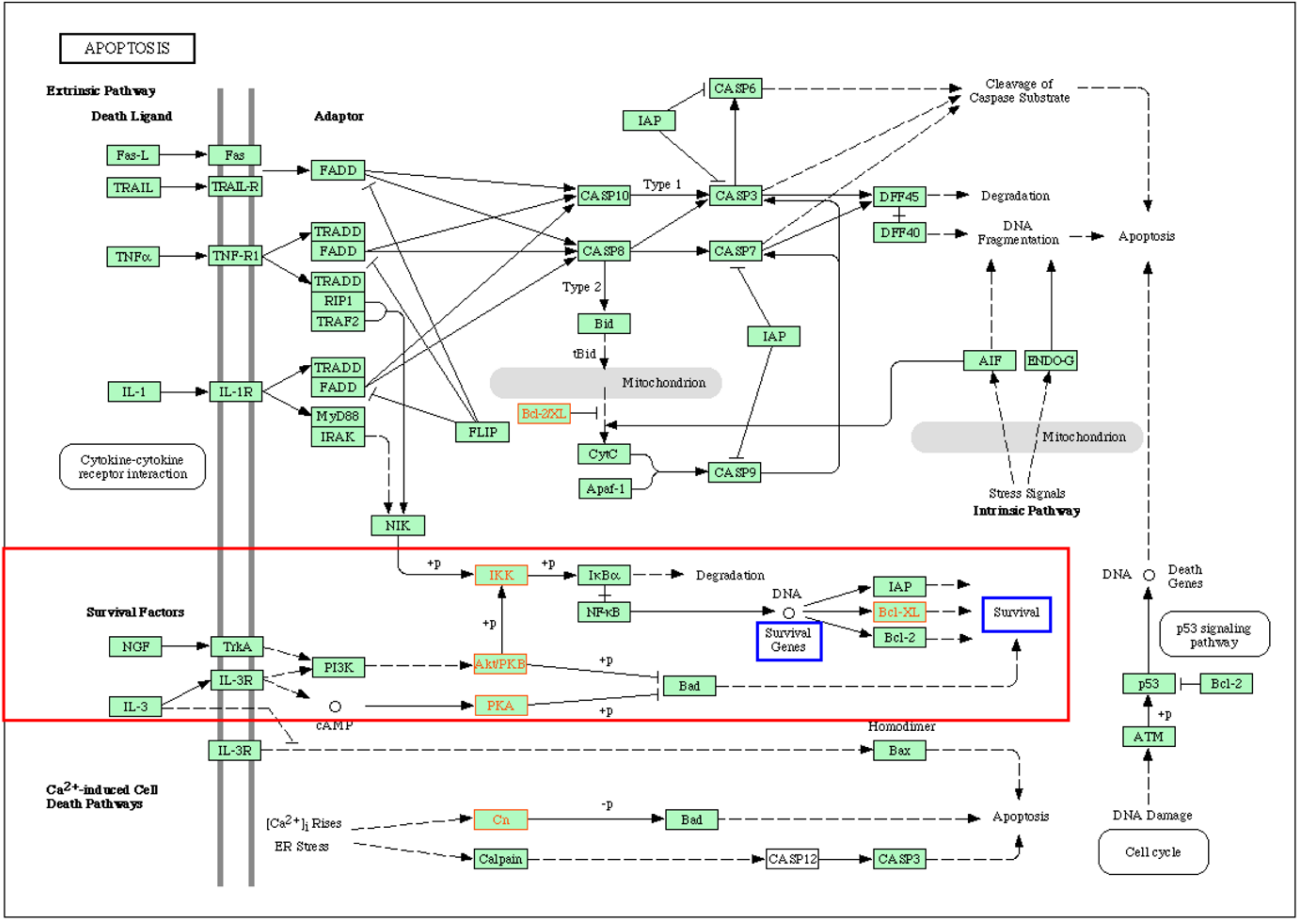
Inter-individual mRNA expression variances in the complex of apoptosis in rheumatoid arthritis (RA) compared with normal control (NC). The graph shows genes affected by significant intra-group inter-individual mRNA expression variances in RA compared with NC (*P *≤ 0.05; Bonferroni/Holm corrected Brown-Forsythe version of the Levene test; labeled in red) in the *Kyoto Encyclopedia of Genes and Genomes *(KEGG) complex of apoptosis. Among the three apoptosis sub-complexes, the survival factor-dependent sub-complex was significantly affected by gene expression variances (*P *≤ 0.15, χ^2 ^test; indicated in red). Cellular processes with potential influence on or relevance for RA pathogenesis (expression of survival genes and cell survival) are labeled in blue.

Ten pathways/complexes significantly affected by inter-individual mRNA expression variances were identified in the comparison between RA and NC, 7 of which were specific for RA, that is, did not appear in the comparison between OA and NC (for example, cytokine–cytokine receptor interactions; Figure 2). The occurrence of gene expression variances in the complete MAPK, transforming growth factor-beta (TGF-β), and apoptosis pathways/complexes did not reach statistical significance. Interestingly, within these pathways, significantly affected sub-pathways/sub-complexes could be identified: the classical TGF-β sub-pathway (Figure 3), the classical and the c-jun kinase (JNK)/p38 MAPK sub-pathway(s) (Figure 4), and the sub-complex of anti-apoptosis (Figure 5). A complete list of significantly affected pathways/complexes is presented in Table 2.

**Table 2:** KEGG pathways/complexes significantly affected by intra-group inter-individual gene expression variance in rheumatoid arthritis (RA) compared with normal control (that is, higher variances in RA)

	KEGG identification number	Pathway/complex	B (E)	χ^2^	*P *value	Affected genes
1	hsa04060	Cytokine–cytokine receptor interaction^a^	14 (8)	4.56	0.12	*CXCL13, IFNA8, FNAR2, IL2RG, IL4, IL8, IL13, CXCL10, IL21R, TNFRSF17, TGFBR2, CD27, TNFRSF25, ACVR1B*
2	hsa04010	MAPK signaling pathway^a^	13 (8)	3.32	0.22	*CHP, AKT2, MAP3K7IP2, PLA2G2D, IKBKB, NTRK2, PRKACA, MAPK8, PRKX, TGFBR2, CACNB1, FGF18, ACVR1B*
2a	hsa04010	MAPK signaling pathway^a ^(classical + JNK/p38 MAPK sub-pathway)	13 (7)	4.39	0.13	*CHP, AKT2, MAP3K7IP2, PLA2G2D, IKBKB, NTRK2, PRKACA, MAPK8, PRKX, TGFBR2, CACNB1, FGF18, ACVR1B*
3	hsa05212	Pancreatic cancer^a^	9 (2)	20.29	<0.01	*E2F3, AKT2, IKBKB, SMAD2, MAPK8, BCL2L1, STAT1, TGFBR2, ACVR1B*
4	hsa04620	Toll-like receptor signaling pathway^a^	9 (3)	14.01	<0.01	*AKT2, MAP3K7IP2, IFNA8, IFNAR2, IKBKB, IL8, CXCL10, MAPK8, STAT1*
5	hsa04660	T-cell receptor signaling pathway^a^	7 (3)	5.98	0.05	*CHP, AKT2, IKBKB, IL4, RHOA, PDK1, PLCG1*
6	hsa04664	Fc epsilon receptor I signaling pathway^a^	7 (2)	9.53	0.01	*AKT2, PLA2G2D, IL4, IL13, PDK1, PLCG1, MAPK8*
7	hsa04520	Adherens junction^a^	6 (2)	5.56	0.07	*CSNK2A1, RHOA, SMAD2, TGFBR2, ACVR1B, CDH1*
8	hsa05220	Chronic myeloid leukemia^a^	6 (2)	5.73	0.06	*E2F3, IKBKB, BCL2L1, TGFBR2, ACVR1B, AKT2*
9	hsa04350	TGF-β signaling pathway^a^	5 (3)	1.86	0.38	*RHOA, SMAD2, TGFBR2, ACVR1B, ZFYVE9*
9a	hsa04350	TGF-β signaling pathway^a ^(classical TGF-β sub-pathway)	5 (2)	6.7	0.05	*RHOA, SMAD2, TGFBR2, ACVR1B, ZFYVE9*
10	hsa04210	Apoptosis^a^	5 (3)	2.25	0.34	*AKT2, IKBKB, PRKACA, BCL2L1, CHP*
10a	hsa04210	Apoptosis^a ^(anti-apoptotic sub-complex)	5 (1)	6.7	0.03	*AKT2, IKBKB, PRKACA, BCL2L1, CHP*

^a^Specifically affected in rheumatoid arthritis. B, absolute frequency; E, expected frequency; JNK, c-jun kinase; KEGG, *Kyoto Encyclopedia of Genes and Genomes*; MAPK, mitogen-activated protein kinase; TGF-β, transforming growth factor-beta.

#### Pathways significantly affected by inter-individual gene expression variances in normal control

Six pathways/complexes significantly affected by inter-individual mRNA expression variances were identified in NC compared with RA, including the cell cycle and the Wnt (wingless-type MMTV integration site family) signaling pathway. All pathways/complexes were specific for NC. A complete list of significantly affected pathways/complexes is presented in Table 3.

**Table 3:** KEGG pathways/complexes significantly affected by intra-group inter-individual gene expression variance in normal control (NC) compared with rheumatoid arthritis (that is, higher variances in NC)

	KEGG identification number	Pathway/complex	B (E)	χ^2^	*P *value	Affected genes
1	hsa03010	Ribosome^a^	8 (3)	27.62	<0.01	*RPL7, RPL9, RPL21, RPL27, RPL30, RPS6, RPS10, RPS12*
2	hsa04110	Cell cycle^a^	7 (4)	13.11	<0.01	*CDKN1A, E2F1, GADD45B, ATM, SKP1A, CCNA2, CDC2*
3	hsa04310	Wnt signaling pathway^a^	7 (5)	7.8	0.01	*CACYBP, PPP2R1B, PRKACB, PSEN1, SKP1A, TBL1XR1, FZD1*
4	hsa04640	Hematopoietic cell lineage^a^	4 (3)	4.15	0.15	*CSF1, EPOR, FLT3LG, ITGA4*
5	hsa05010	Alzheimer disease^a^	3 (1)	13.3	<0.01	*GAPDH, LRP1, PSEN1*
6	hsa01510	Neurodegenerative disorders^a^	3 (1)	8.18	0.01	*GAPDH, NR4A2, PSEN1*

^a^Specifically affected in rheumatoid arthritis. B, absolute frequency; E, expected frequency; KEGG, *Kyoto Encyclopedia of Genes and Genomes*; Wnt, wingless-type MMTV integration site family.

### KEGG pathways identified in the comparison between osteoarthritis and normal control

#### Pathways significantly affected by inter-individual gene expression variances in osteoarthritis

Seven pathways/complexes significantly affected by inter-individual mRNA expression variances were identified in OA compared with NC. Among these pathways/complexes, six were specific for OA, including the complexes of apoptosis. A complete list of significantly affected pathways/complexes is presented in Table 4.

**Table 4:** KEGG pathways/complexes significantly affected by intra-group inter-individual gene expression variance in osteoarthritis (OA) compared with normal control (that is, higher variances in OA)

	KEGG identification number	Pathway/complex	B (E)	χ^2^	*P *value	Affected genes
1	hsa04310	Wnt signaling pathway	7 (4)	3.44	0.21	*CSNK2A1, SMAD2, PPP3CB, PRKACA, TBL1X, BTRC, RBX1*
1a	hsa04310	Wnt signaling pathway (canonical sub-pathway)	6 (3)	4.56	0.12	*CSNK2A1, BTRC, SMAD2, PRKACA, TBL1X, RBX1*
2	hsa04210	Apoptosis^a^	6 (2)	8.13	0.01	*AKT2, IKBKB, PP3CB, PRKACA, RKAR2A, BCL2L*
3	hsa03010	Ribosome^a^	5 (2)	3.99	0.16	*RPL18, RPL35A, RPL38, RPS10, RPL14*
3a	hsa03010	Ribosome^a ^(large subunit)	4 (1)	6.49	0.04	*RPL18, RPL35A, RPL38, RPL14*
4	hsa04520	Adherens junction^a^	5 (2)	5.57	0.07	*CSNK2A1, SMAD2, ACP1, TGFBR2, YES1*
5	hsa05212	Pancreatic cancer^a^	5 (1)	6.22	0.04	*AKT2, IKBKB, SMAD2, BCL2L1, TGFBR2*
6	hsa04120	Ubiquitin-mediated proteolysis^a^	4 (2)	8.12	0.01	*ANAPC5, UBE2D2, BTRC, RBX1*
7	hsa05050	Dentatorubropallidoluysian atrophy^a^	3 (1)	19.79	<0.01	*ATN1, RERE, MAGI1*

^a^Specifically affected in rheumatoid arthritis. B, absolute frequency; E, expected frequency; KEGG, *Kyoto Encyclopedia of Genes and Genomes*; Wnt, wingless-type MMTV integration site family.

#### Pathways significantly affected by inter-individual gene expression variances in normal control

Four pathways/complexes significantly affected by inter-individual mRNA expression variances were identified in NC compared with OA. Three of those were specific for NC, including the Toll-like receptor signaling pathway. A complete list of significantly affected pathways/complexes is presented in Table 5.

**Table 5:** KEGG pathways/complexes significantly affected by intra-group inter-individual gene expression variance in normal control (NC) compared with osteoarthritis (that is, higher variances in NC)

	KEGG identification number	Pathway/complex	B (E)	χ^2^	*P *value	Affected genes
1	hsa04310	Wnt signaling pathway	8 (3)	6.55	0.04	*CSNK1A1, DKK2, JUN, MYC, PPP2R1B, PRKACB, WNT5B, FZD1*
2	hsa05120	Epithelial cell signaling in *Helicobacter pylori *infection^a^	5 (2)	7.97	0.01	*JUN, NFKBIA, ATP6V1C1, ADAM17, ATP6V0D1*
3	hsa05211	Renal cell carcinoma^a^	5 (2)	7.75	0.01	*AKT2, HGF, JUN, TCEB1, VEGFA*
4	hsa04620	Toll-like receptor signaling pathway^a^	5 (2)	4.43	0.12	*AKT2, JUN, NFKBIA, TLR7, STAT1*

^a^Specifically affected in rheumatoid arthritis. B, absolute frequency; E, expected frequency; KEGG, *Kyoto Encyclopedia of Genes and Genomes*; Wnt, wingless-type MMTV integration site family.

### KEGG pathways identified in the comparison between rheumatoid arthritis and osteoarthritis

#### Pathways significantly affected by inter-individual gene expression variances in rheumatoid arthritis

Three pathways/complexes significantly affected by inter-individual mRNA expression variances were identified in RA compared with OA. All pathways/complexes were specific for RA, including the vascular endothelial growth factor (VEGF) and the B-cell receptor signaling pathways. A complete list of significantly affected pathways/complexes is presented in Table 6.

**Table 6:** KEGG pathways/complexes significantly affected by intra-group inter-individual gene expression variance in rheumatoid arthritis (RA) compared with osteoarthritis (that is, higher variances in RA)

	KEGG identification number	Pathway/complex	B (E)	χ^2^	*P *value	Affected genes
1	hsa04916	Melanogenesis^a^	6 (3)	6.53	0.03	*ADCY2, LEF1, PRKCB1, PRKX, TCF7, WNT8B*
2	hsa04662	B-cell receptor signaling pathway^a^	5 (2)	9.72	0.01	*MALT1, PIK3CD, PLCG2, PRKCB1, CD72*
3	hsa04370	VEGF signaling pathway^a^	4 (2)	4.09	0.15	*PLA2G2D, PIK3CD, PLCG2, PRKCB1*

^a^Specifically affected in rheumatoid arthritis. B, absolute frequency; E, expected frequency; KEGG, *Kyoto Encyclopedia of Genes and Genomes*; VEGF, vascular endothelial growth factor.

#### Pathways significantly affected by inter-individual gene expression variances in osteoarthritis

Four pathways/complexes significantly affected by inter-individual mRNA expression variances were identified in OA compared with RA (for example, the complex of oxidative phosphorylation). All of them were specific for OA. A complete list of significantly affected pathways/complexes is presented in Table 7.

**Table 7:** KEGG pathways/complexes significantly affected by intra-group inter-individual gene expression variance in osteoarthritis (OA) compared with rheumatoid arthritis (that is, higher variances in OA)

	KEGG identification number	Pathway/complex	B (E)	χ^2^	*P *value	Affected genes
1	hsa00190	Oxidative phosphorylation^a^	10 (1)	75.6	<0.01	*COX5B, NDUFA6, NDUFA8, NDUFB2, NDUFB4, SDHC, NDUFB6, aNDUFC1, NDUFA13, ATP5G3*
2	hsa04010	MAPK signaling pathway^a^	5 (2)	3.8	0.17	*DUSP5, RASGRP3, FAS, MAPK11, TAOK1*
2a	hsa04010	MAPK signaling pathway^a ^(JNK/p38 MAPK sub-pathway)	4 (1)	6.54	0.03	*DUSP5, FAS, MAPK11, TAOK1*
3	hsa00790	Folate biosynthesis^a^	3 (0)	22.03	<0.01	*ASCC3, SETX, SMARCA5*
4	hsa00500	Starch and sucrose metabolism^a^	3 (1)	7.86	0.01	*ASCC3, SETX, SMARCA5*

^a^Specifically affected in rheumatoid arthritis. B, absolute frequency; E, expected frequency; JNK, c-jun kinase; KEGG, *Kyoto Encyclopedia of Genes and Genomes*; MAPK, mitogen-activated protein kinase.

## Discussion

The present microarray-based and real-time RT-PCR-validated, genome-wide mRNA expression analysis in RA, OA, and NC SM by KEGG mapping shows that gene-specific, significant, intra-group/inter-individual variances in gene expression profiles occur in RA. These variances affect a variety of genes involved in numerous pathways/complexes potentially relevant for RA pathogenesis. Since significant variance-fold values are observed for many genes with comparable mean expression levels among different patient/donor groups (data not shown), the manifestation of gene expression variances does not necessarily depend on the respective mean mRNA expression level.

To our knowledge, gene expression variances in RA samples have been reported only for distinct subgroup-specific differences in gene expression profiles of RA patients [12]. Consequently, the present data demonstrate for the first time broad intra-group/inter-individual gene expression variances in RA SM samples, previously observed in other severe diseases such as trisomy 21, malignant glioma, and inflammatory allergy [17-19]. It has been hypothesized that expression variances of regulatory key genes contribute to the individual phenotype of the given disease [17], whether independent of or depending on the expression level.

Currently, the causes for gene expression variances among RA patients are unknown. Possible external reasons may include the higher average age of the individuals in the RA group as well as medication influencing immunological processes and the expression of immunologically relevant genes (for example, methotrexate, prednisolone, sulfasalazine, and/or nonsteroidal anti-inflammatory drugs [35,36]) or differences in nutrition, with general effects on individual gene expression [37]. The inflammatory status of the respective joint at the time of surgical intervention may also substantially influence gene expression in the RA SM [38]. However, an analysis of the differential gene expression shows that the present RA group is generally characterized by an expression profile highly compatible with previous gene expression studies [39], including the overexpression of several transcription factors (for example, *FOS*, *FOSB*, *JUN*, and *STAT1 *[10-12]), cytokines/chemokines (for example, *IL2*, *IL4*, *CCL23*, and *CCL25 *[40]), signal transduction molecules (for example, *MAPK9*, *MAP3K2*, *PTPN7*, and *AKT2 *[41,42]), cell cycle regulators (for example, *CDC12*, *CCNB2*, and *CCNE2 *[43]), and heat shock proteins (DNAJ molecules; [44]; data not shown), indicating that the present RA cohort is representative for RA patients in general.

Regarding internal molecular changes in the individuals, a participation of mutations or single nucleotide polymorphisms in different genes is plausible, either directly [45,46] or via mutated regulators (for example, transcription factors, mRNA stability modifiers, and so on [47]). This also includes broader genomic rearrangements (for example, chromosomal translocations or polysomies [48,49]) as well as epigenomic modifications (for example, gene/promoter methylation [50]). In addition, the individual composition of cell types in the analyzed SM samples may influence the mRNA expression profile, depending on the inflammatory status and/or cell proliferation, potentially resulting in enhanced immigration/proliferation of T cells, B cells, or synovial fibroblasts [51].

In RA compared with NC, 10 KEGG pathways/complexes are specifically and significantly affected by gene expression variances. As expected, the importance of immunological processes for RA progression [8] is reflected in several pathways directly involved in such networks (Toll-like, T cell, and Fc ε receptor signaling [52-54]). In the SM, alterations in immunological pathways/complexes may contribute to the development of local (and systemic) inflammation, reflecting the highly inflamed status of the joint as one of the major characteristics of RA [2,55].

RA-specific gene expression variances also occur in cytokine–cytokine receptor interactions. Within this complex, a striking involvement of sub-pathways can be observed, with relevance for chemotaxis (CXC family chemokines [56]), angiogenesis, proliferation, and cell survival (TGF-β family [57,58]) as well as inflammation, joint destruction, and fibrosis (TNF family [59,60] and IL2RG shared pathway [9,61]; Figure 2). Sub-pathways influencing tissue protection (interferon family [62]) or anti-inflammation and anti-angiogenesis (IL13RA1 [interleukin-13 receptor alpha-1] shared pathway [63]) are scarcely affected. Therefore, a specific influence of gene expression variances on cytokine-mediated aspects of the RA can be assumed [64].

Although the following pathways/complexes are not significantly affected by gene expression variances in total, embedded sub-pathways include the majority of affected genes, thus reaching statistical significance. In the TGF-β pathway, only members of the classical TGF-β sub-pathway are significantly affected, thus potentially influencing angiogenesis [58], cell survival [65], and cell proliferation [66] amongst others (Figure 3). Indeed, this (sub-) pathway appears to occupy a central position for the RA pathogenesis, due to the integration of various RA-relevant cellular functions. This is further underlined by its prominent role within the framework of cytokine–cytokine receptor interactions (Figure 2) and its influence on pro-inflammatory/pro-destructive features, either independent of or via MAPK (Figures 3 and 4). Within the MAPK signaling pathway, the 'classical' and the JNK/p38 MAPK sub-pathways – regulating proliferation, anti-apoptosis, and inflammation – are significantly affected by gene expression variances (Figure 4). This may be an indication of a participation of variable gene expression in inflammatory processes via MAPK variants (especially via *JNK/MAPK8 *[67]) and proliferation of activated cells (for example, synovial fibroblasts and T cells) in RA [68,69] and MAPK-mediated anti-apoptosis (Figure 4).

Regarding apoptosis, genes particularly involved in the regulation of cell survival and anti-apoptosis are significantly affected by expression variances (Figure 5) [70]. Interestingly, the respective genes in this particular pathway also show increased expression levels in RA SM (data not shown). Pro-apoptotic genes are not affected in this pathway, corresponding to the absence of gene expression variances within the complex of p53-induced apoptosis (data not shown).

Depending on the individual gene expression level in each patient, gene expression variances in regulatory pathways may lead to enhanced inflammation [53,54], angiogenesis [71,72], enhanced collagen synthesis and secretion [9], and/or a reduced rate of apoptosis [73], thus potentially contributing to hyperplasia of the SM [74], collagen-dependent fibrosis of the joints [64], and a prolonged life span of activated synovial cells in RA [73,75].

Since RA and OA samples share many aspects of their respective mRNA expression profiles [76,77], genes in a number of pathways show comparable variance-fold values in both RA and OA (for example, apoptosis; Tables 2 and 4), thus reflecting basic similarities of joint diseases. However, RA and OA SM samples can be clearly differentiated regarding gene expression variances in other pathways/complexes. In OA, the pathways/complexes affected by higher expression variances than in NC indicate an OA-specific desynchronization of metabolic processes (Table 7). In contrast, RA-specific pathways/complexes are involved in the regulation of VEGF-mediated angiogenesis [74,75] and vascular permeability [78], as well as B cell-dependent auto-immunity and inflammation [79]. The latter represents the elevated activity status of B cells (including cytokine production and T-cell activation) and – in connection with the affection of the anti-apoptotic sub-pathway – the enhanced survival of self-reactive B cells [5,6,80]. This may result in a pronounced role of B cells for disease development in RA compared with OA, which is also reflected in the increasing impact of B cell-directed treatment in RA [81].

In summary, these pathways indicate limited but distinct molecular/cellular differences between RA and OA and demonstrate a major contribution of inflammation and angiogenesis in RA. It is reasonable to assume that the RA pathogenesis is influenced by broad alterations of gene expression in general. For years, only differential gene expression analyses have been performed, resulting in the identification of some key genes but leading to the disregard of several genes with a more limited influence on RA, whose collective influence may still be as large as that of the already-known key players. Therefore, besides ubiquitous elevated expression levels of exceptional pro-inflammatory/pro-destructive key regulators/mediators like TNF-α, IL-1β [82], or MMP-1 [83], elevated or reduced expression levels of many different genes in various pathways/complexes may also influence RA development and progression. In this process, the affection of pathologically relevant pathways with differentially expressed genes may be more important than the character of the respective genes, resulting in different gene expression profiles among individual RA patients as reflected in the gene expression variances of the present study. As a consequence, synchronized or desynchronized gene expression in RA potentially shifts cellular activity from the normal to an activated status.

Regarding diagnosis and therapy of RA, the present results indicate that a more individualized approach for different patients may represent the future of RA treatment. Thus, the determination of individual gene expression patterns may facilitate the selection of the best medication or, more ambitiously, may allow directed modulation of (individually) selected pathways/complexes instead of broad suppression of inflammation by anti-inflammatory/anti-rheumatic drugs [84]. In addition, the present study helped to identify the TGF-β pathway as an accessory key player in RA, due to its central position within the regulatory networks. This suggestion is strongly supported by an emerging number of publications reporting a decisive impact of TGF-β on RA development/progression [57,58,85,86]. The affected pathways (and the respective genes) reported here may provide the basis for further analyses of the RA pathogenesis and the differences between RA and OA on a cellular and molecular level.

## Conclusion

In RA, a number of disease-relevant or even disease-specific KEGG pathways/complexes (for example, TGF-β signaling and anti-apoptosis) are characterized by broad intra-group inter-individual expression variances. This indicates that RA pathogenesis in different individuals may depend to a lesser extent on common alterations of the expression of specific key genes, and rather on individual-specific alterations of different genes resulting in common disturbances of key pathways. Numerous affected pathways, including TGF-β signaling in a central position, are involved in inflammation, angiogenesis, proliferation, and cell survival, thus potentially influencing characteristic features of RA pathology.

## Abbreviations

ECM: extracellular matrix; IL: interleukin; IL2RG: interleukin 2 receptor gamma; JNK: c-jun kinase; KEGG: *Kyoto Encyclopedia of Genes and Genomes*; MAPK: mitogen-activated protein kinase; MMP: matrix metalloproteinase; NC: normal control; OA: osteoarthritis; PCR: polymerase chain reaction; RA: rheumatoid arthritis; RT-PCR: reverse transcription-polymerase chain reaction; SM: synovial membrane; TGF-β: transforming growth factor-beta; TNF: tumor necrosis factor; VEGF: vascular endothelial growth factor.

## Competing interests

The authors declare that they have no competing interests.

## Authors' contributions

RH performed the KEGG analyses, contributed to the real-time RT-PCR analyses, and participated in the writing of the manuscript. CH analyzed the microarray data, performed the bioinformatic analyses, and participated in the writing of the manuscript. RH and CH contributed equally to this work. UG participated in the data analyses. DP performed the real-time RT-PCR analyses. DK performed the Affymetrix microarray experiments. RG participated in the design and coordination of the study, including supervision of the bioinformatic analyses. RWK contributed to the design and coordination of the study and participated in the writing of the manuscript. All authors read and approved the final version of the manuscript.

## Supplementary Material

Additional file 1 'Supplementary Table 1A: Genes affected by intra-group, inter-individual mRNA expression variances (RA compared to NC)', 'Supplementary Table 1B: Genes affected by intra-group, inter-individual mRNA expression variances (OA compared to NC)', 'Supplementary Table 1C: Genes affected by intra-group, inter-individual mRNA expression variances (RA compared to OA)'. For KEGG analyses, relevant genes were selected according to (i) a significance level of *p *≤ 0.05 (Bonferroni/Holm corrected Brown-Forsythe version of the Levene test) for variance-fold values and (ii) a cutoff value for absolute variance-fold levels of > 2.5 for higher variances in RA, OA, and NC, respectively. (A) 568 genes were selected for the comparison between RA and NC (307 with higher variances in RA, 261 with higher variances in NC), (B) 542 genes were used for the comparison OA versus NC (314 with higher variances in OA, 228 with higher variances in NC), and (C) 333 genes were selected for the comparison between RA and OA (186 with higher variances in RA, 147 with higher variances in OA). All genes are sorted according to absolute variance-fold values. Click here for file
