# Supplementary material for: Identification of intra-group, inter-individual, and gene-specific variances in mRNA expression profiles in the rheumatoid arthritis synovial membrane
Source: Arthritis Res Ther. 2008 Aug 22;10(4):R98. doi: 10.1186/ar2485 (PMC2575612; doi:10.1186/ar2485)
Supplement: Additional file 1 — 'Supplementary Table 1A: Genes affected by intra-group, inter-individual mRNA expression variances (RA compared to NC)', 'Supplementary Table 1B: Genes affected by intra-group, inter-individual mRNA expression variances (OA compared to NC)', 'Supplementary Table 1C: Genes affected by intra-group, inter-individual mRNA expression variances (RA compared to OA)'. For KEGG analyses, relevant genes were selected according to (i) a significance level of p ≤ 0.05 (Bonferroni/Holm corrected Brown-Forsythe version of the Levene test) for variance-fold values and (ii) a cutoff value for absolute variance-fold levels of > 2.5 for higher variances in RA, OA, and NC, respectively. (A) 568 genes were selected for the comparison between RA and NC (307 with higher variances in RA, 261 with higher variances in NC), (B) 542 genes were used for the comparison OA versus NC (314 with higher variances in OA, 228 with higher variances in NC), and (C) 333 genes were selected for the comparison between RA and OA (186 with higher variances in RA, 147 with higher variances in OA). All genes are sorted according to absolute variance-fold values. [file ar2485-S1.doc]

# Supplementary Table 1A: Genes affected by intra-group, inter-individual mRNA expression variances (RA compared to NC):

**higher variances in RA**

| **GeneSymbol** | **variance Fold** | ***P* Brown-Forsythe** | ***P* Bonferroni** | **Full Annotation** |
| --- | --- | --- | --- | --- |
| PLA2G2D | 2,408444E+002 | 1,000000E-006 | 2,614700E-002 | phospholipase_A2_group_IID |
| IGL@ | 5,650004E+001 | 0,000000E+001 | 7,700000E-005 | Immunoglobulin_lambda_constant_1_(Mcg_marker) |
| N-PAC | 4,963201E+001 | 0,000000E+001 | 3,300000E-005 | cytokine-like_nuclear_factor_n-pac |
| PTPN12 | 3,708733E+001 | 1,000000E-006 | 2,476400E-002 | protein_tyrosine_phosphatase_non-receptor_type_12 |
| SHB | 2,986998E+001 | 0,000000E+001 | 1,500000E-005 | Immunoglobulin_lambda_variable_3-21 |
| AKT2 | 2,822130E+001 | 0,000000E+001 | 3,700000E-005 | v-akt_murine_thymoma_viral_oncogene_homolog_2 |
| ATP2A2 | 2,263330E+001 | 0,000000E+001 | 4,567000E-003 | ATPase_Ca++_transporting_cardiac_muscle_slow_twitch_2 |
| GNRH2 | 2,184672E+001 | 0,000000E+001 | 1,365000E-003 | gonadotropin-releasing_hormone_2 |
| GJB1 | 2,130962E+001 | 0,000000E+001 | 3,210000E-004 | gap_junction_protein_beta_1_32kDa_(connexin_32_Charcot-Marie-Tooth_neuropathy_X-linked) |
| KLK2 | 1,811652E+001 | 0,000000E+001 | 7,200000E-005 | kallikrein_2_prostatic |
| TPD52 | 1,743627E+001 | 0,000000E+001 | 4,900000E-005 | tumor_protein_D52 |
| LAX1 | 1,643868E+001 | 0,000000E+001 | 3,728000E-003 | lymphocyte_transmembrane_adaptor_1 |
| IKBKB | 1,636037E+001 | 0,000000E+001 | 2,120000E-004 | inhibitor_of_kappa_light_polypeptide_gene_enhancer_in_B-cells_kinase_beta |
| TNFRSF17 | 1,583269E+001 | 0,000000E+001 | 4,840000E-004 | tumor_necrosis_factor_receptor_superfamily_member_17 |
| PDK1 | 1,570846E+001 | 0,000000E+001 | 4,000000E-006 | pyruvate_dehydrogenase_kinase_isoenzyme_1 |
| IGHG1 | 1,531929E+001 | 0,000000E+001 | 2,750000E-004 | Immunoglobulin_heavy_constant_gamma_1_(G1m_marker) |
| NDRG2 | 1,529932E+001 | 0,000000E+001 | 1,567000E-003 | NDRG_family_member_2 |
| IFNA8 | 1,480097E+001 | 0,000000E+001 | 3,646000E-003 | interferon_alpha_8 |
| SLC1A4 | 1,464431E+001 | 0,000000E+001 | 1,132000E-003 | solute_carrier_family_1_(glutamate/neutral_amino_acid_transporter)_member_4 |
| SLC26A10 | 1,342841E+001 | 0,000000E+001 | 3,870000E-004 | solute_carrier_family_26_member_10 |
| IL2RG | 1,274599E+001 | 0,000000E+001 | 9,220000E-004 | interleukin_2_receptor_gamma_(severe_combined_immunodeficiency) |
| STX16 | 1,244210E+001 | 0,000000E+001 | 3,800000E-005 | syntaxin_16 |
| IL13 | 1,231768E+001 | 0,000000E+001 | 6,201000E-003 | interleukin_13 |
| ENO3 | 1,200119E+001 | 0,000000E+001 | 2,995000E-003 | enolase_3_(beta_muscle) |
| TNFRSF25 | 1,153798E+001 | 0,000000E+001 | 9,314000E-003 | tumor_necrosis_factor_receptor_superfamily_member_25 |
| SLC12A4 | 1,141440E+001 | 0,000000E+001 | 4,540000E-004 | solute_carrier_family_12_(potassium/chloride_transporters)_member_4 |
| RAB35 | 1,043868E+001 | 0,000000E+001 | 5,000000E-006 | RAB35_member_RAS_oncogene_family |
| PIM2 | 1,028168E+001 | 2,000000E-006 | 4,622400E-002 | pim-2_oncogene |
| EPRS | 9,870952E000 | 0,000000E+001 | 2,220000E-004 | glutamyl-prolyl-tRNA_synthetase |
| MAPK8 | 9,830556E000 | 0,000000E+001 | 5,580000E-004 | mitogen-activated_protein_kinase_8 |
| AD7C-NTP | 9,815662E000 | 0,000000E+001 | 3,990000E-004 | neuronal_thread_protein_AD7c-NTP |
| GTSE1 | 9,742785E000 | 0,000000E+001 | 3,375000E-003 | G-2_and_S-phase_expressed_1 |
| STK10 | 9,670240E000 | 1,000000E-006 | 1,324700E-002 | serine/threonine_kinase_10 |
| DHX9 | 9,635625E000 | 0,000000E+001 | 2,380000E-004 | DEAH_(Asp-Glu-Ala-His)_box_polypeptide_9 |
| IGHM | 9,297771E000 | 0,000000E+001 | 2,692000E-003 | immunoglobulin_heavy_constant_mu |
| PCDHGC3 | 9,239244E000 | 0,000000E+001 | 1,760000E-004 | protocadherin_gamma_subfamily_C_3 |
| MTMR3 | 9,132239E000 | 0,000000E+001 | 3,587000E-003 | myotubularin_related_protein_3 |
| SFRS8 | 9,021760E000 | 0,000000E+001 | 2,000000E-005 | splicing_factor_arginine/serine-rich_8_(suppressor-of-white-apricot_homolog_Drosophila) |
| WIZ | 8,625541E000 | 0,000000E+001 | 3,390000E-004 | widely-interspaced_zinc_finger_motifs |
| KRTAP5-8 | 8,624788E000 | 0,000000E+001 | 1,000000E-006 | keratin_associated_protein_5-8 |
| ADD1 | 8,623693E000 | 0,000000E+001 | 1,340000E-004 | adducin_1_(alpha) |
| MS4A1 | 8,472336E000 | 1,000000E-006 | 2,741100E-002 | membrane-spanning_4-domains_subfamily_A_member_1 |
| CLDN11 | 8,312606E000 | 2,000000E-006 | 4,609500E-002 | claudin_11_(oligodendrocyte_transmembrane_protein) |
| PSMB10 | 8,273333E000 | 0,000000E+001 | 2,082000E-003 | proteasome_(prosome_macropain)_subunit_beta_type_10 |
| GAS8 | 8,272610E000 | 0,000000E+001 | 2,010000E-004 | growth_arrest-specific_8 |
| HLA-C | 8,022072E000 | 0,000000E+001 | 1,310000E-003 | Anti-rabies_virus_immunoglobulin_rearranged_kappa_chain_V-region |
| SDCCAG3 | 7,824300E000 | 0,000000E+001 | 1,005800E-002 | serologically_defined_colon_cancer_antigen_3 |
| ZFP36L2 | 7,748508E000 | 0,000000E+001 | 1,264000E-003 | zinc_finger_protein_36_C3H_type-like_2 |
| SPDEF | 7,641529E000 | 0,000000E+001 | 2,234000E-003 | SAM_pointed_domain_containing_ets_transcription_factor |
| CD27 | 7,472289E000 | 0,000000E+001 | 4,170000E-004 | tumor_necrosis_factor_receptor_superfamily_member_7 |
| PTPRA | 7,468656E000 | 0,000000E+001 | 3,300000E-005 | protein_tyrosine_phosphatase_receptor_type_A |
| PRKACA | 7,457310E000 | 0,000000E+001 | 1,657000E-003 | protein_kinase_cAMP-dependent_catalytic_alpha |
| B4GALT1 | 7,384168E000 | 1,000000E-006 | 1,478800E-002 | UDP-Gal:betaGlcNAc_beta_14-_galactosyltransferase_polypeptide_1 |
| IFNAR2 | 7,291547E000 | 0,000000E+001 | 4,520000E-004 | interferon_(alpha_beta_and_omega)_receptor_2 |
| TGFBR2 | 7,248944E000 | 0,000000E+001 | 1,000000E-006 | transforming_growth_factor_beta_receptor_II_(70/80kDa) |
| ECE2 | 7,134496E000 | 1,000000E-006 | 3,155800E-002 | endothelin_converting_enzyme_2 |
| TRFP | 7,117936E000 | 0,000000E+001 | 3,968000E-003 | Trf_(TATA_binding_protein-related_factor)-proximal_homolog_(Drosophila) |
| SUZ12 | 7,098083E000 | 2,000000E-006 | 3,511900E-002 | suppressor_of_zeste_12_homolog_(Drosophila) |
| CTCF | 7,065926E000 | 0,000000E+001 | 2,327000E-003 | CCCTC-binding_factor_(zinc_finger_protein) |
| EPN1 | 6,985147E000 | 0,000000E+001 | 9,400000E-005 | epsin_1 |
| B2M | 6,695070E000 | 0,000000E+001 | 1,878000E-003 | beta-2-microglobulin |
| GSK3A | 6,663630E000 | 0,000000E+001 | 9,530000E-004 | glycogen_synthase_kinase_3_alpha |
| CDC14B | 6,606903E000 | 0,000000E+001 | 1,417000E-003 | CDC14_cell_division_cycle_14_homolog_B_(S,_cerevisiae) |
| MS4A1 | 6,557774E000 | 0,000000E+001 | 1,092000E-002 | membrane-spanning_4-domains_subfamily_A_member_1 |
| WDR45 | 6,526704E000 | 0,000000E+001 | 5,600000E-005 | WD_repeat_domain_45 |
| FLOT2 | 6,479658E000 | 1,000000E-006 | 2,740700E-002 | flotillin_2 |
| APLP2 | 6,400222E000 | 0,000000E+001 | 4,700000E-005 | Amyloid_beta_(A4)_precursor-like_protein_2 |
| HLA-C | 6,374911E000 | 0,000000E+001 | 5,140000E-004 | immunoglobulin_kappa_variable_1-5 |
| HSF1 | 6,261619E000 | 0,000000E+001 | 1,690000E-004 | heat_shock_transcription_factor_1 |
| SRRM2 | 6,247732E000 | 1,000000E-006 | 2,048600E-002 | Serine/arginine_repetitive_matrix_2 |
| KIAA0746 | 6,106254E000 | 0,000000E+001 | 1,120000E-004 | KIAA0746_protein |
| RPS21 | 6,083627E000 | 0,000000E+001 | 7,286000E-003 | ribosomal_protein_S21 |
| ENG | 6,039006E000 | 0,000000E+001 | 5,000000E-006 | endoglin_(Osler-Rendu-Weber_syndrome_1) |
| DNAJB6 | 5,980996E000 | 0,000000E+001 | 3,216000E-003 | DnaJ_(Hsp40)_homolog_subfamily_B_member_6 |
| NGLY1 | 5,980915E000 | 0,000000E+001 | 4,910000E-004 | N-glycanase_1 |
| PBXIP1 | 5,951743E000 | 2,000000E-006 | 3,902200E-002 | Pre-B-cell_leukemia_transcription_factor_interacting_protein_1 |
| PDCD6 | 5,923234E000 | 0,000000E+001 | 1,071000E-003 | programmed_cell_death_6 |
| CD2 | 5,922647E000 | 2,000000E-006 | 4,787100E-002 | CD2_antigen_(p50)_sheep_red_blood_cell_receptor |
| SFRS5 | 5,881921E000 | 0,000000E+001 | 3,829000E-003 | splicing_factor_arginine/serine-rich_5 |
| HIP1 | 5,863837E000 | 2,000000E-006 | 3,802200E-002 | huntingtin_interacting_protein_1 |
| IGHD | 5,843906E000 | 1,000000E-006 | 3,321000E-002 | immunoglobulin_heavy_constant_delta |
| CUGBP1 | 5,826938E000 | 0,000000E+001 | 1,501000E-003 | CUG_triplet_repeat_RNA_binding_protein_1 |
| SRPR | 5,770418E000 | 0,000000E+001 | 3,000000E-006 | signal_recognition_particle_receptor_('docking_protein') |
| HLA-C | 5,761129E000 | 0,000000E+001 | 3,960000E-004 | similar_to_Ig_kappa_chain |
| PDLIM5 | 5,759303E000 | 0,000000E+001 | 4,000000E-006 | PDZ_and_LIM_domain_5 |
| COL6A1 | 5,711230E000 | 0,000000E+001 | 6,800000E-005 | collagen_type_VI_alpha_1 |
| CREBL1 | 5,626616E000 | 0,000000E+001 | 7,897000E-003 | cAMP_responsive_element_binding_protein-like_1 |
| POU2AF1 | 5,565018E000 | 0,000000E+001 | 8,000000E-005 | POU_domain_class_2_associating_factor_1 |
| NFE2L3 | 5,556085E000 | 0,000000E+001 | 5,461000E-003 | nuclear_factor_(erythroid-derived_2)-like_3 |
| NADK | 5,479623E000 | 0,000000E+001 | 1,164000E-003 | NAD_kinase |
| MFN2 | 5,452690E000 | 0,000000E+001 | 5,900000E-005 | mitofusin_2 |
| FKBP6 | 5,449444E000 | 0,000000E+001 | 9,184000E-003 | FK506_binding_protein_6_36kDa |
| ZDHHC6 | 5,436940E000 | 0,000000E+001 | 1,700000E-004 | zinc_finger_DHHC-type_containing_6 |
| ATXN2L | 5,427459E000 | 2,000000E-006 | 3,963600E-002 | ataxin_2-like |
| PPIA | 5,416168E000 | 0,000000E+001 | 3,000000E-003 | peptidylprolyl_isomerase_A_(cyclophilin_A) |
| ZC3H7B | 5,350527E000 | 1,000000E-006 | 3,206400E-002 | zinc_finger_CCCH-type_containing_7B |
| UNC45A | 5,334675E000 | 0,000000E+001 | 0,000000E+001 | smooth_muscle_cell_associated_protein-1 |
| ARS2 | 5,323709E000 | 0,000000E+001 | 2,900000E-004 | arsenate_resistance_protein_ARS2 |
| RAPGEF1 | 5,257891E000 | 1,000000E-006 | 1,555600E-002 | Rap_guanine_nucleotide_exchange_factor_(GEF)_1 |
| SMARCC1 | 5,250382E000 | 0,000000E+001 | 1,400000E-005 | SWI/SNF_related_matrix_associated_actin_dependent_regulator_of_chromatin_subfamily_c_member_1 |
| ILF3 | 5,248892E000 | 0,000000E+001 | 2,160000E-004 | interleukin_enhancer_binding_factor_3_90kDa |
| IDH3B | 5,209782E000 | 0,000000E+001 | 2,280000E-004 | isocitrate_dehydrogenase_3_(NAD+)_beta |
| GGA3 | 5,149126E000 | 0,000000E+001 | 8,158000E-003 | golgi_associated_gamma_adaptin_ear_containing_ARF_binding_protein_3 |
| TTC3 | 5,118917E000 | 0,000000E+001 | 4,220000E-004 | tetratricopeptide_repeat_domain_3 |
| SMAD2 | 5,109953E000 | 0,000000E+001 | 4,050000E-004 | SMAD_mothers_against_DPP_homolog_2_(Drosophila) |
| ARPC1B | 5,029197E000 | 0,000000E+001 | 8,810000E-004 | Immunoglobulin_heavy_constant_mu |
| KIF25 | 5,023507E000 | 1,000000E-006 | 1,709400E-002 | kinesin_family_member_25 |
| TOP1 | 5,002481E000 | 0,000000E+001 | 7,000000E-006 | topoisomerase_(DNA)_I |
| PHF7 | 4,942621E000 | 1,000000E-006 | 2,390200E-002 | PHD_finger_protein_7 |
| SLAMF1 | 4,884530E000 | 2,000000E-006 | 4,964900E-002 | signaling_lymphocytic_activation_molecule_family_member_1 |
| CXCL13 | 4,853419E000 | 0,000000E+001 | 1,000000E-005 | chemokine_(C-X-C_motif)_ligand_13_(B-cell_chemoattractant) |
| SIT1 | 4,800113E000 | 1,000000E-006 | 2,119200E-002 | signaling_threshold_regulating_transmembrane_adaptor_1 |
| LGALS2 | 4,791920E000 | 0,000000E+001 | 6,429000E-003 | lectin_galactoside-binding_soluble_2_(galectin_2) |
| TRRAP | 4,785024E000 | 0,000000E+001 | 6,000000E-004 | transformation/transcription_domain-associated_protein |
| HLA-C | 4,761916E000 | 0,000000E+001 | 5,680000E-004 | HRV_Fab_027-VL |
| SMEK1 | 4,755452E000 | 1,000000E-006 | 2,486100E-002 | KIAA2010 |
| GSN | 4,713232E000 | 0,000000E+001 | 0,000000E+001 | gelsolin_(amyloidosis_Finnish_type) |
| TRBV19 | 4,686291E000 | 0,000000E+001 | 4,422000E-003 | T_cell_receptor_beta_variable_19 |
| ABI2 | 4,675189E000 | 0,000000E+001 | 6,369000E-003 | abl_interactor_2 |
| MPZL1 | 4,665954E000 | 0,000000E+001 | 3,490000E-004 | myelin_protein_zero-like_1 |
| LSM14A | 4,656355E000 | 0,000000E+001 | 9,320000E-004 | family_with_sequence_similarity_61_member_A |
| RPL14 | 4,633363E000 | 0,000000E+001 | 3,150000E-004 | ribosomal_protein_L14 |
| LTBP4 | 4,632863E000 | 0,000000E+001 | 9,790000E-004 | latent_transforming_growth_factor_beta_binding_protein_4 |
| IL27RA | 4,624938E000 | 0,000000E+001 | 1,296000E-003 | interleukin_27_receptor_alpha |
| GBP1 | 4,576891E000 | 0,000000E+001 | 1,090000E-004 | guanylate_binding_protein_1_interferon-inducible_67kDa |
| CENTB1 | 4,576043E000 | 0,000000E+001 | 1,632000E-003 | centaurin_beta_1 |
| TNPO1 | 4,574755E000 | 0,000000E+001 | 1,156000E-003 | transportin_1 |
| IFI16 | 4,544887E000 | 0,000000E+001 | 1,272000E-003 | interferon_gamma-inducible_protein_16 |
| RBM25 | 4,526747E000 | 0,000000E+001 | 7,750000E-004 | RNA_binding_motif_protein_25 |
| PRODH2 | 4,512688E000 | 0,000000E+001 | 3,400000E-005 | proline_dehydrogenase_(oxidase)_2 |
| PKP1 | 4,504620E000 | 0,000000E+001 | 4,654000E-003 | plakophilin_1_(ectodermal_dysplasia/skin_fragility_syndrome) |
| SETD8 | 4,493178E000 | 1,000000E-006 | 1,857700E-002 | PR/SET_domain_containing_protein_8 |
| MRPL49 | 4,492710E000 | 0,000000E+001 | 2,090000E-004 | mitochondrial_ribosomal_protein_L49 |
| IGL@ | 4,485903E000 | 0,000000E+001 | 5,238000E-003 | Immunoglobulin_lambda_joining_3 |
| RANBP9 | 4,485861E000 | 0,000000E+001 | 3,900000E-005 | RAN_binding_protein_9 |
| APP | 4,471465E000 | 0,000000E+001 | 1,000000E-006 | amyloid_beta_(A4)_precursor_protein_(protease_nexin-II_Alzheimer_disease) |
| MOBK1B | 4,460398E000 | 0,000000E+001 | 1,630000E-004 | MOB1_Mps_One_Binder_kinase_activator-like_1B_(yeast) |
| BICD1 | 4,448266E000 | 0,000000E+001 | 1,060000E-004 | bicaudal_D_homolog_1_(Drosophila) |
| RERE | 4,428342E000 | 1,000000E-006 | 1,892200E-002 | arginine-glutamic_acid_dipeptide_(RE)_repeats |
| TUBGCP2 | 4,405887E000 | 0,000000E+001 | 3,080000E-004 | tubulin_gamma_complex_associated_protein_2 |
| VIL2 | 4,403227E000 | 0,000000E+001 | 5,100000E-005 | villin_2_(ezrin) |
| HSPA4 | 4,390733E000 | 0,000000E+001 | 1,102000E-003 | heat_shock_70kDa_protein_4 |
| NADK | 4,389925E000 | 0,000000E+001 | 3,000000E-006 | NAD_kinase |
| IL8 | 4,372653E000 | 0,000000E+001 | 5,275000E-003 | IgM_VDJ-region |
| KCNG1 | 4,350207E000 | 0,000000E+001 | 7,000000E-006 | potassium_voltage-gated_channel_subfamily_G_member_1 |
| EPAS1 | 4,338465E000 | 0,000000E+001 | 3,140000E-004 | endothelial_PAS_domain_protein_1 |
| POLDIP3 | 4,286040E000 | 0,000000E+001 | 6,810000E-003 | polymerase_(DNA-directed)_delta_interacting_protein_3 |
| PTP4A3 | 4,281605E000 | 1,000000E-006 | 2,155100E-002 | protein_tyrosine_phosphatase_type_IVA_member_3 |
| HBD | 4,277681E000 | 0,000000E+001 | 2,478000E-003 | hemoglobin_delta |
| PYY2 | 4,268385E000 | 2,000000E-006 | 4,884800E-002 | peptide_YY_2_(seminalplasmin) |
| TTF1 | 4,236124E000 | 0,000000E+001 | 2,170000E-004 | transcription_termination_factor_RNA_polymerase_I |
| ZFYVE9 | 4,231081E000 | 1,000000E-006 | 1,926700E-002 | zinc_finger_FYVE_domain_containing_9 |
| TEX261 | 4,211484E000 | 0,000000E+001 | 8,720000E-004 | testis_expressed_sequence_261 |
| CSNK2A1 | 4,210657E000 | 0,000000E+001 | 3,000000E-006 | casein_kinase_2_alpha_1_polypeptide |
| SLC26A1 | 4,210018E000 | 0,000000E+001 | 4,453000E-003 | solute_carrier_family_26_(sulfate_transporter)_member_1 |
| IGLJ3 | 4,190956E000 | 0,000000E+001 | 5,980000E-003 | immunoglobulin_lambda_joining_3 |
| RIN3 | 4,183885E000 | 2,000000E-006 | 4,187000E-002 | Ras_and_Rab_interactor_3 |
| CTA-246H3.1 | 4,154289E000 | 0,000000E+001 | 1,129000E-003 | similar_to_omega_protein |
| BAT1 | 4,137356E000 | 0,000000E+001 | 2,600000E-005 | HLA-B_associated_transcript_1 |
| IRF4 | 4,113445E000 | 0,000000E+001 | 1,498000E-003 | interferon_regulatory_factor_4 |
| CUGBP1 | 4,059037E000 | 0,000000E+001 | 5,250000E-004 | CUG_triplet_repeat_RNA_binding_protein_1 |
| DNAJC7 | 4,055181E000 | 0,000000E+001 | 7,894000E-003 | 2'3'-cyclic_nucleotide_3'_phosphodiesterase |
| N-PAC | 4,053347E000 | 0,000000E+001 | 4,000000E-006 | cytokine-like_nuclear_factor_n-pac |
| IGLJ3 | 4,049789E000 | 0,000000E+001 | 1,070000E-004 | immunoglobulin_lambda_joining_3 |
| NR2F6 | 4,027912E000 | 0,000000E+001 | 9,100000E-005 | nuclear_receptor_subfamily_2_group_F_member_6 |
| TOM1L2 | 4,024050E000 | 0,000000E+001 | 7,770000E-004 | target_of_myb1-like_2_(chicken) |
| THOC2 | 4,023006E000 | 0,000000E+001 | 1,270000E-004 | THO_complex_2 |
| NTN2L | 4,018560E000 | 0,000000E+001 | 4,764000E-003 | IgM_rheumatoid_factor_RF-TT1_variable_heavy_chain |
| SPTBN1 | 4,003393E000 | 0,000000E+001 | 1,100000E-005 | spectrin_beta_non-erythrocytic_1 |
| CTRC | 3,996563E000 | 1,000000E-006 | 1,618600E-002 | chymotrypsin_C_(caldecrin) |
| TRIM46 | 3,992790E000 | 0,000000E+001 | 5,481000E-003 | tripartite_motif-containing_46 |
| PTP4A3 | 3,940080E000 | 0,000000E+001 | 4,960000E-003 | protein_tyrosine_phosphatase_type_IVA_member_3 |
| IGKC | 3,928232E000 | 0,000000E+001 | 1,100000E-004 | HRV_Fab_N8-VL |
| FGF18 | 3,926167E000 | 1,000000E-006 | 2,495300E-002 | Fibroblast_growth_factor_18 |
| ADAMTS7 | 3,904689E000 | 0,000000E+001 | 1,223000E-003 | a_disintegrin-like_and_metalloprotease_(reprolysin_type)_with_thrombospondin_type_1_motif_7 |
| ACBD3 | 3,892451E000 | 0,000000E+001 | 6,482000E-003 | acyl-Coenzyme_A_binding_domain_containing_3 |
| NTRK2 | 3,880474E000 | 0,000000E+001 | 6,832000E-003 | neurotrophic_tyrosine_kinase_receptor_type_2 |
| FSTL3 | 3,867323E000 | 2,000000E-006 | 4,408100E-002 | follistatin-like_3_(secreted_glycoprotein) |
| PAWR | 3,861611E000 | 0,000000E+001 | 7,400000E-005 | PRKC_apoptosis_WT1_regulator |
| RPS10 | 3,852257E000 | 0,000000E+001 | 1,089000E-003 | Ribosomal_protein_S10 |
| RPL35A | 3,817937E000 | 0,000000E+001 | 8,658000E-003 | Ribosomal_protein_L35a |
| CAMKK2 | 3,792826E000 | 0,000000E+001 | 2,520000E-004 | calcium/calmodulin-dependent_protein_kinase_kinase_2_beta |
| CSPP1 | 3,754567E000 | 0,000000E+001 | 1,080000E-004 | centrosome_spindle_pole_associated_protein |
| IL21R | 3,748718E000 | 0,000000E+001 | 1,125000E-003 | interleukin_21_receptor |
| TMEM126B | 3,743442E000 | 1,000000E-006 | 2,461400E-002 | uncharacterized_hypothalamus_protein_HT007 |
| SFI1 | 3,739598E000 | 0,000000E+001 | 1,950000E-004 | Sfi1_homolog_spindle_assembly_associated_(yeast) |
| MATN4 | 3,738818E000 | 2,000000E-006 | 4,698200E-002 | matrilin_4 |
| EXTL3 | 3,737766E000 | 0,000000E+001 | 6,150000E-004 | exostoses_(multiple)-like_3 |
| BCL2L1 | 3,733861E000 | 0,000000E+001 | 2,905000E-003 | BCL2-like_1 |
| CDC2L1 | 3,723305E000 | 0,000000E+001 | 1,900000E-004 | cell_division_cycle_2-like_1_(PITSLRE_proteins) |
| MAP4 | 3,722586E000 | 0,000000E+001 | 2,872000E-003 | microtubule-associated_protein_4 |
| GTF3C2 | 3,671640E000 | 0,000000E+001 | 1,849000E-003 | general_transcription_factor_IIIC_polypeptide_2_beta_110kDa |
| IHPK1 | 3,639211E000 | 1,000000E-006 | 2,692300E-002 | inositol_hexaphosphate_kinase_1 |
| IRF5 | 3,635041E000 | 0,000000E+001 | 3,277000E-003 | interferon_regulatory_factor_5 |
| FUT2 | 3,631484E000 | 0,000000E+001 | 3,570000E-004 | fucosyltransferase_2_(secretor_status_included) |
| TAF6L | 3,627747E000 | 0,000000E+001 | 4,229000E-003 | TAF6-like_RNA_polymerase_II_p300/CBP-associated_factor_(PCAF)-associated_factor_65kDa |
| CALR | 3,627322E000 | 0,000000E+001 | 1,142000E-003 | Calreticulin |
| TOB2 | 3,611560E000 | 2,000000E-006 | 4,524200E-002 | transducer_of_ERBB2_2 |
| ANAPC5 | 3,605366E000 | 0,000000E+001 | 2,100000E-005 | anaphase_promoting_complex_subunit_5 |
| B2M | 3,575276E000 | 0,000000E+001 | 4,000000E-006 | beta-2-microglobulin |
| CCPG1 | 3,571680E000 | 0,000000E+001 | 2,219000E-003 | cell_cycle_progression_1 |
| XPO7 | 3,556348E000 | 0,000000E+001 | 2,300000E-005 | exportin_7 |
| MRP63 | 3,553395E000 | 0,000000E+001 | 1,400000E-005 | mitochondrial_ribosomal_protein_63 |
| KCNQ2 | 3,533920E000 | 0,000000E+001 | 2,000000E-005 | potassium_voltage-gated_channel_KQT-like_subfamily_member_2 |
| MCL1 | 3,520230E000 | 0,000000E+001 | 4,600000E-005 | myeloid_cell_leukemia_sequence_1_(BCL2-related) |
| CACNB1 | 3,500174E000 | 1,000000E-006 | 2,142700E-002 | calcium_channel_voltage-dependent_beta_1_subunit |
| RBM9 | 3,472016E000 | 1,000000E-006 | 1,666300E-002 | RNA_binding_motif_protein_9 |
| WDR1 | 3,436313E000 | 0,000000E+001 | 6,676000E-003 | WD_repeat_domain_1 |
| DLST | 3,426204E000 | 1,000000E-006 | 1,620800E-002 | dihydrolipoamide_S-succinyltransferase_(E2_component_of_2-oxo-glutarate_complex) |
| PCDH1 | 3,419741E000 | 1,000000E-006 | 1,598300E-002 | protocadherin_1_(cadherin-like_1) |
| CIAO1 | 3,379031E000 | 0,000000E+001 | 2,500000E-005 | WD_repeat_domain_39 |
| FRMD4A | 3,351263E000 | 0,000000E+001 | 8,346000E-003 | FERM_domain_containing_4A |
| DNAJB6 | 3,330771E000 | 0,000000E+001 | 3,800000E-004 | DnaJ_(Hsp40)_homolog_subfamily_B_member_6 |
| PBX1 | 3,314258E000 | 0,000000E+001 | 1,090000E-004 | Pre-B-cell_leukemia_transcription_factor_1 |
| AFTPH | 3,301784E000 | 0,000000E+001 | 3,400000E-005 | aftiphilin_protein |
| PKP4 | 3,287313E000 | 0,000000E+001 | 6,000000E-006 | plakophilin_4 |
| SPCS2 | 3,272168E000 | 0,000000E+001 | 9,186000E-003 | signal_peptidase_complex_subunit_2_homolog_(S,_cerevisiae) |
| ACVR1B | 3,272023E000 | 1,000000E-006 | 1,736200E-002 | activin_A_receptor_type_IB |
| MCFD2 | 3,254456E000 | 0,000000E+001 | 9,010000E-004 | multiple_coagulation_factor_deficiency_2 |
| PLK3 | 3,241026E000 | 2,000000E-006 | 3,629900E-002 | polo-like_kinase_3_(Drosophila) |
| CXCL10 | 3,240305E000 | 0,000000E+001 | 1,000000E-005 | chemokine_(C-X-C_motif)_ligand_10 |
| CHP | 3,233993E000 | 0,000000E+001 | 5,410000E-003 | calcium_binding_protein_P22 |
| PRSS1 | 3,221935E000 | 0,000000E+001 | 1,280000E-004 | T-cell_receptor_beta_chain_V3-D-J2,7-C2_region |
| IGKC | 3,217631E000 | 1,000000E-006 | 1,575100E-002 | immunoglobulin_kappa_constant |
| IGL@ | 3,182497E000 | 0,000000E+001 | 7,030000E-004 | Immunoglobulin_lambda_variable_3-21 |
| HADHA | 3,175746E000 | 0,000000E+001 | 8,000000E-006 | hydroxyacyl-Coenzyme_A_dehydrogenase/3-ketoacyl-Coenzyme_A_thiolase/enoyl-Coenzyme_A_hydratase |
| ADAMDEC1 | 3,172997E000 | 0,000000E+001 | 1,400000E-005 | ADAM-like_decysin_1 |
| KIAA0251 | 3,167545E000 | 1,000000E-006 | 2,189900E-002 | Similar_to_Group_X_secretory_phospholipase_A2_precursor_(Phosphatidylcholine_2-acylhydrolase_GX) |
| CROP | 3,158945E000 | 0,000000E+001 | 9,130000E-004 | cisplatin_resistance-associated_overexpressed_protein |
| CDH1 | 3,139618E000 | 0,000000E+001 | 3,950000E-004 | cadherin_1_type_1_E-cadherin_(epithelial) |
| ARL1 | 3,135648E000 | 0,000000E+001 | 2,900000E-005 | ADP-ribosylation_factor-like_1 |
| IFI6 | 3,134704E000 | 0,000000E+001 | 2,009000E-003 | immunoglobulin_heavy_constant_alpha_1 |
| HIPK1 | 3,132424E000 | 2,000000E-006 | 4,760800E-002 | homeodomain_interacting_protein_kinase_1 |
| MEG3 | 3,128115E000 | 0,000000E+001 | 2,900000E-005 | maternally_expressed_3 |
| DAPK3 | 3,126543E000 | 0,000000E+001 | 1,700000E-005 | death-associated_protein_kinase_3 |
| BET1L | 3,124078E000 | 2,000000E-006 | 4,220500E-002 | blocked_early_in_transport_1_homolog_(S,_cerevisiae)_like |
| TXNIP | 3,119571E000 | 0,000000E+001 | 4,370000E-004 | thioredoxin_interacting_protein |
| IL4 | 3,111632E000 | 0,000000E+001 | 2,701000E-003 | interleukin_4 |
| CHKA | 3,098052E000 | 0,000000E+001 | 6,528000E-003 | choline_kinase_alpha |
| ELL2 | 3,071529E000 | 1,000000E-006 | 2,715400E-002 | elongation_factor_RNA_polymerase_II_2 |
| HTATSF1 | 3,060877E000 | 0,000000E+001 | 6,700000E-005 | HIV_TAT_specific_factor_1 |
| SF3B1 | 3,053530E000 | 0,000000E+001 | 7,890000E-004 | splicing_factor_3b_subunit_1_155kDa |
| FOXJ3 | 3,051796E000 | 0,000000E+001 | 5,000000E-006 | forkhead_box_J3 |
| PRKX | 3,047492E000 | 0,000000E+001 | 1,159000E-003 | protein_kinase_X-linked |
| NUDT7 | 3,033430E000 | 0,000000E+001 | 8,415000E-003 | nudix_(nucleoside_diphosphate_linked_moiety_X)-type_motif_7 |
| E2F3 | 3,023623E000 | 0,000000E+001 | 3,500000E-005 | E2F_transcription_factor_3 |
| ATP8A2 | 3,013026E000 | 0,000000E+001 | 8,460000E-004 | ATPase_aminophospholipid_transporter-like_Class_I_type_8A_member_2 |
| MTMR10 | 2,998041E000 | 2,000000E-006 | 4,697700E-002 | myotubularin_related_protein_10 |
| APP | 2,996492E000 | 0,000000E+001 | 5,706000E-003 | amyloid_beta_(A4)_precursor_protein_(protease_nexin-II_Alzheimer_disease) |
| PSMB9 | 2,989445E000 | 0,000000E+001 | 2,000000E-006 | proteasome_(prosome_macropain)_subunit_beta_type_9_(large_multifunctional_protease_2) |
| TRBV19 | 2,977944E000 | 0,000000E+001 | 1,363000E-003 | T_cell_receptor_beta_variable_19 |
| FBXW12 | 2,971594E000 | 0,000000E+001 | 2,409000E-003 | F-box_and_WD-40_domain_protein_12 |
| PSCD4 | 2,949333E000 | 1,000000E-006 | 1,810100E-002 | pleckstrin_homology_Sec7_and_coiled-coil_domains_4 |
| TPD52 | 2,947980E000 | 0,000000E+001 | 3,700000E-005 | tumor_protein_D52 |
| LRRC42 | 2,947522E000 | 0,000000E+001 | 1,106000E-003 | leucine_rich_repeat_containing_42 |
| MCF2L | 2,933359E000 | 1,000000E-006 | 1,179100E-002 | MCF,2_cell_line_derived_transforming_sequence-like |
| CD79A | 2,931222E000 | 0,000000E+001 | 1,459000E-003 | CD79A_antigen_(immunoglobulin-associated_alpha) |
| SDK1 | 2,919593E000 | 0,000000E+001 | 4,487000E-003 | Sidekick_homolog_1_(chicken) |
| PCDHGA3 | 2,918217E000 | 0,000000E+001 | 3,200000E-005 | protocadherin_gamma_subfamily_A_3 |
| IGKV1D-13 | 2,907736E000 | 0,000000E+001 | 3,600000E-005 | immunoglobulin_kappa_variable_1D-13 |
| DOCK6 | 2,891999E000 | 1,000000E-006 | 2,267000E-002 | dedicator_of_cytokinesis_6 |
| CHD4 | 2,884485E000 | 1,000000E-006 | 1,903600E-002 | chromodomain_helicase_DNA_binding_protein_4 |
| HNRPU | 2,862927E000 | 1,000000E-006 | 1,695700E-002 | heterogeneous_nuclear_ribonucleoprotein_U_(scaffold_attachment_factor_A) |
| STAT1 | 2,860490E000 | 0,000000E+001 | 1,412000E-003 | signal_transducer_and_activator_of_transcription_1_91kDa |
| PHF11 | 2,853911E000 | 0,000000E+001 | 4,930000E-004 | PHD_finger_protein_11 |
| CRYM | 2,838813E000 | 2,000000E-006 | 4,335000E-002 | crystallin_mu |
| PISD | 2,829739E000 | 0,000000E+001 | 2,810000E-004 | phosphatidylserine_decarboxylase |
| PAX9 | 2,827578E000 | 0,000000E+001 | 8,444000E-003 | paired_box_gene_9 |
| SLC9A5 | 2,826907E000 | 0,000000E+001 | 1,344000E-003 | solute_carrier_family_9_(sodium/hydrogen_exchanger)_isoform_5 |
| PRDM2 | 2,799279E000 | 0,000000E+001 | 2,107000E-003 | PR_domain_containing_2_with_ZNF_domain |
| TPP1 | 2,787833E000 | 0,000000E+001 | 3,140000E-003 | tripeptidyl_peptidase_I |
| BACH2 | 2,768706E000 | 1,000000E-006 | 2,435100E-002 | BTB_and_CNC_homology_1_basic_leucine_zipper_transcription_factor_2 |
| TM9SF4 | 2,748786E000 | 1,000000E-006 | 2,864800E-002 | transmembrane_9_superfamily_protein_member_4 |
| COPA | 2,743247E000 | 0,000000E+001 | 1,374000E-003 | coatomer_protein_complex_subunit_alpha |
| TOX | 2,740406E000 | 0,000000E+001 | 6,638000E-003 | thymus_high_mobility_group_box_protein_TOX |
| RHOA | 2,734009E000 | 0,000000E+001 | 4,928000E-003 | ras_homolog_gene_family_member_A |
| PLCG1 | 2,724935E000 | 0,000000E+001 | 9,666000E-003 | phospholipase_C_gamma_1 |
| ZBTB17 | 2,714128E000 | 0,000000E+001 | 7,411000E-003 | zinc_finger_and_BTB_domain_containing_17 |
| WDFY3 | 2,710289E000 | 1,000000E-006 | 2,532900E-002 | WD_repeat_and_FYVE_domain_containing_3 |
| MAP3K7IP2 | 2,708943E000 | 0,000000E+001 | 7,052000E-003 | mitogen-activated_protein_kinase_kinase_kinase_7_interacting_protein_2 |
| HDHD1A | 2,705605E000 | 0,000000E+001 | 8,500000E-004 | haloacid_dehalogenase-like_hydrolase_domain_containing_1A |
| PARP1 | 2,686326E000 | 0,000000E+001 | 2,820000E-003 | poly_(ADP-ribose)_polymerase_family_member_1 |
| NFKB2 | 2,683123E000 | 0,000000E+001 | 9,220000E-004 | nuclear_factor_of_kappa_light_polypeptide_gene_enhancer_in_B-cells_2_(p49/p100) |
| SKAP1 | 2,674935E000 | 1,000000E-006 | 1,321600E-002 | src_family_associated_phosphoprotein_1 |
| PLEC1 | 2,658309E000 | 0,000000E+001 | 6,470000E-004 | plectin_1_intermediate_filament_binding_protein_500kDa |
| ARHGAP1 | 2,636522E000 | 0,000000E+001 | 2,300000E-005 | Rho_GTPase_activating_protein_1 |
| EIF4G2 | 2,636047E000 | 0,000000E+001 | 3,900000E-005 | eukaryotic_translation_initiation_factor_4_gamma_2 |
| EEF1D | 2,630123E000 | 0,000000E+001 | 5,000000E-005 | Eukaryotic_translation_elongation_factor_1_delta_(guanine_nucleotide_exchange_protein) |
| SET | 2,592771E000 | 0,000000E+001 | 4,598000E-003 | SET_translocation_(myeloid_leukemia-associated) |
| SF3B1 | 2,591308E000 | 1,000000E-006 | 2,207100E-002 | splicing_factor_3b_subunit_1_155kDa |
| PTPRU | 2,575519E000 | 1,000000E-006 | 1,767200E-002 | protein_tyrosine_phosphatase_receptor_type_U |
| BAT2D1 | 2,571812E000 | 0,000000E+001 | 7,620000E-004 | BAT2_domain_containing_1 |
| KRT33A | 2,571680E000 | 2,000000E-006 | 3,528600E-002 | keratin_hair_acidic_3A |
| ECT2 | 2,571380E000 | 0,000000E+001 | 3,491000E-003 | epithelial_cell_transforming_sequence_2_oncogene |
| TNKS | 2,562568E000 | 1,000000E-006 | 2,614900E-002 | tankyrase_TRF1-interacting_ankyrin-related_ADP-ribose_polymerase |
| RGS12 | 2,560512E000 | 0,000000E+001 | 5,010000E-004 | regulator_of_G-protein_signalling_12 |
| EDA | 2,551415E000 | 1,000000E-006 | 2,350600E-002 | ectodysplasin_A |
| JAK2 | 2,550451E000 | 0,000000E+001 | 4,200000E-005 | Janus_kinase_2_(a_protein_tyrosine_kinase) |
| SLC7A8 | 2,541329E000 | 0,000000E+001 | 1,000000E-006 | solute_carrier_family_7_(cationic_amino_acid_transporter_y+_system)_member_8 |
| IGL@ | 2,531699E000 | 0,000000E+001 | 4,850000E-004 | Clone_bsmneg3-t5_nonfunctional_immunoglobulin_light_chain_(IGL)_mRNA_partial_sequence |
| INPP4A | 2,527360E000 | 0,000000E+001 | 1,638000E-003 | inositol_polyphosphate-4-phosphatase_type_I_107kDa |
| ASH1L | 2,522850E000 | 0,000000E+001 | 6,080000E-003 | ash1_(absent_small_or_homeotic)-like_(Drosophila) |
| NR5A2 | 2,522729E000 | 0,000000E+001 | 1,159000E-003 | nuclear_receptor_subfamily_5_group_A_member_2 |
| ATP2A3 | 2,519107E000 | 0,000000E+001 | 3,400000E-005 | ATPase_Ca++_transporting_ubiquitous |
| COX17 | 2,517013E000 | 1,000000E-006 | 3,225700E-002 | COX17_homolog_cytochrome_c_oxidase_assembly_protein_(yeast) |
| RHOBTB3 | 2,507792E000 | 0,000000E+001 | 1,886000E-003 | Rho-related_BTB_domain_containing_3 |
| NTN2L | 2,502805E000 | 0,000000E+001 | 7,914000E-003 | IgM_rheumatoid_factor_RF-TT1_variable_heavy_chain |

**higher variances in NC**

| **GeneSymbol** | **variance Fold** | ***P* Brown-Forsythe** | ***P* Bonferroni** | **Full Annotation** |
| --- | --- | --- | --- | --- |
| TFAM | 2,513357E-000 | 0,000000E+001 | 7,000000E-006 | transcription_factor_A_mitochondrial |
| MEOX1 | 2,520999E-000 | 2,000000E-006 | 4,988400E-002 | mesenchyme_homeo_box_1 |
| CYB561 | 2,523809E-000 | 0,000000E+001 | 1,080000E-004 | cytochrome_b-561 |
| JUND | 2,524257E-000 | 0,000000E+001 | 6,590000E-004 | jun_D_proto-oncogene |
| CCDC47 | 2,537211E-000 | 0,000000E+001 | 1,990000E-003 | GK001_protein |
| JAK2 | 2,537500E-000 | 2,000000E-006 | 4,285700E-002 | Janus_kinase_2_(a_protein_tyrosine_kinase) |
| CES4 | 2,541276E-000 | 0,000000E+001 | 3,000000E-005 | carboxylesterase_4-like |
| SNX6 | 2,541738E-000 | 0,000000E+001 | 2,094000E-003 | sorting_nexin_6 |
| NMUR1 | 2,553035E-000 | 0,000000E+001 | 3,874000E-003 | neuromedin_U_receptor_1 |
| HES1 | 2,561834E-000 | 0,000000E+001 | 2,000000E-006 | hairy_and_enhancer_of_split_1_(Drosophila) |
| LEPROTL1 | 2,564169E-000 | 0,000000E+001 | 1,400000E-005 | leptin_receptor_overlapping_transcript-like_1 |
| TM9SF1 | 2,564384E-000 | 0,000000E+001 | 7,433000E-003 | transmembrane_9_superfamily_member_1 |
| TGDS | 2,566223E-000 | 0,000000E+001 | 2,126000E-003 | TDP-glucose_46-dehydratase |
| ADARB1 | 2,571445E-000 | 1,000000E-006 | 2,910800E-002 | adenosine_deaminase_RNA-specific_B1_(RED1_homolog_rat) |
| RPL3 | 2,574279E-000 | 0,000000E+001 | 9,170000E-004 | ribosomal_protein_L3 |
| BAT2 | 2,582055E-000 | 0,000000E+001 | 1,916000E-003 | HLA-B_associated_transcript_2 |
| LDLR | 2,586703E-000 | 0,000000E+001 | 3,588000E-003 | low_density_lipoprotein_receptor_(familial_hypercholesterolemia) |
| EIF1 | 2,598712E-000 | 0,000000E+001 | 1,000000E-006 | putative_translation_initiation_factor |
| LTA4H | 2,600436E-000 | 0,000000E+001 | 9,286000E-003 | leukotriene_A4_hydrolase |
| GPX3 | 2,602735E-000 | 0,000000E+001 | 1,816000E-003 | glutathione_peroxidase_3_(plasma) |
| RPL30 | 2,613418E-000 | 0,000000E+001 | 2,300000E-005 | ribosomal_protein_L30 |
| RPL7 | 2,620431E-000 | 0,000000E+001 | 9,894000E-003 | ribosomal_protein_L7 |
| PLEKHA1 | 2,623453E-000 | 0,000000E+001 | 1,154000E-003 | pleckstrin_homology_domain_containing_family_A_(phosphoinositide_binding_specific)_member_1 |
| CSF1 | 2,639172E-000 | 0,000000E+001 | 9,400000E-005 | colony_stimulating_factor_1_(macrophage) |
| RPL21 | 2,639663E-000 | 0,000000E+001 | 6,681000E-003 | ribosomal_protein_L21 |
| RPL7 | 2,656086E-000 | 0,000000E+001 | 7,500000E-005 | ribosomal_protein_L7 |
| HERPUD1 | 2,656560E-000 | 1,000000E-006 | 3,134600E-002 | homocysteine-inducible_endoplasmic_reticulum_stress-inducible_ubiquitin-like_domain_member_1 |
| NR4A1 | 2,657800E-000 | 0,000000E+001 | 1,092000E-003 | nuclear_receptor_subfamily_4_group_A_member_1 |
| RPL7A | 2,666393E-000 | 0,000000E+001 | 3,630000E-004 | ribosomal_protein_L7a |
| GOLGA5 | 2,667691E-000 | 0,000000E+001 | 1,130000E-004 | golgi_autoantigen_golgin_subfamily_a_5 |
| FOXO3A | 2,695715E-000 | 0,000000E+001 | 4,000000E-006 | forkhead_box_O3A |
| LRP1 | 2,695879E-000 | 1,000000E-006 | 1,255500E-002 | low_density_lipoprotein-related_protein_1_(alpha-2-macroglobulin_receptor) |
| EML2 | 2,704238E-000 | 2,000000E-006 | 4,212400E-002 | echinoderm_microtubule_associated_protein_like_2 |
| VDAC1 | 2,711997E-000 | 1,000000E-006 | 1,905800E-002 | voltage-dependent_anion_channel_1 |
| ANXA9 | 2,735862E-000 | 0,000000E+001 | 6,500000E-005 | annexin_A9 |
| HTRA1 | 2,735950E-000 | 1,000000E-006 | 1,121200E-002 | protease_serine_11_(IGF_binding) |
| ENTPD1 | 2,740155E-000 | 0,000000E+001 | 1,983000E-003 | ectonucleoside_triphosphate_diphosphohydrolase_1 |
| MAP7 | 2,743798E-000 | 0,000000E+001 | 1,200000E-005 | microtubule-associated_protein_7 |
| VIL2 | 2,761307E-000 | 0,000000E+001 | 4,991000E-003 | villin_2_(ezrin) |
| IL1RL2 | 2,772647E-000 | 0,000000E+001 | 2,900000E-005 | interleukin_1_receptor-like_2 |
| EGR1 | 2,783850E-000 | 0,000000E+001 | 2,600000E-005 | early_growth_response_1 |
| DNAJB9 | 2,785903E-000 | 0,000000E+001 | 1,100000E-005 | DnaJ_(Hsp40)_homolog_subfamily_B_member_9 |
| RPS17 | 2,787416E-000 | 0,000000E+001 | 1,098000E-003 | ribosomal_protein_S17 |
| MANEA | 2,800601E-000 | 0,000000E+001 | 2,074000E-003 | mannosidase_endo-alpha |
| MYBL1 | 2,812280E-000 | 0,000000E+001 | 1,461000E-003 | v-myb_myeloblastosis_viral_oncogene_homolog_(avian)-like_1 |
| TNFSF10 | 2,831725E-000 | 0,000000E+001 | 3,160000E-004 | tumor_necrosis_factor_(ligand)_superfamily_member_10 |
| CACYBP | 2,834684E-000 | 1,000000E-006 | 1,213600E-002 | calcyclin_binding_protein |
| TRA@ | 2,857105E-000 | 0,000000E+001 | 1,198000E-003 | Vd7_gene_for_T-cell_receptor_V-delta-7 |
| CDC2 | 2,872259E-000 | 2,000000E-006 | 3,428100E-002 | Cell_division_cycle_2_G1_to_S_and_G2_to_M |
| LDB3 | 2,891967E-000 | 0,000000E+001 | 1,013000E-003 | LIM_domain_binding_3 |
| PBX2 | 2,897284E-000 | 0,000000E+001 | 1,390000E-003 | pre-B-cell_leukemia_transcription_factor_2 |
| NUFIP1 | 2,898370E-000 | 2,000000E-006 | 4,743700E-002 | nuclear_fragile_X_mental_retardation_protein_interacting_protein_1 |
| ENTPD1 | 2,901758E-000 | 1,000000E-006 | 1,366600E-002 | ectonucleoside_triphosphate_diphosphohydrolase_1 |
| PSEN1 | 2,901955E-000 | 0,000000E+001 | 5,000000E-006 | presenilin_1_(Alzheimer_disease_3) |
| PNMA2 | 2,914037E-000 | 1,000000E-006 | 1,668400E-002 | paraneoplastic_antigen_MA2 |
| LETM1 | 2,921461E-000 | 2,000000E-006 | 4,693200E-002 | Fibroblast_growth_factor_receptor_3_(achondroplasia_thanatophoric_dwarfism) |
| SGPP1 | 2,932850E-000 | 0,000000E+001 | 1,068400E-002 | sphingosine-1-phosphate_phosphatase_1 |
| LRRC15 | 2,938066E-000 | 0,000000E+001 | 1,110000E-004 | leucine_rich_repeat_containing_15 |
| SNED1 | 2,947128E-000 | 1,000000E-006 | 2,810200E-002 | sushi_nidogen_and_EGF-like_domains_1 |
| KCNK7 | 2,950454E-000 | 0,000000E+001 | 4,600000E-005 | potassium_channel_subfamily_K_member_7 |
| DCK | 2,963532E-000 | 0,000000E+001 | 1,661000E-003 | deoxycytidine_kinase |
| ATP8B1 | 2,974464E-000 | 0,000000E+001 | 5,174000E-003 | ATPase_Class_I_type_8B_member_1 |
| ZFYVE26 | 2,974754E-000 | 1,000000E-006 | 3,335400E-002 | zinc_finger_FYVE_domain_containing_26 |
| SYT11 | 2,980182E-000 | 0,000000E+001 | 2,440000E-004 | synaptotagmin_XI |
| RPS12 | 2,981193E-000 | 0,000000E+001 | 1,500000E-005 | ribosomal_protein_S12 |
| SARM1 | 2,983012E-000 | 0,000000E+001 | 1,216000E-003 | sterile_alpha_and_TIR_motif_containing_1 |
| SEC23A | 2,989649E-000 | 2,000000E-006 | 3,794300E-002 | Sec23_homolog_A_(S,_cerevisiae) |
| CACNG5 | 2,991438E-000 | 1,000000E-006 | 2,289100E-002 | calcium_channel_voltage-dependent_gamma_subunit_5 |
| NUMA1 | 2,992826E-000 | 2,000000E-006 | 3,809100E-002 | nuclear_mitotic_apparatus_protein_1 |
| TMF1 | 2,994077E-000 | 1,000000E-006 | 2,654800E-002 | TATA_element_modulatory_factor_1 |
| PRKACB | 2,996363E-000 | 0,000000E+001 | 1,400000E-005 | protein_kinase_cAMP-dependent_catalytic_beta |
| HNRPA0 | 2,998425E-000 | 0,000000E+001 | 8,200000E-005 | heterogeneous_nuclear_ribonucleoprotein_A0 |
| EIF5A2 | 3,008925E-000 | 1,000000E-006 | 2,776900E-002 | eukaryotic_translation_initiation_factor_5A2 |
| PTTG1IP | 3,009553E-000 | 0,000000E+001 | 3,452000E-003 | pituitary_tumor-transforming_1_interacting_protein |
| INSL6 | 3,024487E-000 | 0,000000E+001 | 2,620000E-004 | insulin-like_6 |
| CYR61 | 3,033676E-000 | 1,000000E-006 | 1,673300E-002 | cysteine-rich_angiogenic_inducer_61 |
| FLOT1 | 3,037290E-000 | 0,000000E+001 | 8,900000E-005 | flotillin_1 |
| PFDN6 | 3,049602E-000 | 0,000000E+001 | 1,560000E-004 | HLA_class_II_region_expressed_gene_KE2 |
| STAT1 | 3,068225E-000 | 1,000000E-006 | 2,668400E-002 | signal_transducer_and_activator_of_transcription_1_91kDa |
| ATG4A | 3,085927E-000 | 0,000000E+001 | 2,345000E-003 | APG4_autophagy_4_homolog_A_(S,_cerevisiae) |
| GIMAP5 | 3,094729E-000 | 0,000000E+001 | 9,900000E-005 | GTPase_IMAP_family_member_5 |
| LAP3 | 3,161948E-000 | 0,000000E+001 | 7,750000E-004 | leucine_aminopeptidase_3 |
| NCOR1 | 3,167284E-000 | 0,000000E+001 | 2,240000E-004 | nuclear_receptor_co-repressor_1 |
| RABAC1 | 3,174770E-000 | 0,000000E+001 | 3,540000E-004 | Rab_acceptor_1_(prenylated) |
| DPP8 | 3,183329E-000 | 0,000000E+001 | 1,700000E-004 | dipeptidylpeptidase_8 |
| GAP43 | 3,183380E-000 | 2,000000E-006 | 4,141300E-002 | growth_associated_protein_43 |
| SUMO3 | 3,184778E-000 | 0,000000E+001 | 5,016000E-003 | SMT3_suppressor_of_mif_two_3_homolog_3_(yeast) |
| SRD5A1 | 3,202663E-000 | 0,000000E+001 | 3,260000E-004 | steroid-5-alpha-reductase_alpha_polypeptide_1_(3-oxo-5_alpha-steroid_delta_4-dehydrogenase_alpha_1) |
| FUT8 | 3,203710E-000 | 0,000000E+001 | 7,950000E-004 | fucosyltransferase_8_(alpha_(16)_fucosyltransferase) |
| NLRP2 | 3,204224E-000 | 0,000000E+001 | 3,284000E-003 | NACHT_leucine_rich_repeat_and_PYD_containing_2 |
| OPTN | 3,208704E-000 | 0,000000E+001 | 2,230000E-004 | optineurin |
| HMMR | 3,209813E-000 | 0,000000E+001 | 8,650000E-004 | hyaluronan-mediated_motility_receptor_(RHAMM) |
| LILRA2 | 3,215670E-000 | 0,000000E+001 | 3,000000E-005 | leukocyte_immunoglobulin-like_receptor_subfamily_A_(with_TM_domain)_member_2 |
| IGFBP5 | 3,234919E-000 | 0,000000E+001 | 8,362000E-003 | insulin-like_growth_factor_binding_protein_5 |
| RPL9 | 3,246164E-000 | 0,000000E+001 | 8,000000E-006 | ribosomal_protein_L9 |
| SELI | 3,258584E-000 | 0,000000E+001 | 1,030000E-004 | ribosomal_protein_S3A |
| ZNF330 | 3,259241E-000 | 0,000000E+001 | 1,391000E-003 | zinc_finger_protein_330 |
| SOCS3 | 3,285295E-000 | 0,000000E+001 | 1,750000E-003 | suppressor_of_cytokine_signaling_3 |
| ZBTB7C | 3,296328E-000 | 0,000000E+001 | 2,000000E-006 | BTB/POZ-zinc_finger_protein-like |
| ZFP36 | 3,317417E-000 | 0,000000E+001 | 2,627000E-003 | zinc_finger_protein_36_C3H_type_homolog_(mouse) |
| RPL23 | 3,335908E-000 | 0,000000E+001 | 2,900000E-005 | ribosomal_protein_L23 |
| PRKACB | 3,357358E-000 | 0,000000E+001 | 1,385000E-003 | protein_kinase_cAMP-dependent_catalytic_beta |
| PLAA | 3,362743E-000 | 1,000000E-006 | 2,659200E-002 | phospholipase_A2-activating_protein |
| TRIM3 | 3,370773E-000 | 0,000000E+001 | 1,800000E-004 | tripartite_motif-containing_3 |
| RPS18 | 3,378039E-000 | 0,000000E+001 | 1,020000E-004 | ribosomal_protein_S18 |
| RPS10 | 3,461375E-000 | 0,000000E+001 | 3,434000E-003 | ribosomal_protein_S10 |
| RPS6 | 3,472262E-000 | 0,000000E+001 | 2,000000E-006 | ribosomal_protein_S6 |
| RAB6A | 3,480084E-000 | 0,000000E+001 | 2,700000E-005 | RAB6A_member_RAS_oncogene_family |
| AK2 | 3,496131E-000 | 0,000000E+001 | 8,020000E-004 | adenylate_kinase_2 |
| FLOT1 | 3,518748E-000 | 2,000000E-006 | 4,626400E-002 | flotillin_1 |
| AGT | 3,541045E-000 | 0,000000E+001 | 5,797000E-003 | angiotensinogen_(serine_(or_cysteine)_proteinase_inhibitor_clade_A |
| NR4A3 | 3,563393E-000 | 0,000000E+001 | 7,490000E-004 | nuclear_receptor_subfamily_4_group_A_member_3 |
| PREP | 3,569458E-000 | 1,000000E-006 | 3,307200E-002 | prolyl_endopeptidase |
| AIM2 | 3,577921E-000 | 0,000000E+001 | 1,340000E-004 | absent_in_melanoma_2 |
| CREBL2 | 3,590147E-000 | 0,000000E+001 | 3,162000E-003 | cAMP_responsive_element_binding_protein-like_2 |
| SAMD9 | 3,612636E-000 | 1,000000E-006 | 1,565300E-002 | sterile_alpha_motif_domain_containing_9 |
| ATP6V1C1 | 3,650648E-000 | 0,000000E+001 | 1,065400E-002 | ATPase_H+_transporting_lysosomal_42kDa_V1_subunit_C_isoform_1 |
| SPINT1 | 3,655479E-000 | 0,000000E+001 | 2,283000E-003 | serine_protease_inhibitor_Kunitz_type_1 |
| KIAA0274 | 3,701543E-000 | 1,000000E-006 | 2,303600E-002 | KIAA0274 |
| RND1 | 3,705283E-000 | 0,000000E+001 | 6,920000E-004 | Rho_family_GTPase_1 |
| EIF1 | 3,715703E-000 | 0,000000E+001 | 2,381000E-003 | putative_translation_initiation_factor |
| PSMD10 | 3,716780E-000 | 0,000000E+001 | 1,730000E-004 | proteasome_(prosome_macropain)_26S_subunit_non-ATPase_10 |
| PEX6 | 3,725582E-000 | 0,000000E+001 | 1,000000E-004 | peroxisomal_biogenesis_factor_6 |
| GNB1 | 3,736869E-000 | 2,000000E-006 | 4,873000E-002 | guanine_nucleotide_binding_protein_(G_protein)_beta_polypeptide_1 |
| PPP1R8 | 3,737732E-000 | 0,000000E+001 | 6,532000E-003 | protein_phosphatase_1_regulatory_(inhibitor)_subunit_8 |
| H3F3B | 3,758615E-000 | 0,000000E+001 | 7,200000E-005 | H3_histone_family_3B_(H3,3B) |
| DOCK10 | 3,765044E-000 | 0,000000E+001 | 1,310000E-003 | dedicator_of_cytokinesis_10 |
| DLG7 | 3,800369E-000 | 1,000000E-006 | 1,947700E-002 | discs_large_homolog_7_(Drosophila) |
| RFX5 | 3,813342E-000 | 0,000000E+001 | 2,210000E-004 | regulatory_factor_X_5_(influences_HLA_class_II_expression) |
| PLEKHM2 | 3,849345E-000 | 0,000000E+001 | 8,954000E-003 | pleckstrin_homology_domain_containing_family_M_(with_RUN_domain)_member_2 |
| FBXO42 | 3,854317E-000 | 2,000000E-006 | 4,592700E-002 | F-box_protein_42 |
| NPM1 | 3,901070E-000 | 0,000000E+001 | 1,575000E-003 | nucleophosmin_(nucleolar_phosphoprotein_B23_numatrin) |
| EEF2 | 3,913004E-000 | 0,000000E+001 | 3,500000E-005 | eukaryotic_translation_elongation_factor_2 |
| ZDHHC4 | 4,029381E-000 | 1,000000E-006 | 1,771800E-002 | zinc_finger_DHHC-type_containing_4 |
| GLS | 4,036685E-000 | 0,000000E+001 | 3,165000E-003 | glutaminase |
| THAP1 | 4,037367E-000 | 0,000000E+001 | 3,830000E-004 | THAP_domain_containing_apoptosis_associated_protein_1 |
| GORASP2 | 4,038991E-000 | 0,000000E+001 | 5,000000E-004 | golgi_reassembly_stacking_protein_2_55kDa |
| FZD1 | 4,040570E-000 | 0,000000E+001 | 7,720000E-004 | frizzled_homolog_1_(Drosophila) |
| FAM45B | 4,082189E-000 | 1,000000E-006 | 1,563200E-002 | family_with_sequence_similarity_45_member_B |
| CLIC3 | 4,085276E-000 | 0,000000E+001 | 2,744000E-003 | chloride_intracellular_channel_3 |
| SLC29A3 | 4,088489E-000 | 0,000000E+001 | 4,209000E-003 | solute_carrier_family_29_(nucleoside_transporters)_member_3 |
| RABEP2 | 4,103026E-000 | 0,000000E+001 | 6,450000E-004 | rabaptin_RAB_GTPase_binding_effector_protein_2 |
| KIAA1509 | 4,113277E-000 | 0,000000E+001 | 9,000000E-006 | KIAA1509 |
| TPK1 | 4,134051E-000 | 0,000000E+001 | 1,100000E-004 | thiamin_pyrophosphokinase_1 |
| FARSLA | 4,138206E-000 | 1,000000E-006 | 2,320800E-002 | phenylalanine-tRNA_synthetase-like_alpha_subunit |
| EXOC2 | 4,163767E-000 | 0,000000E+001 | 7,740000E-004 | SEC5-like_1_(S,_cerevisiae) |
| ATM | 4,169221E-000 | 1,000000E-006 | 1,654500E-002 | ataxia_telangiectasia_mutated_(includes_complementation_groups_A_C_and_D) |
| ZZEF1 | 4,215854E-000 | 0,000000E+001 | 0,000000E+001 | zinc_finger_ZZ-type_with_EF-hand_domain_1 |
| CITED1 | 4,221874E-000 | 0,000000E+001 | 3,049000E-003 | Cbp/p300-interacting_transactivator_with_Glu/Asp-rich_carboxy-terminal_domain_1 |
| RAD51AP1 | 4,244357E-000 | 0,000000E+001 | 7,287000E-003 | RAD51_associated_protein_1 |
| FCAR | 4,278978E-000 | 0,000000E+001 | 2,830000E-004 | Fc_fragment_of_IgA_receptor_for |
| BTN2A1 | 4,283194E-000 | 0,000000E+001 | 1,578000E-003 | butyrophilin_subfamily_2_member_A1 |
| TRIB2 | 4,295465E-000 | 0,000000E+001 | 1,294000E-003 | tribbles_homolog_2_(Drosophila) |
| SLC35E2 | 4,304800E-000 | 0,000000E+001 | 8,800000E-005 | succinate_dehydrogenase_complex_subunit_D_integral_membrane_protein |
| KIAA0286 | 4,312095E-000 | 1,000000E-006 | 1,361400E-002 | KIAA0286_protein |
| SPAST | 4,313284E-000 | 1,000000E-006 | 1,881000E-002 | spastin |
| TBL1XR1 | 4,318592E-000 | 1,000000E-006 | 1,817300E-002 | transducin_(beta)-like_1X-linked_receptor_1 |
| NFIL3 | 4,333939E-000 | 0,000000E+001 | 1,064200E-002 | nuclear_factor_interleukin_3_regulated |
| NR4A2 | 4,346949E-000 | 0,000000E+001 | 6,364000E-003 | nuclear_receptor_subfamily_4_group_A_member_2 |
| CRTAM | 4,351147E-000 | 0,000000E+001 | 9,790000E-004 | class-I_MHC-restricted_T_cell_associated_molecule |
| CEBPZ | 4,355262E-000 | 0,000000E+001 | 6,950000E-004 | CCAAT/enhancer_binding_protein_zeta |
| RNF170 | 4,408029E-000 | 0,000000E+001 | 1,634000E-003 | ring_finger_protein_170 |
| DHX34 | 4,430615E-000 | 0,000000E+001 | 9,548000E-003 | DEAH_(Asp-Glu-Ala-His)_box_polypeptide_34 |
| EEF1G | 4,430772E-000 | 0,000000E+001 | 4,037000E-003 | eukaryotic_translation_elongation_factor_1_gamma |
| EIF4G1 | 4,434509E-000 | 2,000000E-006 | 5,063400E-002 | eukaryotic_translation_initiation_factor_4_gamma_1 |
| DAZAP1 | 4,484252E-000 | 0,000000E+001 | 7,606000E-003 | DAZ_associated_protein_1 |
| ITGA4 | 4,494922E-000 | 0,000000E+001 | 1,790000E-004 | integrin_alpha_4_(antigen_CD49D_alpha_4_subunit_of_VLA-4_receptor) |
| DDIT4 | 4,561275E-000 | 0,000000E+001 | 7,000000E-006 | DNA-damage-inducible_transcript_4 |
| CLN5 | 4,562669E-000 | 0,000000E+001 | 8,590000E-004 | ceroid-lipofuscinosis_neuronal_5 |
| YME1L1 | 4,563175E-000 | 0,000000E+001 | 8,067000E-003 | YME1-like_1_(S,_cerevisiae) |
| NBN | 4,623520E-000 | 0,000000E+001 | 5,080000E-004 | nibrin |
| WWC3 | 4,629853E-000 | 1,000000E-006 | 1,938400E-002 | KIAA1280_protein |
| RGL1 | 4,639089E-000 | 0,000000E+001 | 2,780000E-004 | ral_guanine_nucleotide_dissociation_stimulator-like_1 |
| KLHL18 | 4,681089E-000 | 0,000000E+001 | 4,493000E-003 | kelch-like_18_(Drosophila) |
| SKP1A | 4,727158E-000 | 1,000000E-006 | 2,589000E-002 | S-phase_kinase-associated_protein_1A_(p19A) |
| MAT2A | 4,743715E-000 | 0,000000E+001 | 1,110000E-003 | methionine_adenosyltransferase_II_alpha |
| RTP4 | 4,812690E-000 | 1,000000E-006 | 1,821700E-002 | 28kD_interferon_responsive_protein |
| YIPF1 | 4,845599E-000 | 2,000000E-006 | 5,019700E-002 | Yip1_domain_family_member_1 |
| PAPSS1 | 4,857044E-000 | 0,000000E+001 | 5,227000E-003 | 3'-phosphoadenosine_5'-phosphosulfate_synthase_1 |
| BHLHB3 | 4,903332E-000 | 0,000000E+001 | 2,000000E-005 | basic_helix-loop-helix_domain_containing_class_B_3 |
| RBMS1 | 4,963649E-000 | 1,000000E-006 | 1,626800E-002 | RNA_binding_motif_single_stranded_interacting_protein_1 |
| PSCD1 | 4,992970E-000 | 0,000000E+001 | 7,000000E-006 | pleckstrin_homology_Sec7_and_coiled-coil_domains_1(cytohesin_1) |
| NXT2 | 5,011391E-000 | 1,000000E-006 | 2,301200E-002 | nuclear_transport_factor_2-like_export_factor_2 |
| HARSL | 5,031906E-000 | 0,000000E+001 | 4,700000E-005 | histidyl-tRNA_synthetase-like |
| KIF11 | 5,047912E-000 | 1,000000E-006 | 3,245900E-002 | kinesin_family_member_11 |
| RALBP1 | 5,070224E-000 | 0,000000E+001 | 1,400000E-005 | ralA_binding_protein_1 |
| EIF1 | 5,129133E-000 | 0,000000E+001 | 2,080000E-003 | putative_translation_initiation_factor |
| GADD45B | 5,185130E-000 | 0,000000E+001 | 2,320000E-004 | growth_arrest_and_DNA-damage-inducible_beta |
| TUBB2A | 5,230437E-000 | 0,000000E+001 | 5,964000E-003 | tubulin_beta_2 |
| NR4A2 | 5,390137E-000 | 1,000000E-006 | 2,498000E-002 | nuclear_receptor_subfamily_4_group_A_member_2 |
| GADD45B | 5,402841E-000 | 1,000000E-006 | 1,771700E-002 | growth_arrest_and_DNA-damage-inducible_beta |
| ZNF419 | 5,444360E-000 | 2,000000E-006 | 4,941000E-002 | zinc_finger_protein_419 |
| ATM | 5,445352E-000 | 0,000000E+001 | 1,090000E-004 | ataxia_telangiectasia_mutated_(includes_complementation_groups_A_C_and_D) |
| RS1 | 5,478536E-000 | 1,000000E-006 | 3,045300E-002 | retinoschisis_(X-linked_juvenile)_1 |
| PPP1R15A | 5,525747E-000 | 0,000000E+001 | 2,439000E-003 | protein_phosphatase_1_regulatory_(inhibitor)_subunit_15A |
| RPL27 | 5,618427E-000 | 0,000000E+001 | 7,170000E-004 | ribosomal_protein_L27 |
| ADAMTSL2 | 5,619745E-000 | 0,000000E+001 | 1,027900E-002 | ADAMTS-like_2 |
| HIST1H1D | 5,629997E-000 | 2,000000E-006 | 5,090800E-002 | histone_1_H1d |
| APLP2 | 5,648753E-000 | 0,000000E+001 | 2,384000E-003 | amyloid_beta_(A4)_precursor-like_protein_2 |
| TRIM2 | 5,778278E-000 | 1,000000E-006 | 1,630900E-002 | tripartite_motif-containing_2 |
| ATG5 | 5,803106E-000 | 0,000000E+001 | 9,800000E-005 | APG5_autophagy_5-like_(S,_cerevisiae) |
| PGGT1B | 5,878115E-000 | 0,000000E+001 | 7,684000E-003 | protein_geranylgeranyltransferase_type_I_beta_subunit |
| CCNA2 | 5,948745E-000 | 0,000000E+001 | 4,380000E-004 | cyclin_A2 |
| IER2 | 6,102851E-000 | 0,000000E+001 | 5,460000E-004 | immediate_early_response_2 |
| CREBL2 | 6,126065E-000 | 1,000000E-006 | 2,574400E-002 | cAMP_responsive_element_binding_protein-like_2 |
| ABCD2 | 6,167997E-000 | 0,000000E+001 | 1,134000E-003 | ATP-binding_cassette_sub-family_D_(ALD)_member_2 |
| TMOD2 | 6,173326E-000 | 0,000000E+001 | 2,557000E-003 | tropomodulin_2_(neuronal) |
| TRIM34 | 6,177888E-000 | 0,000000E+001 | 2,430000E-003 | tripartite_motif-containing_34 |
| PDE4B | 6,301159E-000 | 0,000000E+001 | 4,806000E-003 | phosphodiesterase_4B_cAMP-specific_(phosphodiesterase_E4_dunce_homolog_Drosophila) |
| PDK2 | 6,433011E-000 | 0,000000E+001 | 6,498000E-003 | pyruvate_dehydrogenase_kinase_isoenzyme_2 |
| HNRPC | 6,481214E-000 | 2,000000E-006 | 3,946800E-002 | heterogeneous_nuclear_ribonucleoprotein_C_(C1/C2) |
| ATP6V1C1 | 6,535739E-000 | 0,000000E+001 | 3,700000E-005 | ATPase_H+_transporting_lysosomal_42kDa_V1_subunit_C_isoform_1 |
| VRK3 | 6,594590E-000 | 0,000000E+001 | 2,662000E-003 | vaccinia_related_kinase_3 |
| GLRA3 | 6,706035E-000 | 0,000000E+001 | 7,954000E-003 | glycine_receptor_alpha_3 |
| SFRS2IP | 6,713926E-000 | 0,000000E+001 | 7,250000E-004 | splicing_factor_arginine/serine-rich_2_interacting_protein |
| PRDX6 | 6,723850E-000 | 1,000000E-006 | 1,411700E-002 | peroxiredoxin_6 |
| TARBP1 | 6,886174E-000 | 0,000000E+001 | 9,618000E-003 | TAR_(HIV)_RNA_binding_protein_1 |
| ABCD3 | 6,962743E-000 | 0,000000E+001 | 2,234000E-003 | ATP-binding_cassette_sub-family_D_(ALD)_member_3 |
| TRPC1 | 6,965986E-000 | 0,000000E+001 | 1,730000E-004 | transient_receptor_potential_cation_channel_subfamily_C_member_1 |
| FANCF | 7,067530E-000 | 2,000000E-006 | 3,449600E-002 | Fanconi_anemia_complementation_group_F |
| JUNB | 7,540511E-000 | 0,000000E+001 | 1,800000E-005 | jun_B_proto-oncogene |
| APBA3 | 7,696288E-000 | 1,000000E-006 | 3,225300E-002 | amyloid_beta_(A4)_precursor_protein-binding_family_A_member_3_(X11-like_2) |
| DHPS | 7,772675E-000 | 0,000000E+001 | 4,960000E-004 | deoxyhypusine_synthase |
| NOL1 | 8,091880E-000 | 1,000000E-006 | 2,439000E-002 | nucleolar_protein_1_120kDa |
| ATXN1 | 8,278727E-000 | 0,000000E+001 | 3,162000E-003 | ataxin_1 |
| PPP2R1B | 8,692650E-000 | 1,000000E-006 | 1,729200E-002 | protein_phosphatase_2_(formerly_2A)_regulatory_subunit_A_(PR_65)_beta_isoform |
| CDKN1A | 8,812104E-000 | 2,000000E-006 | 4,294200E-002 | cyclin-dependent_kinase_inhibitor_1A_(p21_Cip1) |
| MXD1 | 8,860129E-000 | 0,000000E+001 | 7,035000E-003 | MAX_dimerization_protein_1 |
| CREBL2 | 8,945460E-000 | 1,000000E-006 | 3,062700E-002 | cAMP_responsive_element_binding_protein-like_2 |
| RPL17 | 9,098620E-000 | 0,000000E+001 | 3,500000E-005 | ribosomal_protein_L17 |
| ITGB2 | 9,228345E-000 | 0,000000E+001 | 6,850000E-004 | integrin_beta_2_(antigen_CD18_(p95)_lymphocyte_function-associated_antigen_1_macrophage_antigen_1 |
| GCC2 | 9,246139E-000 | 0,000000E+001 | 3,460000E-004 | GRIP_and_coiled-coil_domain_containing_2 |
| TBCB | 9,259874E-000 | 2,000000E-006 | 3,585500E-002 | cytoskeleton_associated_protein_1 |
| DEFA1 | 9,649008E-000 | 0,000000E+001 | 8,356000E-003 | defensin_alpha_1_myeloid-related_sequence |
| SLC9A6 | 9,694874E-000 | 0,000000E+001 | 3,463000E-003 | solute_carrier_family_9_(sodium/hydrogen_exchanger)_isoform_6 |
| GAPDH | 9,891871E-000 | 0,000000E+001 | 4,002000E-003 | glyceraldehyde-3-phosphate_dehydrogenase |
| SLC1A3 | 1,011989E+001 | 0,000000E+001 | 1,370000E-003 | solute_carrier_family_1_(glial_high_affinity_glutamate_transporter)_member_3 |
| DNAJC10 | 1,026115E+001 | 0,000000E+001 | 1,203000E-003 | DnaJ_(Hsp40)_homolog_subfamily_C_member_10 |
| FAM89B | 1,100626E+001 | 2,000000E-006 | 4,290600E-002 | Mouse_Mammary_Turmor_Virus_Receptor_homolog_1 |
| CC2D1A | 1,107168E+001 | 0,000000E+001 | 6,620000E-004 | coiled-coil_and_C2_domain_containing_1A |
| CD93 | 1,117101E+001 | 1,000000E-006 | 2,989000E-002 | complement_component_1_q_subcomponent_receptor_1 |
| VPS35 | 1,121137E+001 | 0,000000E+001 | 6,820000E-003 | vacuolar_protein_sorting_35_(yeast) |
| N4BP1 | 1,123556E+001 | 0,000000E+001 | 3,000000E-006 | Nedd4_binding_protein_1 |
| ASCC3 | 1,153723E+001 | 0,000000E+001 | 5,160000E-003 | activating_signal_cointegrator_1_complex_subunit_3 |
| MLSTD1 | 1,187045E+001 | 2,000000E-006 | 3,470200E-002 | male_sterility_domain_containing_1 |
| SLC7A8 | 1,206155E+001 | 0,000000E+001 | 5,800000E-005 | Solute_carrier_family_7_(cationic_amino_acid_transporter_y+_system)_member_8 |
| CHRNA4 | 1,226468E+001 | 0,000000E+001 | 8,796000E-003 | cholinergic_receptor_nicotinic_alpha_polypeptide_4 |
| PARD3 | 1,266274E+001 | 0,000000E+001 | 1,206000E-003 | par-3_partitioning_defective_3_homolog_(C,_elegans) |
| DNAJB12 | 1,322107E+001 | 0,000000E+001 | 1,900000E-005 | DnaJ_(Hsp40)_homolog_subfamily_B_member_12 |
| FLT3LG | 1,657796E+001 | 1,000000E-006 | 1,145300E-002 | fms-related_tyrosine_kinase_3_ligand |
| INSIG2 | 1,735155E+001 | 0,000000E+001 | 3,200000E-005 | insulin_induced_gene_2 |
| LPP | 1,895813E+001 | 1,000000E-006 | 1,297000E-002 | LIM_domain_containing_preferred_translocation_partner_in_lipoma |
| MYH13 | 1,971273E+001 | 2,000000E-006 | 4,347100E-002 | myosin_heavy_polypeptide_13_skeletal_muscle |
| E2F1 | 2,184466E+001 | 0,000000E+001 | 6,699000E-003 | E2F_transcription_factor_1 |
| EPOR | 2,233566E+001 | 0,000000E+001 | 8,286000E-003 | erythropoietin_receptor |
| TCEB1 | 2,533378E+001 | 0,000000E+001 | 3,010000E-004 | transcription_elongation_factor_B_(SIII)_polypeptide_1_(15kDa_elongin_C) |
| NIT1 | 2,879983E+001 | 0,000000E+001 | 3,812000E-003 | nitrilase_1 |
| PCNXL2 | 3,057569E+001 | 0,000000E+001 | 7,746000E-003 | pecanex-like_2_(Drosophila) |
| BSCL2 | 3,700808E+001 | 2,000000E-006 | 3,869700E-002 | Bernardinelli-Seip_congenital_lipodystrophy_2_(seipin) |
| TXNL4A | 1,247526E+002 | 0,000000E+001 | 7,476000E-003 | thioredoxin-like_4A |
| NOL7 | 1,966023E+002 | 0,000000E+001 | 1,856000E-003 | nucleolar_protein_7_27kDa |

# Supplementary Table 1B: Genes affected by intra-group, inter-individual mRNA expression variances (OA compared to NC):

**higher variances in OA**

| **GeneSymbol** | **variance Fold** | ***P* Brown-Forsythe** | ***P* Bonferroni** | **Full Annotation** |
| --- | --- | --- | --- | --- |
| N-PAC | 6,401726E+001 | 0,000000E+001 | 1,213000E-003 | cytokine-like_nuclear_factor_n-pac |
| PTPN12 | 5,205150E+001 | 0,000000E+001 | 7,813000E-003 | protein_tyrosine_phosphatase_non-receptor_type_12 |
| AKT2 | 4,404530E+001 | 0,000000E+001 | 3,030000E-004 | v-akt_murine_thymoma_viral_oncogene_homolog_2 |
| IKBKB | 1,837756E+001 | 0,000000E+001 | 5,470000E-004 | inhibitor_of_kappa_light_polypeptide_gene_enhancer_in_B-cells_kinase_beta |
| UNC45A | 1,689037E+001 | 0,000000E+001 | 2,500000E-004 | smooth_muscle_cell_associated_protein-1 |
| CDC2L1 | 1,605198E+001 | 0,000000E+001 | 4,709000E-003 | cell_division_cycle_2-like_1_(PITSLRE_proteins) |
| TNPO1 | 1,565616E+001 | 0,000000E+001 | 7,500000E-005 | transportin_1 |
| GJB1 | 1,535261E+001 | 0,000000E+001 | 3,360000E-004 | gap_junction_protein_beta_1_32kDa |
| SMARCC1 | 1,531468E+001 | 0,000000E+001 | 1,930000E-004 | SWI/SNF_related_matrix_associated_actin_dependent_regulator_of_chromatin  _subfamily_c_member_1 |
| ZFP36L2 | 1,475046E+001 | 0,000000E+001 | 2,357000E-003 | zinc_finger_protein_36_C3H_type-like_2 |
| IPO8 | 1,455783E+001 | 1,000000E-006 | 3,232600E-002 | importin_8 |
| AD7C-NTP | 1,399150E+001 | 0,000000E+001 | 7,500000E-005 | neuronal_thread_protein_AD7c-NTP |
| NF1 | 1,341154E+001 | 1,000000E-006 | 1,704000E-002 | Neurofibromin_1_(neurofibromatosis_von_Recklinghausen_disease_Watson_disease) |
| TAF1C | 1,322743E+001 | 1,000000E-006 | 2,446500E-002 | TATA_box_binding_protein_(TBP)-associated_factor_RNA_polymerase_I_C_110kDa |
| EPN1 | 1,311582E+001 | 0,000000E+001 | 3,191000E-003 | epsin_1 |
| H2AFX | 1,232201E+001 | 1,000000E-006 | 3,009100E-002 | H2A_histone_family_member_X |
| SDCCAG3 | 1,186882E+001 | 1,000000E-006 | 2,929300E-002 | serologically_defined_colon_cancer_antigen_3 |
| STX16 | 1,170421E+001 | 0,000000E+001 | 2,200000E-005 | syntaxin_16 |
| GTF2I | 1,112480E+001 | 0,000000E+001 | 5,258000E-003 | general_transcription_factor_II_i |
| TOB2 | 1,064058E+001 | 1,000000E-006 | 1,338700E-002 | transducer_of_ERBB2_2 |
| DCLRE1C | 1,055735E+001 | 0,000000E+001 | 2,290000E-004 | DNA_cross-link_repair_1C_(PSO2_homolog_S,_cerevisiae) |
| POLDIP3 | 9,467537E000 | 1,000000E-006 | 1,740800E-002 | polymerase_(DNA-directed)_delta_interacting_protein_3 |
| CSPP1 | 9,393090E000 | 0,000000E+001 | 1,785000E-003 | centrosome_spindle_pole_associated_protein |
| GNL3L | 9,306212E000 | 0,000000E+001 | 1,082200E-002 | guanine_nucleotide_binding_protein-like_3_(nucleolar)-like |
| CUGBP1 | 9,295015E000 | 0,000000E+001 | 4,700000E-005 | CUG_triplet_repeat_RNA_binding_protein_1 |
| TMEM126B | 9,013228E000 | 0,000000E+001 | 5,469000E-003 | uncharacterized_hypothalamus_protein_HT007 |
| RPL18 | 8,979441E000 | 2,000000E-006 | 4,231800E-002 | Ribosomal_protein_L18 |
| DNAJC3 | 8,907067E000 | 0,000000E+001 | 1,583000E-003 | DnaJ_(Hsp40)_homolog_subfamily_C_member_3 |
| SLC26A10 | 8,659990E000 | 0,000000E+001 | 6,200000E-005 | solute_carrier_family_26_member_10 |
| MAPKBP1 | 8,630762E000 | 0,000000E+001 | 2,790000E-003 | mitogen_activated_protein_kinase_binding_protein_1 |
| ACP1 | 8,483850E000 | 0,000000E+001 | 8,093000E-003 | acid_phosphatase_1_soluble |
| RAD1 | 8,234761E000 | 0,000000E+001 | 5,547000E-003 | RAD1_homolog_(S,_pombe) |
| GGA3 | 8,034893E000 | 1,000000E-006 | 3,134900E-002 | golgi_associated_gamma_adaptin_ear_containing_ARF_binding_protein_3 |
| DOCK10 | 7,996336E000 | 1,000000E-006 | 2,328700E-002 | dedicator_of_cytokinesis_10 |
| DHX9 | 7,897583E000 | 0,000000E+001 | 4,100000E-005 | DEAH_(Asp-Glu-Ala-His)_box_polypeptide_9 |
| SFI1 | 7,891262E000 | 0,000000E+001 | 2,670000E-004 | Sfi1_homolog_spindle_assembly_associated_(yeast) |
| ATN1 | 7,857494E000 | 0,000000E+001 | 3,549000E-003 | Atrophin_1 |
| FAM48A | 7,585378E000 | 0,000000E+001 | 1,793000E-003 | family_with_sequence_similarity_48_member_A |
| B4GALT1 | 7,551318E000 | 0,000000E+001 | 1,435000E-003 | UDP-Gal:betaGlcNAc_beta_14-_galactosyltransferase_polypeptide_1 |
| WIZ | 7,480995E000 | 0,000000E+001 | 1,105000E-003 | widely-interspaced_zinc_finger_motifs |
| BCL2L1 | 7,251270E000 | 0,000000E+001 | 4,052000E-003 | BCL2-like_1 |
| CSNK2A1 | 7,221506E000 | 0,000000E+001 | 1,950000E-004 | casein_kinase_2_alpha_1_polypeptide |
| EPRS | 7,148906E000 | 0,000000E+001 | 4,650000E-004 | glutamyl-prolyl-tRNA_synthetase |
| GTSE1 | 7,117935E000 | 0,000000E+001 | 1,400000E-005 | G-2_and_S-phase_expressed_1 |
| RAB35 | 7,039969E000 | 0,000000E+001 | 7,000000E-006 | RAB35_member_RAS_oncogene_family |
| KLHL20 | 6,901546E000 | 1,000000E-006 | 2,203000E-002 | kelch-like_20_(Drosophila) |
| RPS10 | 6,785486E000 | 1,000000E-006 | 3,192000E-002 | Ribosomal_protein_S10 |
| PDCD6 | 6,667720E000 | 0,000000E+001 | 7,000000E-006 | programmed_cell_death_6 |
| GSN | 6,578569E000 | 0,000000E+001 | 1,800000E-005 | gelsolin_(amyloidosis_Finnish_type) |
| BICD1 | 6,524086E000 | 0,000000E+001 | 3,040000E-003 | bicaudal_D_homolog_1_(Drosophila) |
| TUBGCP2 | 6,480113E000 | 0,000000E+001 | 4,763000E-003 | tubulin_gamma_complex_associated_protein_2 |
| GNRH2 | 6,469277E000 | 0,000000E+001 | 4,350000E-003 | gonadotropin-releasing_hormone_2 |
| ATP2A2 | 6,459284E000 | 0,000000E+001 | 2,200000E-005 | ATPase_Ca++_transporting_cardiac_muscle_slow_twitch_2 |
| FRMD4A | 6,431391E000 | 0,000000E+001 | 6,810000E-004 | FERM_domain_containing_4A |
| CTGLF1 | 6,318398E000 | 0,000000E+001 | 1,460000E-004 | centaurin_gamma-like_family_member_1 |
| SPTBN1 | 6,307515E000 | 0,000000E+001 | 3,020000E-004 | spectrin_beta_non-erythrocytic_1 |
| HMGCS1 | 6,305200E000 | 0,000000E+001 | 9,940000E-004 | 3-hydroxy-3-methylglutaryl-Coenzyme_A_synthase_1_(soluble) |
| SMA4 | 6,270770E000 | 2,000000E-006 | 3,480100E-002 | SMA4 |
| TAF6L | 6,259126E000 | 2,000000E-006 | 4,700800E-002 | TAF6-like_RNA_polymerase_II_p300/CBP-associated_factor |
| EEF1D | 6,198164E000 | 1,000000E-006 | 3,154800E-002 | Eukaryotic_translation_elongation_factor_1_delta_(guanine_nucleotide_exchange_protein) |
| NCR2 | 6,173285E000 | 1,000000E-006 | 2,014100E-002 | natural_cytotoxicity_triggering_receptor_2 |
| ILF3 | 6,142616E000 | 0,000000E+001 | 1,960000E-004 | interleukin_enhancer_binding_factor_3_90kDa |
| FOXJ3 | 6,114959E000 | 0,000000E+001 | 4,317000E-003 | forkhead_box_J3 |
| SFRS8 | 6,107399E000 | 0,000000E+001 | 3,300000E-005 | splicing_factor_arginine/serine-rich_8_(suppressor-of-white-apricot_homolog_Drosophila) |
| SHMT1 | 6,037195E000 | 0,000000E+001 | 3,632000E-003 | serine_hydroxymethyltransferase_1_(soluble) |
| PRKACA | 5,990959E000 | 0,000000E+001 | 1,990000E-004 | protein_kinase_cAMP-dependent_catalytic_alpha |
| ADAM15 | 5,984811E000 | 0,000000E+001 | 2,850000E-004 | a_disintegrin_and_metalloproteinase_domain_15_(metargidin) |
| MRPL18 | 5,951351E000 | 0,000000E+001 | 3,642000E-003 | mitochondrial_ribosomal_protein_L18 |
| PRDX2 | 5,935944E000 | 0,000000E+001 | 2,260000E-004 | peroxiredoxin_2 |
| DIAPH2 | 5,862583E000 | 0,000000E+001 | 7,728000E-003 | diaphanous_homolog_2_(Drosophila) |
| SRRM2 | 5,855487E000 | 0,000000E+001 | 1,046200E-002 | Serine/arginine_repetitive_matrix_2 |
| PPIA | 5,687417E000 | 2,000000E-006 | 3,499800E-002 | peptidylprolyl_isomerase_A_(cyclophilin_A) |
| SS18 | 5,686692E000 | 0,000000E+001 | 8,756000E-003 | synovial_sarcoma_translocation_chromosome_18 |
| SFI1 | 5,678846E000 | 0,000000E+001 | 1,100000E-004 | Sfi1_homolog_spindle_assembly_associated_(yeast) |
| PRODH2 | 5,666881E000 | 0,000000E+001 | 1,631000E-003 | proline_dehydrogenase_(oxidase)_2 |
| WDFY3 | 5,633428E000 | 0,000000E+001 | 1,676000E-003 | WD_repeat_and_FYVE_domain_containing_3 |
| NADK | 5,616677E000 | 0,000000E+001 | 1,720000E-003 | NAD_kinase |
| THOC2 | 5,588910E000 | 0,000000E+001 | 8,200000E-005 | THO_complex_2 |
| SRPR | 5,566594E000 | 0,000000E+001 | 9,400000E-005 | signal_recognition_particle_receptor_('docking_protein') |
| RANBP2 | 5,547101E000 | 0,000000E+001 | 8,000000E-005 | RAN_binding_protein_2 |
| FTO | 5,527121E000 | 0,000000E+001 | 9,690000E-003 | Fatso |
| B2M | 5,458789E000 | 0,000000E+001 | 4,200000E-005 | beta-2-microglobulin |
| RBBP6 | 5,375650E000 | 0,000000E+001 | 2,489000E-003 | retinoblastoma_binding_protein_6 |
| PAPOLA | 5,367479E000 | 2,000000E-006 | 4,614100E-002 | Poly(A)_polymerase_alpha |
| CDC2L5 | 5,323606E000 | 0,000000E+001 | 1,494000E-003 | cell_division_cycle_2-like_5_(cholinesterase-related_cell_division_controller) |
| RERE | 5,292552E000 | 0,000000E+001 | 4,351000E-003 | arginine-glutamic_acid_dipeptide_(RE)_repeats |
| TTF1 | 5,274610E000 | 0,000000E+001 | 5,272000E-003 | transcription_termination_factor_RNA_polymerase_I |
| KRTAP5-8 | 5,261242E000 | 0,000000E+001 | 1,200000E-005 | keratin_associated_protein_5-8 |
| RPA4 | 5,208935E000 | 1,000000E-006 | 1,969300E-002 | replication_protein_A4_34kDa |
| PLEC1 | 5,150647E000 | 0,000000E+001 | 1,859000E-003 | plectin_1_intermediate_filament_binding_protein_500kDa |
| PSMB4 | 5,134014E000 | 1,000000E-006 | 2,172500E-002 | proteasome_(prosome_macropain)_subunit_beta_type_4 |
| ANKHD1 | 5,132640E000 | 0,000000E+001 | 1,860000E-003 | ankyrin_repeat_and_KH_domain_containing_1 |
| CUGBP1 | 5,092863E000 | 0,000000E+001 | 2,410000E-004 | CUG_triplet_repeat_RNA_binding_protein_1 |
| ENG | 5,089958E000 | 0,000000E+001 | 5,950000E-004 | endoglin_(Osler-Rendu-Weber_syndrome_1) |
| APLP2 | 5,056475E000 | 0,000000E+001 | 8,200000E-005 | Amyloid_beta_(A4)_precursor-like_protein_2 |
| TEX261 | 5,054827E000 | 0,000000E+001 | 8,391000E-003 | testis_expressed_sequence_261 |
| FUT2 | 5,046775E000 | 0,000000E+001 | 9,112000E-003 | fucosyltransferase_2_(secretor_status_included) |
| ZDHHC11 | 5,043010E000 | 0,000000E+001 | 5,723000E-003 | zinc_finger_DHHC-type_containing_11 |
| MRPL49 | 5,023746E000 | 0,000000E+001 | 9,510000E-004 | mitochondrial_ribosomal_protein_L49 |
| GSK3A | 5,018842E000 | 0,000000E+001 | 2,509000E-003 | glycogen_synthase_kinase_3_alpha |
| ZBTB43 | 5,015581E000 | 1,000000E-006 | 2,176300E-002 | zinc_finger_protein_297B |
| TNPO3 | 4,998036E000 | 2,000000E-006 | 4,168400E-002 | transportin_3 |
| PPM1A | 4,995521E000 | 2,000000E-006 | 4,314900E-002 | protein_phosphatase_1A_(formerly_2C)_magnesium-dependent_alpha_isoform |
| GRB10 | 4,989459E000 | 0,000000E+001 | 2,730000E-004 | growth_factor_receptor-bound_protein_10 |
| N-PAC | 4,974992E000 | 0,000000E+001 | 5,800000E-005 | cytokine-like_nuclear_factor_n-pac |
| ZMYM2 | 4,953410E000 | 1,000000E-006 | 1,473500E-002 | zinc_finger_protein_198 |
| WDR68 | 4,928161E000 | 0,000000E+001 | 7,800000E-005 | WD_repeat_domain_68 |
| PAPD4 | 4,871590E000 | 0,000000E+001 | 8,720000E-004 | PAP_associated_domain_containing_4 |
| UBE2D2 | 4,755322E000 | 0,000000E+001 | 8,614000E-003 | ubiquitin-conjugating_enzyme_E2D_2_(UBC4/5_homolog_yeast) |
| NDUFB6 | 4,739738E000 | 1,000000E-006 | 1,324700E-002 | NADH_dehydrogenase_(ubiquinone)_1_beta_subcomplex_6_17kDa |
| MAP3K7IP2 | 4,732592E000 | 1,000000E-006 | 1,772600E-002 | mitogen-activated_protein_kinase_kinase_kinase_7_interacting_protein_2 |
| SH2D3A | 4,730173E000 | 0,000000E+001 | 2,066000E-003 | SH2_domain_containing_3A |
| PI4KII | 4,725773E000 | 0,000000E+001 | 1,293000E-003 | phosphatidylinositol_4-kinase_type_II |
| SPCS1 | 4,720767E000 | 0,000000E+001 | 6,990000E-004 | signal_peptidase_complex_subunit_1_homolog_(S,_cerevisiae) |
| SREBF2 | 4,714223E000 | 0,000000E+001 | 1,462000E-003 | sterol_regulatory_element_binding_transcription_factor_2 |
| BACH2 | 4,629782E000 | 0,000000E+001 | 3,600000E-005 | BTB_and_CNC_homology_1_basic_leucine_zipper_transcription_factor_2 |
| PAX2 | 4,612167E000 | 2,000000E-006 | 3,971100E-002 | paired_box_gene_2 |
| GM2A | 4,578118E000 | 1,000000E-006 | 1,996600E-002 | GM2_ganglioside_activator |
| KIAA0101 | 4,576351E000 | 0,000000E+001 | 1,464000E-003 | KIAA0101 |
| PKP4 | 4,564099E000 | 0,000000E+001 | 1,070000E-004 | plakophilin_4 |
| TTC3 | 4,547913E000 | 1,000000E-006 | 1,621600E-002 | tetratricopeptide_repeat_domain_3 |
| NADK | 4,535218E000 | 0,000000E+001 | 7,410000E-004 | NAD_kinase |
| ARS2 | 4,528867E000 | 0,000000E+001 | 5,200000E-005 | arsenate_resistance_protein_ARS2 |
| SMEK1 | 4,514189E000 | 0,000000E+001 | 9,200000E-005 | KIAA2010 |
| FRMD4A | 4,480770E000 | 2,000000E-006 | 3,446000E-002 | FERM_domain_containing_4A |
| CALR | 4,479599E000 | 0,000000E+001 | 3,500000E-004 | Calreticulin |
| ATBF1 | 4,413228E000 | 0,000000E+001 | 5,087000E-003 | AT-binding_transcription_factor_1 |
| GTPBP1 | 4,406373E000 | 0,000000E+001 | 6,473000E-003 | GTP_binding_protein_1 |
| ACBD3 | 4,381199E000 | 0,000000E+001 | 1,310000E-004 | acyl-Coenzyme_A_binding_domain_containing_3 |
| PLCG1 | 4,348701E000 | 2,000000E-006 | 5,014200E-002 | phospholipase_C_gamma_1 |
| ZFR | 4,329114E000 | 2,000000E-006 | 4,402800E-002 | zinc_finger_RNA_binding_protein |
| RPP30 | 4,305849E000 | 0,000000E+001 | 4,677000E-003 | ribonuclease_P/MRP_30kDa_subunit |
| SMAD2 | 4,295286E000 | 0,000000E+001 | 1,570000E-004 | SMAD_mothers_against_DPP_homolog_2_(Drosophila) |
| MSL3L1 | 4,237105E000 | 0,000000E+001 | 3,013000E-003 | male-specific_lethal_3-like_1_(Drosophila) |
| SARDH | 4,234305E000 | 1,000000E-006 | 1,866000E-002 | sarcosine_dehydrogenase |
| DNAJB6 | 4,228113E000 | 1,000000E-006 | 2,242100E-002 | DnaJ_(Hsp40)_homolog_subfamily_B_member_6 |
| ADRBK1 | 4,222631E000 | 0,000000E+001 | 3,738000E-003 | adrenergic_beta_receptor_kinase_1 |
| PPP2R5B | 4,207998E000 | 1,000000E-006 | 3,175500E-002 | protein_phosphatase_2_regulatory_subunit_B_(B56)_beta_isoform |
| MED6 | 4,202969E000 | 0,000000E+001 | 8,960000E-004 | mediator_of_RNA_polymerase_II_transcription_subunit_6_homolog_(yeast) |
| DOHH | 4,175215E000 | 0,000000E+001 | 1,118000E-003 | HEAT-like_(PBS_lyase)_repeat_containing_1 |
| PSME4 | 4,140334E000 | 0,000000E+001 | 9,291000E-003 | proteasome_(prosome_macropain)_activator_subunit_4 |
| SRRM2 | 4,135630E000 | 0,000000E+001 | 3,900000E-005 | serine/arginine_repetitive_matrix_2 |
| MRP63 | 4,130175E000 | 0,000000E+001 | 4,200000E-005 | mitochondrial_ribosomal_protein_63 |
| FN1 | 4,121037E000 | 0,000000E+001 | 1,540000E-004 | fibronectin_1 |
| IFI16 | 4,103706E000 | 0,000000E+001 | 2,400000E-005 | interferon_gamma-inducible_protein_16 |
| FN1 | 4,089342E000 | 0,000000E+001 | 7,800000E-005 | fibronectin_1 |
| RANGAP1 | 4,074637E000 | 0,000000E+001 | 1,100100E-002 | Ran_GTPase_activating_protein_1 |
| ADD1 | 4,045678E000 | 0,000000E+001 | 2,710000E-004 | adducin_1_(alpha) |
| HADHA | 4,032869E000 | 0,000000E+001 | 8,700000E-005 | hydroxyacyl-Coenzyme_A_dehydrogenase/3-ketoacyl-Coenzyme_A_thiolase/  enoyl-Coenzyme_A_hydratase |
| VPS13D | 4,000717E000 | 0,000000E+001 | 1,042000E-003 | vacuolar_protein_sorting_13D_(yeast) |
| ARL1 | 3,999143E000 | 0,000000E+001 | 1,400000E-005 | ADP-ribosylation_factor-like_1 |
| PFAAP5 | 3,962473E000 | 0,000000E+001 | 4,870000E-004 | Phosphonoformate_immuno-associated_protein_5 |
| PDLIM5 | 3,958700E000 | 0,000000E+001 | 2,650000E-004 | PDZ_and_LIM_domain_5 |
| MFN2 | 3,956425E000 | 0,000000E+001 | 4,370000E-004 | mitofusin_2 |
| WNK1 | 3,929459E000 | 0,000000E+001 | 8,930000E-004 | WNK_lysine_deficient_protein_kinase_1 |
| PBX1 | 3,926205E000 | 0,000000E+001 | 4,100000E-005 | Pre-B-cell_leukemia_transcription_factor_1 |
| APP | 3,910100E000 | 0,000000E+001 | 3,000000E-006 | amyloid_beta_(A4)_precursor_protein_(protease_nexin-II_Alzheimer_disease) |
| DICER1 | 3,909188E000 | 0,000000E+001 | 1,130000E-004 | Dicer1_Dcr-1_homolog_(Drosophila) |
| FER1L3 | 3,906855E000 | 0,000000E+001 | 1,087900E-002 | fer-1-like_3_myoferlin_(C,_elegans) |
| PHACTR1 | 3,843072E000 | 1,000000E-006 | 1,763300E-002 | phosphatase_and_actin_regulator_1 |
| NPIP | 3,817438E000 | 1,000000E-006 | 1,489800E-002 | nuclear_pore_complex_interacting_protein |
| IRF5 | 3,810276E000 | 1,000000E-006 | 1,388400E-002 | interferon_regulatory_factor_5 |
| HSPA4 | 3,801360E000 | 0,000000E+001 | 4,200000E-004 | heat_shock_70kDa_protein_4 |
| LRCH1 | 3,790951E000 | 0,000000E+001 | 5,836000E-003 | Leucine-rich_repeats_and_calponin_homology_(CH)_domain_containing_1 |
| SAFB | 3,772835E000 | 1,000000E-006 | 1,427400E-002 | scaffold_attachment_factor_B |
| FLOT2 | 3,770678E000 | 2,000000E-006 | 4,248000E-002 | flotillin_2 |
| MZF1 | 3,767414E000 | 0,000000E+001 | 3,645000E-003 | zinc_finger_protein_42_(myeloid-specific_retinoic_acid-responsive) |
| TOP1 | 3,708411E000 | 0,000000E+001 | 4,000000E-006 | topoisomerase_(DNA)_I |
| ABLIM2 | 3,703142E000 | 1,000000E-006 | 1,301300E-002 | Actin_binding_LIM_protein_family_member_2 |
| SETD8 | 3,690397E000 | 1,000000E-006 | 1,146600E-002 | PR/SET_domain_containing_protein_8 |
| TNFAIP1 | 3,685357E000 | 0,000000E+001 | 3,610000E-003 | tumor_necrosis_factor_alpha-induced_protein_1_(endothelial) |
| RIOK3 | 3,671998E000 | 0,000000E+001 | 3,646000E-003 | RIO_kinase_3_(yeast) |
| PHF20L1 | 3,670007E000 | 0,000000E+001 | 4,127000E-003 | PHD_finger_protein_20-like_1 |
| ZBTB20 | 3,650983E000 | 1,000000E-006 | 1,607200E-002 | zinc_finger_and_BTB_domain_containing_20 |
| TRIM23 | 3,630767E000 | 0,000000E+001 | 4,271000E-003 | tripartite_motif-containing_23 |
| COX17 | 3,621875E000 | 0,000000E+001 | 1,057000E-003 | COX17_homolog_cytochrome_c_oxidase_assembly_protein_(yeast) |
| MAP4 | 3,593254E000 | 2,000000E-006 | 4,993300E-002 | microtubule-associated_protein_4 |
| SIN3B | 3,581389E000 | 1,000000E-006 | 3,290100E-002 | SIN3_homolog_B_transcription_regulator_(yeast) |
| SSR4 | 3,572571E000 | 0,000000E+001 | 2,516000E-003 | signal_sequence_receptor_delta_(translocon-associated_protein_delta) |
| ANKRD11 | 3,541485E000 | 0,000000E+001 | 8,980000E-003 | ankyrin_repeat_domain_11 |
| EEF1A1 | 3,536560E000 | 0,000000E+001 | 5,300000E-005 | eukaryotic_translation_elongation_factor_1_alpha_1 |
| BAT2D1 | 3,502100E000 | 0,000000E+001 | 4,770000E-004 | BAT2_domain_containing_1 |
| ANAPC5 | 3,491375E000 | 0,000000E+001 | 1,701000E-003 | anaphase_promoting_complex_subunit_5 |
| TRAPPC4 | 3,487700E000 | 0,000000E+001 | 7,170000E-004 | trafficking_protein_particle_complex_4 |
| B2M | 3,474876E000 | 0,000000E+001 | 1,000000E-005 | beta-2-microglobulin |
| TGFBR2 | 3,466899E000 | 0,000000E+001 | 1,000000E-006 | transforming_growth_factor_beta_receptor_II_(70/80kDa) |
| DLG1 | 3,466053E000 | 0,000000E+001 | 8,990000E-003 | discs_large_homolog_1_(Drosophila) |
| SPN | 3,454488E000 | 0,000000E+001 | 6,650000E-004 | sialophorin_(gpL115_leukosialin_CD43) |
| STK4 | 3,435536E000 | 1,000000E-006 | 1,602800E-002 | serine/threonine_kinase_4 |
| UBR2 | 3,415714E000 | 0,000000E+001 | 9,700000E-005 | Ubiquitin_protein_ligase_E3_component_n-recognin_2 |
| CTGLF1 | 3,414360E000 | 1,000000E-006 | 1,248200E-002 | centaurin_gamma-like_family_member_1 |
| KIF25 | 3,404029E000 | 0,000000E+001 | 5,590000E-004 | kinesin_family_member_25 |
| NDUFA13 | 3,385188E000 | 0,000000E+001 | 3,844000E-003 | NADH_dehydrogenase_(ubiquinone)_1_alpha_subcomplex_13 |
| NRP2 | 3,375526E000 | 0,000000E+001 | 3,895000E-003 | neuropilin_2 |
| PRDM2 | 3,372291E000 | 0,000000E+001 | 1,464000E-003 | PR_domain_containing_2_with_ZNF_domain |
| VIL2 | 3,367859E000 | 0,000000E+001 | 2,730000E-004 | villin_2_(ezrin) |
| PCDHGC3 | 3,357024E000 | 0,000000E+001 | 8,600000E-005 | protocadherin_gamma_subfamily_C_3 |
| ACTR2 | 3,355326E000 | 0,000000E+001 | 6,000000E-005 | ARP2_actin-related_protein_2_homolog_(yeast) |
| SFRS4 | 3,352187E000 | 0,000000E+001 | 1,019000E-003 | splicing_factor_arginine/serine-rich_4 |
| RFTN1 | 3,343431E000 | 1,000000E-006 | 2,295700E-002 | Raft-linking_protein |
| WWC2 | 3,343371E000 | 1,000000E-006 | 2,937200E-002 | BH3-only_member_B_protein |
| PTPRA | 3,337505E000 | 0,000000E+001 | 5,300000E-005 | protein_tyrosine_phosphatase_receptor_type_A |
| SLC9A5 | 3,334809E000 | 1,000000E-006 | 1,781700E-002 | solute_carrier_family_9_(sodium/hydrogen_exchanger)_isoform_5 |
| KLF12 | 3,324782E000 | 2,000000E-006 | 3,817800E-002 | Kruppel-like_factor_12 |
| MCM4 | 3,308761E000 | 1,000000E-006 | 2,290900E-002 | MCM4_minichromosome_maintenance_deficient_4_(S,_cerevisiae) |
| TMEM1 | 3,294485E000 | 0,000000E+001 | 1,703000E-003 | transmembrane_protein_1 |
| IMP3 | 3,290291E000 | 2,000000E-006 | 4,346100E-002 | IMP3_U3_small_nucleolar_ribonucleoprotein_homolog_(yeast) |
| CSNK2A1 | 3,283961E000 | 0,000000E+001 | 6,050000E-004 | casein_kinase_2_alpha_1_polypeptide |
| CIAO1 | 3,269388E000 | 0,000000E+001 | 9,981000E-003 | WD_repeat_domain_39 |
| CHMP2B | 3,263312E000 | 0,000000E+001 | 3,140000E-003 | chromatin_modifying_protein_2B |
| ALPK1 | 3,261500E000 | 0,000000E+001 | 6,421000E-003 | alpha-kinase_1 |
| TXNIP | 3,260522E000 | 0,000000E+001 | 6,858000E-003 | thioredoxin_interacting_protein |
| SFRS10 | 3,256071E000 | 0,000000E+001 | 6,603000E-003 | splicing_factor_arginine/serine-rich_10_(transformer_2_homolog_Drosophila) |
| PBX2 | 3,252406E000 | 1,000000E-006 | 3,199500E-002 | pre-B-cell_leukemia_transcription_factor_2 |
| PHF21A | 3,246288E000 | 0,000000E+001 | 6,867000E-003 | PHD_finger_protein_21A |
| THRAP1 | 3,244284E000 | 1,000000E-006 | 2,038500E-002 | Thyroid_hormone_receptor_associated_protein_1 |
| UPF1 | 3,237985E000 | 1,000000E-006 | 2,126500E-002 | regulator_of_nonsense_transcripts_1 |
| PPP3CB | 3,233625E000 | 0,000000E+001 | 9,126000E-003 | Protein_phosphatase_3_catalytic_subunit_beta_isoform_(calcineurin_A_beta) |
| TNPO1 | 3,216748E000 | 0,000000E+001 | 4,600000E-004 | transportin_1 |
| ENDOD1 | 3,201317E000 | 1,000000E-006 | 1,334200E-002 | KIAA0830_protein |
| DNAJC7 | 3,200334E000 | 1,000000E-006 | 1,387300E-002 | 2'3'-cyclic_nucleotide_3'_phosphodiesterase |
| ARID5A | 3,155961E000 | 1,000000E-006 | 1,194700E-002 | AT_rich_interactive_domain_5A_(MRF1-like) |
| LTB4R | 3,152132E000 | 2,000000E-006 | 3,666100E-002 | leukotriene_B4_receptor |
| FN1 | 3,145287E000 | 0,000000E+001 | 2,370000E-004 | fibronectin_1 |
| U2AF2 | 3,138377E000 | 0,000000E+001 | 9,716000E-003 | U2_(RNU2)_small_nuclear_RNA_auxiliary_factor_2 |
| PRKCE | 3,122616E000 | 0,000000E+001 | 1,768000E-003 | Protein_kinase_C_epsilon |
| FBXW12 | 3,102417E000 | 0,000000E+001 | 3,100000E-005 | F-box_and_WD-40_domain_protein_12 |
| PAWR | 3,101662E000 | 2,000000E-006 | 4,839600E-002 | PRKC_apoptosis_WT1_regulator |
| EEF1A1 | 3,094394E000 | 0,000000E+001 | 3,500000E-005 | eukaryotic_translation_elongation_factor_1_alpha_1 |
| DYNLRB1 | 3,090937E000 | 0,000000E+001 | 1,049000E-003 | dynein_cytoplasmic_light_polypeptide_2A |
| MARS | 3,069142E000 | 0,000000E+001 | 2,150000E-004 | Methionine-tRNA_synthetase |
| BTRC | 3,065334E000 | 1,000000E-006 | 2,251400E-002 | beta-transducin_repeat_containing |
| SLC7A8 | 3,050437E000 | 0,000000E+001 | 1,500000E-005 | solute_carrier_family_7_(cationic_amino_acid_transporter_y+_system)_member_8 |
| HSF1 | 3,041713E000 | 0,000000E+001 | 2,220000E-004 | heat_shock_transcription_factor_1 |
| INPP4A | 3,039004E000 | 1,000000E-006 | 1,223500E-002 | inositol_polyphosphate-4-phosphatase_type_I_107kDa |
| ZC3H11A | 3,034820E000 | 0,000000E+001 | 8,010000E-004 | zinc_finger_CCCH-type_containing_11A |
| SMC5 | 3,032408E000 | 0,000000E+001 | 8,550000E-004 | SMC5_structural_maintenance_of_chromosomes_5-like_1_(yeast) |
| AIRE | 3,015228E000 | 1,000000E-006 | 1,195700E-002 | autoimmune_regulator |
| MTA1 | 3,012111E000 | 0,000000E+001 | 2,280000E-004 | metastasis_associated_1 |
| ORC5L | 3,008247E000 | 2,000000E-006 | 4,441500E-002 | origin_recognition_complex_subunit_5-like_(yeast) |
| GPHN | 2,989055E000 | 1,000000E-006 | 2,047200E-002 | Gephyrin |
| BAT2D1 | 2,979389E000 | 0,000000E+001 | 3,220000E-004 | BAT2_domain_containing_1 |
| SYNPO2 | 2,968810E000 | 0,000000E+001 | 4,200000E-003 | Synaptopodin_2 |
| CD40 | 2,968094E000 | 0,000000E+001 | 6,080000E-004 | CD40_antigen_(TNF_receptor_superfamily_member_5) |
| FARS2 | 2,940352E000 | 0,000000E+001 | 3,758000E-003 | phenylalanine-tRNA_synthetase_2_(mitochondrial) |
| BAZ2A | 2,939551E000 | 0,000000E+001 | 1,649000E-003 | bromodomain_adjacent_to_zinc_finger_domain_2A |
| RPL14 | 2,938461E000 | 0,000000E+001 | 2,749000E-003 | ribosomal_protein_L14 |
| TBL1X | 2,936767E000 | 0,000000E+001 | 2,530000E-004 | transducin_(beta)-like_1X-linked |
| PFAAP5 | 2,912070E000 | 2,000000E-006 | 4,171000E-002 | Phosphonoformate_immuno-associated_protein_5 |
| HAB1 | 2,911823E000 | 0,000000E+001 | 9,152000E-003 | B1_for_mucin |
| RAD50 | 2,909181E000 | 0,000000E+001 | 4,778000E-003 | RAD50_homolog_(S,_cerevisiae) |
| RBX1 | 2,896569E000 | 1,000000E-006 | 1,882300E-002 | ring-box_1 |
| RBM25 | 2,895889E000 | 0,000000E+001 | 1,100000E-005 | RNA_binding_motif_protein_25 |
| RPL38 | 2,892757E000 | 0,000000E+001 | 7,950000E-004 | Ribosomal_protein_L38 |
| CROP | 2,890716E000 | 0,000000E+001 | 4,470000E-004 | cisplatin_resistance-associated_overexpressed_protein |
| PRKAR2A | 2,889453E000 | 0,000000E+001 | 4,886000E-003 | protein_kinase_cAMP-dependent_regulatory_type_II_alpha |
| EPS15L1 | 2,879604E000 | 0,000000E+001 | 4,501000E-003 | epidermal_growth_factor_receptor_pathway_substrate_15-like_1 |
| CDC2L1 | 2,879484E000 | 0,000000E+001 | 1,544000E-003 | cell_division_cycle_2-like_1_(PITSLRE_proteins) |
| TOP3A | 2,870190E000 | 1,000000E-006 | 1,186500E-002 | topoisomerase_(DNA)_III_alpha |
| TMEM28 | 2,859561E000 | 2,000000E-006 | 4,827900E-002 | transmembrane_protein_28 |
| EPPB9 | 2,848603E000 | 1,000000E-006 | 1,139300E-002 | B9_protein |
| PNPLA4 | 2,844081E000 | 2,000000E-006 | 3,709000E-002 | patatin-like_phospholipase_domain_containing_4 |
| SLC26A1 | 2,807999E000 | 0,000000E+001 | 6,537000E-003 | solute_carrier_family_26_(sulfate_transporter)_member_1 |
| GATA2 | 2,797963E000 | 1,000000E-006 | 2,809600E-002 | GATA_binding_protein_2 |
| REPS1 | 2,793828E000 | 0,000000E+001 | 3,774000E-003 | RALBP1_associated_Eps_domain_containing_1 |
| NDUFS3 | 2,776551E000 | 0,000000E+001 | 1,914000E-003 | NADH_dehydrogenase_(ubiquinone)_Fe-S_protein_3_30kDa |
| PPFIA1 | 2,768906E000 | 1,000000E-006 | 1,372100E-002 | protein_tyrosine_phosphatase_receptor_type_f_polypeptide_interacting_protein_alpha |
| MZF1 | 2,766298E000 | 1,000000E-006 | 1,284900E-002 | zinc_finger_protein_42_(myeloid-specific_retinoic_acid-responsive) |
| TPP1 | 2,766294E000 | 0,000000E+001 | 2,049000E-003 | tripeptidyl_peptidase_I |
| TNPO1 | 2,760859E000 | 0,000000E+001 | 1,700000E-005 | transportin_1 |
| PSMB1 | 2,741466E000 | 1,000000E-006 | 1,534500E-002 | proteasome_(prosome_macropain)_subunit_beta_type_1 |
| TPMT | 2,739192E000 | 0,000000E+001 | 1,017700E-002 | thiopurine_S-methyltransferase |
| BACH1 | 2,738681E000 | 0,000000E+001 | 5,968000E-003 | BTB_and_CNC_homology_1_basic_leucine_zipper_transcription_factor_1 |
| SNAPC5 | 2,734958E000 | 2,000000E-006 | 3,743500E-002 | Small_nuclear_RNA_activating_complex_polypeptide_5_19kDa |
| CLCN5 | 2,727781E000 | 0,000000E+001 | 7,170000E-004 | phosphate_cytidylyltransferase_1_choline_beta_isoform |
| PRKAR2A | 2,724414E000 | 0,000000E+001 | 1,790000E-004 | protein_kinase_cAMP-dependent_regulatory_type_II_alpha |
| PRPF3 | 2,717706E000 | 0,000000E+001 | 9,700000E-005 | PRP3_pre-mRNA_processing_factor_3_homolog_(yeast) |
| MRPS14 | 2,710233E000 | 0,000000E+001 | 1,856000E-003 | mitochondrial_ribosomal_protein_S14 |
| RSF1 | 2,707971E000 | 1,000000E-006 | 2,337800E-002 | Hepatitis_B_virus_x_associated_protein |
| ZNF609 | 2,705148E000 | 1,000000E-006 | 1,188800E-002 | Zinc_finger_protein_609 |
| WDR45 | 2,700627E000 | 0,000000E+001 | 3,000000E-006 | WD_repeat_domain_45 |
| CALD1 | 2,698771E000 | 0,000000E+001 | 6,548000E-003 | caldesmon_1 |
| RAB14 | 2,687894E000 | 1,000000E-006 | 1,899300E-002 | RAB14_member_RAS_oncogene_family |
| SF3B1 | 2,676648E000 | 0,000000E+001 | 1,020000E-004 | splicing_factor_3b_subunit_1_155kDa |
| EIF4G2 | 2,655999E000 | 0,000000E+001 | 1,810000E-004 | eukaryotic_translation_initiation_factor_4_gamma_2 |
| OGFR | 2,651803E000 | 1,000000E-006 | 1,497700E-002 | opioid_growth_factor_receptor |
| PCDHGA3 | 2,651436E000 | 0,000000E+001 | 2,766000E-003 | protocadherin_gamma_subfamily_A_3 |
| HTATSF1 | 2,650240E000 | 0,000000E+001 | 1,820000E-004 | HIV_TAT_specific_factor_1 |
| SFRS15 | 2,645612E000 | 0,000000E+001 | 1,643000E-003 | splicing_factor_arginine/serine-rich_15 |
| HIP1 | 2,639438E000 | 0,000000E+001 | 3,253000E-003 | huntingtin_interacting_protein_1 |
| BAT2D1 | 2,639045E000 | 0,000000E+001 | 1,800000E-005 | BAT2_domain_containing_1 |
| HIC2 | 2,632982E000 | 0,000000E+001 | 2,713000E-003 | hypermethylated_in_cancer_2 |
| HNRPD | 2,628670E000 | 0,000000E+001 | 5,482000E-003 | Heterogeneous_nuclear_ribonucleoprotein_D |
| MLLT10 | 2,621341E000 | 0,000000E+001 | 2,468000E-003 | myeloid/lymphoid_or_mixed-lineage_leukemia_translocated_to_10 |
| JMJD2B | 2,620354E000 | 0,000000E+001 | 3,700000E-004 | jumonji_domain_containing_2B |
| PSPC1 | 2,610785E000 | 0,000000E+001 | 6,979000E-003 | Paraspeckle_component_1 |
| KIAA0256 | 2,606444E000 | 2,000000E-006 | 3,752500E-002 | KIAA0256_gene_product |
| USP34 | 2,605530E000 | 0,000000E+001 | 3,220000E-004 | ubiquitin_specific_protease_34 |
| VTI1B | 2,602774E000 | 0,000000E+001 | 7,693000E-003 | vesicle_transport_through_interaction_with_t-SNAREs_homolog_1B_(yeast) |
| RNF14 | 2,599964E000 | 0,000000E+001 | 2,100000E-005 | ring_finger_protein_14 |
| DGUOK | 2,596572E000 | 0,000000E+001 | 8,960000E-004 | deoxyguanosine_kinase |
| UBC | 2,596355E000 | 0,000000E+001 | 6,700000E-005 | ubiquitin_C |
| COPS6 | 2,590520E000 | 0,000000E+001 | 3,410000E-004 | COP9_constitutive_photomorphogenic_homolog_subunit_6_(Arabidopsis) |
| SON | 2,589138E000 | 0,000000E+001 | 1,550000E-003 | SON_DNA_binding_protein |
| MYO1C | 2,579517E000 | 0,000000E+001 | 7,829000E-003 | myosin_IC |
| HBXIP | 2,576885E000 | 0,000000E+001 | 1,297000E-003 | hepatitis_B_virus_x_interacting_protein |
| OGFR | 2,563673E000 | 0,000000E+001 | 1,508000E-003 | opioid_growth_factor_receptor |
| YES1 | 2,535814E000 | 1,000000E-006 | 1,713900E-002 | V-yes-1_Yamaguchi_sarcoma_viral_oncogene_homolog_1 |
| TMED2 | 2,531135E000 | 0,000000E+001 | 5,400000E-005 | coated_vesicle_membrane_protein |
| XRCC2 | 2,525845E000 | 0,000000E+001 | 3,700000E-005 | X-ray_repair_complementing_defective_repair_in_Chinese_hamster_cells_2 |
| RPL35A | 2,521684E000 | 0,000000E+001 | 1,500000E-005 | Ribosomal_protein_L35a |
| RBM5 | 2,521580E000 | 1,000000E-006 | 1,314300E-002 | RNA_binding_motif_protein_5 |
| ZCCHC2 | 2,503863E000 | 0,000000E+001 | 3,363000E-003 | zinc_finger_CCHC_domain_containing_2 |
| SET | 2,501808E000 | 0,000000E+001 | 5,200000E-005 | SET_translocation_(myeloid_leukemia-associated) |

**higher variances in NC**

| **GeneSymbol** | **variance Fold** | ***P* Brown-Forsythe** | ***P* Bonferroni** | **Full Annotation** |
| --- | --- | --- | --- | --- |
| MSX2 | 2,502477E-000 | 1,000000E-006 | 2,996300E-002 | msh_homeo_box_homolog_2_(Drosophila) |
| HNMT | 2,519516E-000 | 0,000000E+001 | 1,050000E-002 | histamine_N-methyltransferase |
| BOP1 | 2,520090E-000 | 0,000000E+001 | 2,019000E-003 | block_of_proliferation_1 |
| GABARAPL1 | 2,526135E-000 | 1,000000E-006 | 1,679900E-002 | GABA(A)_receptor-associated_protein_like_1 |
| THEM2 | 2,527882E-000 | 0,000000E+001 | 4,385000E-003 | thioesterase_superfamily_member_2 |
| MDH1 | 2,535266E-000 | 1,000000E-006 | 1,454400E-002 | malate_dehydrogenase_1_NAD_(soluble) |
| PRKAG1 | 2,548339E-000 | 0,000000E+001 | 2,410000E-004 | protein_kinase_AMP-activated_gamma_1_non-catalytic_subunit |
| DNAJC10 | 2,548485E-000 | 1,000000E-006 | 3,179900E-002 | DnaJ_(Hsp40)_homolog_subfamily_C_member_10 |
| MTUS1 | 2,565541E-000 | 2,000000E-006 | 3,934200E-002 | mitochondrial_tumor_suppressor_1 |
| VARS | 2,568194E-000 | 1,000000E-006 | 1,954900E-002 | valyl-tRNA_synthetase |
| SMURF1 | 2,573593E-000 | 1,000000E-006 | 1,881800E-002 | SMAD_specific_E3_ubiquitin_protein_ligase_1 |
| DCTN5 | 2,581311E-000 | 2,000000E-006 | 3,766800E-002 | dynactin_4 |
| NLRP3 | 2,585594E-000 | 0,000000E+001 | 3,956000E-003 | cold_autoinflammatory_syndrome_1 |
| NELF | 2,585823E-000 | 0,000000E+001 | 4,832000E-003 | nasal_embryonic_LHRH_factor |
| CES4 | 2,597192E-000 | 0,000000E+001 | 2,940000E-004 | carboxylesterase_4-like |
| COL8A1 | 2,603264E-000 | 0,000000E+001 | 8,449000E-003 | collagen_type_VIII_alpha_1 |
| AKAP2 | 2,603753E-000 | 0,000000E+001 | 1,850000E-004 | A_kinase_(PRKA)_anchor_protein_2 |
| KCNMB1 | 2,650664E-000 | 1,000000E-006 | 1,785000E-002 | potassium_large_conductance_calcium-activated_channel_subfamily_M_beta_member_1 |
| GADD45B | 2,665406E-000 | 0,000000E+001 | 1,383000E-003 | growth_arrest_and_DNA-damage-inducible_beta |
| YTHDF2 | 2,678087E-000 | 0,000000E+001 | 1,818000E-003 | YTH_domain_family_member_2 |
| RPL37A | 2,682515E-000 | 0,000000E+001 | 9,140000E-004 | Ribosomal_protein_L37a |
| DCN | 2,684187E-000 | 0,000000E+001 | 8,522000E-003 | decorin |
| YWHAE | 2,691882E-000 | 0,000000E+001 | 7,537000E-003 | tyrosine_3-monooxygenase/tryptophan_5-monooxygenase_activation_protein_epsilon_polypeptide |
| GPAA1 | 2,692419E-000 | 0,000000E+001 | 1,810000E-004 | GPAA1P_anchor_attachment_protein_1_homolog_(yeast) |
| EXOC7 | 2,713802E-000 | 1,000000E-006 | 2,508700E-002 | exocyst_complex_component_7 |
| POP4 | 2,728356E-000 | 1,000000E-006 | 1,892600E-002 | processing_of_precursor_4_ribonuclease_P/MRP_subunit_(S,_cerevisiae) |
| PEX7 | 2,728505E-000 | 1,000000E-006 | 1,922400E-002 | peroxisomal_biogenesis_factor_7 |
| SCP2 | 2,740206E-000 | 0,000000E+001 | 4,004000E-003 | sterol_carrier_protein_2 |
| ATP8B1 | 2,741856E-000 | 0,000000E+001 | 8,900000E-005 | ATPase_Class_I_type_8B_member_1 |
| MYOG | 2,757474E-000 | 2,000000E-006 | 4,347300E-002 | myogenin_(myogenic_factor_4) |
| EMP1 | 2,772109E-000 | 1,000000E-006 | 2,573100E-002 | epithelial_membrane_protein_1 |
| FAM45B | 2,778898E-000 | 0,000000E+001 | 2,020000E-004 | family_with_sequence_similarity_45_member_B |
| SDCBP | 2,783783E-000 | 1,000000E-006 | 2,600100E-002 | syndecan_binding_protein_(syntenin) |
| HTR5A | 2,790318E-000 | 1,000000E-006 | 1,251400E-002 | 5-hydroxytryptamine_(serotonin)_receptor_5A |
| DYNLRB1 | 2,812745E-000 | 1,000000E-006 | 3,068800E-002 | dynein_cytoplasmic_light_polypeptide_2A |
| SFRS7 | 2,817729E-000 | 1,000000E-006 | 1,821800E-002 | splicing_factor_arginine/serine-rich_7_35kDa |
| RAB4A | 2,822513E-000 | 0,000000E+001 | 2,120000E-004 | RAB4A_member_RAS_oncogene_family |
| SIRPA | 2,848459E-000 | 0,000000E+001 | 9,100000E-005 | protein_tyrosine_phosphatase_non-receptor_type_substrate_1 |
| KIAA0194 | 2,861555E-000 | 0,000000E+001 | 4,770000E-003 | KIAA0194_protein |
| SLC2A3 | 2,868705E-000 | 0,000000E+001 | 3,704000E-003 | solute_carrier_family_2_(facilitated_glucose_transporter)_member_3 |
| MOSPD3 | 2,870808E-000 | 0,000000E+001 | 9,700000E-005 | motile_sperm_domain_containing_3 |
| PTN | 2,891451E-000 | 0,000000E+001 | 1,044000E-002 | pleiotrophin_(heparin_binding_growth_factor_8_neurite_growth-promoting_factor_1) |
| EIF4A1 | 2,893109E-000 | 0,000000E+001 | 2,152000E-003 | Eukaryotic_translation_initiation_factor_4A_isoform_1 |
| SLC7A8 | 2,913872E-000 | 0,000000E+001 | 2,170000E-003 | Solute_carrier_family_7_(cationic_amino_acid_transporter_y+_system)_member_8 |
| TLR7 | 2,917527E-000 | 1,000000E-006 | 1,526900E-002 | toll-like_receptor_7 |
| QDPR | 2,942870E-000 | 1,000000E-006 | 2,186000E-002 | quinoid_dihydropteridine_reductase |
| AKR7A2 | 2,944726E-000 | 1,000000E-006 | 2,470700E-002 | aldo-keto_reductase_family_7_member_A2_(aflatoxin_aldehyde_reductase) |
| UQCRC1 | 2,949190E-000 | 0,000000E+001 | 3,741000E-003 | ubiquinol-cytochrome_c_reductase_core_protein_I |
| PRKACB | 2,953658E-000 | 0,000000E+001 | 9,075000E-003 | protein_kinase_cAMP-dependent_catalytic_beta |
| TTC1 | 2,963417E-000 | 0,000000E+001 | 4,080000E-004 | tetratricopeptide_repeat_domain_1 |
| KIAA1659 | 2,971147E-000 | 1,000000E-006 | 1,539300E-002 | KIAA1659_protein |
| 03/06/07 | 2,974950E-000 | 0,000000E+001 | 7,234000E-003 | membrane-associated_ring_finger_(C3HC4)_6 |
| KLF6 | 2,994541E-000 | 0,000000E+001 | 6,524000E-003 | Kruppel-like_factor_6 |
| YIPF3 | 2,995542E-000 | 0,000000E+001 | 1,521000E-003 | Yip1_domain_family_member_3 |
| EIF5A2 | 3,005013E-000 | 1,000000E-006 | 1,485100E-002 | eukaryotic_translation_initiation_factor_5A2 |
| LMBRD1 | 3,015297E-000 | 0,000000E+001 | 3,040000E-004 | LMBR1_domain_containing_1 |
| TXNL2 | 3,033453E-000 | 0,000000E+001 | 8,400000E-005 | thioredoxin-like_2 |
| SMARCA4 | 3,036689E-000 | 1,000000E-006 | 2,222900E-002 | SWI/SNF_related_matrix_associated_actin_dependent_regulator_of_chromatin_  subfamily_a_member_4 |
| PMS2L1 | 3,042786E-000 | 1,000000E-006 | 2,568100E-002 | postmeiotic_segregation_increased_2-like_1 |
| RBPMS | 3,053729E-000 | 0,000000E+001 | 2,816000E-003 | RNA_binding_protein_with_multiple_splicing |
| EWSR1 | 3,088238E-000 | 0,000000E+001 | 1,019000E-003 | Ewing_sarcoma_breakpoint_region_1 |
| PTHB1 | 3,103051E-000 | 1,000000E-006 | 3,316100E-002 | parathyroid_hormone-responsive_B1_gene |
| MCL1 | 3,116167E-000 | 0,000000E+001 | 2,311000E-003 | Myeloid_cell_leukemia_sequence_1_(BCL2-related) |
| NR4A1 | 3,125415E-000 | 0,000000E+001 | 7,750000E-004 | nuclear_receptor_subfamily_4_group_A_member_1 |
| KTN1 | 3,133944E-000 | 0,000000E+001 | 2,228000E-003 | kinectin_1_(kinesin_receptor) |
| AGA | 3,138458E-000 | 0,000000E+001 | 3,819000E-003 | aspartylglucosaminidase |
| ABHD14A | 3,140587E-000 | 0,000000E+001 | 6,410000E-004 | abhydrolase_domain_containing_14A |
| HNRPDL | 3,152568E-000 | 0,000000E+001 | 2,322000E-003 | heterogeneous_nuclear_ribonucleoprotein_D-like |
| KLF6 | 3,169650E-000 | 0,000000E+001 | 2,029000E-003 | Kruppel-like_factor_6 |
| CD44 | 3,189415E-000 | 0,000000E+001 | 6,100000E-004 | CD44_antigen_(homing_function_and_Indian_blood_group_system) |
| TNFRSF11B | 3,191000E-000 | 1,000000E-006 | 1,243100E-002 | tumor_necrosis_factor_receptor_superfamily_member_11b_(osteoprotegerin) |
| RAD17 | 3,227745E-000 | 0,000000E+001 | 6,742000E-003 | RAD17_homolog_(S,_pombe) |
| BEXL1 | 3,227879E-000 | 2,000000E-006 | 3,594200E-002 | brain_expressed_X-linked-like_1 |
| PGEA1 | 3,229236E-000 | 2,000000E-006 | 3,958300E-002 | PKD2_interactor_golgi_and_endoplasmic_reticulum_associated_1 |
| SLC35E2 | 3,243598E-000 | 1,000000E-006 | 3,065300E-002 | succinate_dehydrogenase_complex_subunit_D_integral_membrane_protein |
| RTN4 | 3,268449E-000 | 1,000000E-006 | 1,332900E-002 | reticulon_4 |
| ADAM17 | 3,269730E-000 | 0,000000E+001 | 3,600000E-005 | a_disintegrin_and_metalloproteinase_domain_17 |
| CRYBA2 | 3,312864E-000 | 1,000000E-006 | 1,492500E-002 | crystallin_beta_A2 |
| NOLC1 | 3,329515E-000 | 0,000000E+001 | 9,010000E-004 | nucleolar_and_coiled-body_phosphoprotein_1 |
| PDGFA | 3,406665E-000 | 1,000000E-006 | 2,064000E-002 | platelet-derived_growth_factor_alpha_polypeptide |
| GADD45B | 3,415281E-000 | 2,000000E-006 | 4,316800E-002 | growth_arrest_and_DNA-damage-inducible_beta |
| SH3BGRL | 3,415544E-000 | 0,000000E+001 | 6,080000E-004 | SH3_domain_binding_glutamic_acid-rich_protein_like |
| SLC25A37 | 3,425844E-000 | 1,000000E-006 | 1,543900E-002 | solute_carrier_family_25_member_37 |
| NCOA3 | 3,432183E-000 | 0,000000E+001 | 5,340000E-004 | nuclear_receptor_coactivator_3 |
| SGCD | 3,435868E-000 | 1,000000E-006 | 2,334800E-002 | sarcoglycan_delta_(35kDa_dystrophin-associated_glycoprotein) |
| EFEMP1 | 3,451377E-000 | 0,000000E+001 | 9,198000E-003 | EGF-containing_fibulin-like_extracellular_matrix_protein_1 |
| DYRK3 | 3,479641E-000 | 2,000000E-006 | 4,344100E-002 | dual-specificity_tyrosine-(Y)-phosphorylation_regulated_kinase_3 |
| MAP7 | 3,482436E-000 | 0,000000E+001 | 5,239000E-003 | microtubule-associated_protein_7 |
| DHPS | 3,491746E-000 | 0,000000E+001 | 8,304000E-003 | deoxyhypusine_synthase |
| HGF | 3,512470E-000 | 0,000000E+001 | 5,170000E-004 | hepatocyte_growth_factor_(hepapoietin_A_scatter_factor) |
| GPAA1 | 3,516894E-000 | 1,000000E-006 | 1,925200E-002 | GPAA1P_anchor_attachment_protein_1_homolog_(yeast) |
| SNX2 | 3,518896E-000 | 0,000000E+001 | 1,090000E-004 | sorting_nexin_2 |
| CEBPB | 3,520939E-000 | 0,000000E+001 | 6,787000E-003 | CCAAT/enhancer_binding_protein_(C/EBP)_beta |
| SIVA1 | 3,522601E-000 | 0,000000E+001 | 4,182000E-003 | CD27-binding_(Siva)_protein |
| C1GALT1C1 | 3,536573E-000 | 0,000000E+001 | 4,710000E-004 | C1GALT1-specific_chaperone_1 |
| ZFP36L1 | 3,566139E-000 | 0,000000E+001 | 1,900000E-005 | zinc_finger_protein_36_C3H_type-like_1 |
| HOXC4 | 3,574695E-000 | 0,000000E+001 | 3,170000E-004 | homeo_box_C4 |
| DYRK4 | 3,578640E-000 | 0,000000E+001 | 8,530000E-004 | dual-specificity_tyrosine-(Y)-phosphorylation_regulated_kinase_4 |
| NVL | 3,587230E-000 | 0,000000E+001 | 1,970000E-004 | nuclear_VCP-like |
| PSCD1 | 3,617171E-000 | 0,000000E+001 | 7,100000E-005 | pleckstrin_homology_Sec7_and_coiled-coil_domains_1(cytohesin_1) |
| NAGLU | 3,621575E-000 | 0,000000E+001 | 5,780000E-004 | N-acetylglucosaminidase_alpha-_(Sanfilippo_disease_IIIB) |
| LRIG2 | 3,638985E-000 | 2,000000E-006 | 4,021900E-002 | leucine-rich_repeats_and_immunoglobulin-like_domains_2 |
| PSCD3 | 3,639280E-000 | 0,000000E+001 | 2,508000E-003 | pleckstrin_homology_Sec7_and_coiled-coil_domains_3 |
| SH3YL1 | 3,642128E-000 | 1,000000E-006 | 2,862200E-002 | SH3_domain_containing_Ysc84-like_1_(S,_cerevisiae) |
| B4GALT1 | 3,647523E-000 | 0,000000E+001 | 4,205000E-003 | UDP-Gal:betaGlcNAc_beta_14-_galactosyltransferase_polypeptide_1 |
| CHD1 | 3,693983E-000 | 0,000000E+001 | 5,461000E-003 | chromodomain_helicase_DNA_binding_protein_1 |
| JMJD2B | 3,718577E-000 | 0,000000E+001 | 1,072800E-002 | Jumonji_domain_containing_2B |
| TNC | 3,728759E-000 | 2,000000E-006 | 3,763700E-002 | Tenascin_C_(hexabrachion) |
| SNF1LK | 3,743665E-000 | 0,000000E+001 | 3,900000E-004 | SNF1-like_kinase |
| FZD1 | 3,794122E-000 | 1,000000E-006 | 2,094100E-002 | frizzled_homolog_1_(Drosophila) |
| JUN | 3,797559E-000 | 0,000000E+001 | 3,760000E-004 | v-jun_sarcoma_virus_17_oncogene_homolog_(avian) |
| CAPZA1 | 3,814089E-000 | 2,000000E-006 | 4,956300E-002 | capping_protein_(actin_filament)_muscle_Z-line_alpha_1 |
| NFKBIA | 3,862618E-000 | 0,000000E+001 | 9,680000E-004 | nuclear_factor_of_kappa_light_polypeptide_gene_enhancer_in_B-cells_inhibitor_alpha |
| ZDHHC4 | 3,872313E-000 | 0,000000E+001 | 1,900000E-004 | zinc_finger_DHHC-type_containing_4 |
| RUTBC3 | 3,933754E-000 | 0,000000E+001 | 4,700000E-004 | RUN_and_TBC1_domain_containing_3 |
| VEGFA | 4,138512E-000 | 1,000000E-006 | 1,571700E-002 | vascular_endothelial_growth_factor |
| ATIC | 4,139176E-000 | 0,000000E+001 | 9,650000E-004 | 5-aminoimidazole-4-carboxamide_ribonucleotide_formyltransferase/IMP_cyclohydrolase |
| CTSO | 4,146943E-000 | 0,000000E+001 | 2,921000E-003 | cathepsin_O |
| PPP1R3C | 4,173486E-000 | 0,000000E+001 | 6,040000E-004 | protein_phosphatase_1_regulatory_(inhibitor)_subunit_3C |
| RNH1 | 4,176323E-000 | 0,000000E+001 | 2,400000E-003 | ribonuclease/angiogenin_inhibitor_1 |
| PDE4DIP | 4,200765E-000 | 1,000000E-006 | 3,205500E-002 | phosphodiesterase_4D_interacting_protein_(myomegalin) |
| RALBP1 | 4,204992E-000 | 0,000000E+001 | 8,840000E-004 | ralA_binding_protein_1 |
| RBKS | 4,232032E-000 | 0,000000E+001 | 3,609000E-003 | ribokinase |
| ZFP36 | 4,372285E-000 | 0,000000E+001 | 2,044000E-003 | zinc_finger_protein_36_C3H_type_homolog_(mouse) |
| LTA4H | 4,392082E-000 | 0,000000E+001 | 5,644000E-003 | leukotriene_A4_hydrolase |
| PSG2 | 4,395308E-000 | 0,000000E+001 | 6,872000E-003 | pregnancy_specific_beta-1-glycoprotein_2 |
| ATP6V0D1 | 4,418779E-000 | 0,000000E+001 | 2,668000E-003 | ATPase_H+_transporting_lysosomal_38kDa_V0_subunit_d_isoform_1 |
| GMPR2 | 4,463440E-000 | 2,000000E-006 | 4,264100E-002 | guanosine_monophosphate_reductase_2 |
| VEGFA | 4,512158E-000 | 1,000000E-006 | 1,872300E-002 | vascular_endothelial_growth_factor |
| DNAJB12 | 4,556978E-000 | 0,000000E+001 | 2,270000E-003 | DnaJ_(Hsp40)_homolog_subfamily_B_member_12 |
| SAMM50 | 4,576245E-000 | 0,000000E+001 | 1,600000E-005 | CGI-51_protein |
| MCL1 | 4,582163E-000 | 0,000000E+001 | 3,100000E-005 | myeloid_cell_leukemia_sequence_1_(BCL2-related) |
| SEMA4G | 4,670205E-000 | 0,000000E+001 | 9,530000E-004 | sema_domain_immunoglobulin_domain_transmembrane_domain_and_short_cytoplasmic_domain |
| GALNT11 | 4,713194E-000 | 0,000000E+001 | 4,734000E-003 | UDP-N-acetyl-alpha-D-galactosamine: polypeptide_N-acetylgalactosaminyltransferase_11 |
| BAI1 | 4,727356E-000 | 1,000000E-006 | 1,570300E-002 | brain-specific_angiogenesis_inhibitor_1 |
| SNAPC3 | 4,763168E-000 | 0,000000E+001 | 1,087000E-003 | small_nuclear_RNA_activating_complex_polypeptide_3_50kDa |
| TFAM | 4,780353E-000 | 0,000000E+001 | 3,450000E-004 | transcription_factor_A_mitochondrial |
| WDR43 | 4,784807E-000 | 2,000000E-006 | 4,443000E-002 | WD_repeat_domain_43 |
| CLN5 | 4,785069E-000 | 0,000000E+001 | 8,980000E-004 | ceroid-lipofuscinosis_neuronal_5 |
| KDELR3 | 4,920132E-000 | 0,000000E+001 | 6,698000E-003 | KDEL_(Lys-Asp-Glu-Leu)_endoplasmic_reticulum_protein_retention_receptor_3 |
| SARM1 | 5,032046E-000 | 0,000000E+001 | 6,428000E-003 | sterile_alpha_and_TIR_motif_containing_1 |
| LRRC3 | 5,115611E-000 | 0,000000E+001 | 4,200000E-005 | leucine_rich_repeat_containing_3 |
| HGF | 5,122335E-000 | 1,000000E-006 | 2,932400E-002 | hepatocyte_growth_factor_(hepapoietin_A_scatter_factor) |
| PCDH17 | 5,151360E-000 | 2,000000E-006 | 3,906100E-002 | protocadherin_17 |
| ADAM7 | 5,158442E-000 | 0,000000E+001 | 9,640000E-004 | a_disintegrin_and_metalloproteinase_domain_7 |
| SLC19A1 | 5,168576E-000 | 0,000000E+001 | 4,719000E-003 | solute_carrier_family_19_(folate_transporter)_member_1 |
| STAT1 | 5,169511E-000 | 0,000000E+001 | 1,510000E-003 | signal_transducer_and_activator_of_transcription_1_91kDa |
| OTUB2 | 5,176807E-000 | 1,000000E-006 | 1,555100E-002 | OTU_domain_ubiquitin_aldehyde_binding_2 |
| SOCS3 | 5,242544E-000 | 0,000000E+001 | 5,660000E-004 | suppressor_of_cytokine_signaling_3 |
| TNFAIP3 | 5,252440E-000 | 1,000000E-006 | 2,385300E-002 | tumor_necrosis_factor_alpha-induced_protein_3 |
| DKK2 | 5,253374E-000 | 2,000000E-006 | 4,383000E-002 | dickkopf_homolog_2_(Xenopus_laevis) |
| DLD | 5,327441E-000 | 0,000000E+001 | 1,028000E-003 | dihydrolipoamide_dehydrogenase |
| BRF2 | 5,361736E-000 | 1,000000E-006 | 1,273000E-002 | BRF2_subunit_of_RNA_polymerase_III_transcription_initiation_factor_BRF1-like |
| MYC | 5,438417E-000 | 0,000000E+001 | 6,649000E-003 | v-myc_myelocytomatosis_viral_oncogene_homolog_(avian) |
| UBL4A | 5,508538E-000 | 0,000000E+001 | 1,075400E-002 | ubiquitin-like_4 |
| UIMC1 | 5,511773E-000 | 1,000000E-006 | 1,794800E-002 | receptor_associated_protein_80 |
| SERBP1 | 5,566705E-000 | 0,000000E+001 | 5,338000E-003 | PAI-1_mRNA_binding_protein |
| COL1A1 | 5,583288E-000 | 1,000000E-006 | 1,708800E-002 | collagen_type_I_alpha_1 |
| DAZAP1 | 5,602140E-000 | 0,000000E+001 | 8,740000E-004 | DAZ_associated_protein_1 |
| FECH | 5,832463E-000 | 1,000000E-006 | 1,156900E-002 | ferrochelatase_(protoporphyria) |
| SLC29A3 | 6,141542E-000 | 1,000000E-006 | 1,862800E-002 | solute_carrier_family_29_(nucleoside_transporters)_member_3 |
| SET | 6,143979E-000 | 0,000000E+001 | 1,572000E-003 | SET_translocation_(myeloid_leukemia-associated) |
| CBX8 | 6,178273E-000 | 1,000000E-006 | 1,390700E-002 | chromobox_homolog_8_(Pc_class_homolog_Drosophila) |
| ITFG1 | 6,311262E-000 | 1,000000E-006 | 1,700300E-002 | T-cell_immunomodulatory_protein |
| TCEB1 | 6,342607E-000 | 0,000000E+001 | 1,667000E-003 | transcription_elongation_factor_B_(SIII)_polypeptide_1_(15kDa_elongin_C) |
| NR4A1 | 6,373088E-000 | 0,000000E+001 | 9,210000E-003 | nuclear_receptor_subfamily_4_group_A_member_1 |
| NMUR1 | 6,393422E-000 | 0,000000E+001 | 2,146000E-003 | neuromedin_U_receptor_1 |
| DUSP5 | 6,568973E-000 | 0,000000E+001 | 3,309000E-003 | dual_specificity_phosphatase_5 |
| ARL6IP5 | 6,575361E-000 | 0,000000E+001 | 4,270000E-003 | ADP-ribosylation-like_factor_6_interacting_protein_5 |
| PPP2R1B | 6,638418E-000 | 2,000000E-006 | 4,602700E-002 | protein_phosphatase_2_(formerly_2A)_regulatory_subunit_A_(PR_65)_beta_isoform |
| ATP6V1C1 | 6,642715E-000 | 0,000000E+001 | 1,150000E-004 | ATPase_H+_transporting_lysosomal_42kDa_V1_subunit_C_isoform_1 |
| CHRNB1 | 6,725979E-000 | 1,000000E-006 | 2,484400E-002 | cholinergic_receptor_nicotinic_beta_polypeptide_1_(muscle) |
| HSP90AA1 | 6,744053E-000 | 1,000000E-006 | 1,577100E-002 | heat_shock_90kDa_protein_1_alpha |
| FAM89B | 6,790798E-000 | 2,000000E-006 | 4,601400E-002 | Mouse_Mammary_Turmor_Virus_Receptor_homolog_1 |
| PIGT | 6,900686E-000 | 2,000000E-006 | 4,896300E-002 | phosphatidylinositol_glycan_class_T |
| PEPD | 7,143564E-000 | 1,000000E-006 | 1,481400E-002 | peptidase_D |
| TARBP1 | 7,145698E-000 | 0,000000E+001 | 3,377000E-003 | TAR_(HIV)_RNA_binding_protein_1 |
| MCL1 | 7,270091E-000 | 0,000000E+001 | 9,674000E-003 | myeloid_cell_leukemia_sequence_1_(BCL2-related) |
| SET | 7,409800E-000 | 0,000000E+001 | 9,070000E-003 | SET_translocation_(myeloid_leukemia-associated) |
| NFIL3 | 7,521108E-000 | 1,000000E-006 | 1,379200E-002 | nuclear_factor_interleukin_3_regulated |
| KPNB1 | 7,568118E-000 | 2,000000E-006 | 3,396900E-002 | karyopherin_(importin)_beta_1 |
| KLHDC2 | 7,689869E-000 | 2,000000E-006 | 3,710000E-002 | kelch_domain_containing_2 |
| TIPARP | 7,718418E-000 | 1,000000E-006 | 1,177000E-002 | TCDD-inducible_poly(ADP-ribose)_polymerase |
| CDKN1A | 7,720190E-000 | 1,000000E-006 | 1,292000E-002 | cyclin-dependent_kinase_inhibitor_1A_(p21_Cip1) |
| GPAA1 | 7,742810E-000 | 0,000000E+001 | 1,085000E-003 | GPAA1P_anchor_attachment_protein_1_homolog_(yeast) |
| XRCC4 | 7,748119E-000 | 2,000000E-006 | 4,314600E-002 | X-ray_repair_complementing_defective_repair_in_Chinese_hamster_cells_4 |
| CCNL1 | 7,785239E-000 | 0,000000E+001 | 7,090000E-003 | cyclin_L1 |
| RNF187 | 7,848948E-000 | 0,000000E+001 | 5,089000E-003 | ring_finger_protein_187 |
| IL1R1 | 7,856095E-000 | 0,000000E+001 | 4,227000E-003 | interleukin_1_receptor_type_I |
| PPP1R15A | 7,919468E-000 | 0,000000E+001 | 7,712000E-003 | protein_phosphatase_1_regulatory_(inhibitor)_subunit_15A |
| CORO1B | 7,919643E-000 | 0,000000E+001 | 5,734000E-003 | coronin_actin_binding_protein_1B |
| ZIC1 | 7,945457E-000 | 0,000000E+001 | 1,110000E-004 | Zic_family_member_1_(odd-paired_homolog_Drosophila) |
| DNAJB9 | 7,968793E-000 | 0,000000E+001 | 1,570000E-004 | DnaJ_(Hsp40)_homolog_subfamily_B_member_9 |
| RPA1 | 8,008671E-000 | 1,000000E-006 | 2,628400E-002 | replication_protein_A1_70kDa |
| GLT8D1 | 8,010412E-000 | 1,000000E-006 | 2,152100E-002 | glycosyltransferase_8_domain_containing_1 |
| PTDSR | 8,056482E-000 | 1,000000E-006 | 1,532900E-002 | phosphatidylserine_receptor |
| WNT5B | 8,070905E-000 | 2,000000E-006 | 3,370300E-002 | wingless-type_MMTV_integration_site_family_member_5B |
| ECD | 8,489739E-000 | 0,000000E+001 | 7,377000E-003 | suppressor_of_S,_cerevisiae_gcr2 |
| KCNK15 | 8,499686E-000 | 0,000000E+001 | 1,208000E-003 | potassium_channel_subfamily_K_member_15 |
| NR4A3 | 8,589671E-000 | 0,000000E+001 | 2,030000E-004 | nuclear_receptor_subfamily_4_group_A_member_3 |
| DRG1 | 8,737960E-000 | 0,000000E+001 | 3,419000E-003 | developmentally_regulated_GTP_binding_protein_1 |
| SLC1A3 | 8,748515E-000 | 1,000000E-006 | 1,703900E-002 | solute_carrier_family_1_(glial_high_affinity_glutamate_transporter)_member_3 |
| RBMS1 | 8,980822E-000 | 0,000000E+001 | 5,275000E-003 | RNA_binding_motif_single_stranded_interacting_protein_1 |
| ITGB1 | 9,127588E-000 | 0,000000E+001 | 8,110000E-004 | integrin_beta_1 |
| N4BP1 | 9,218854E-000 | 2,000000E-006 | 3,926300E-002 | Nedd4_binding_protein_1 |
| HDAC11 | 9,280802E-000 | 0,000000E+001 | 6,896000E-003 | histone_deacetylase_11 |
| INSIG2 | 9,636388E-000 | 1,000000E-006 | 2,626200E-002 | insulin_induced_gene_2 |
| SLC2A3 | 9,758167E-000 | 0,000000E+001 | 7,195000E-003 | solute_carrier_family_2_(facilitated_glucose_transporter)_member_3 |
| JUND | 1,008212E+001 | 0,000000E+001 | 7,100000E-005 | jun_D_proto-oncogene |
| NUFIP1 | 1,057967E+001 | 0,000000E+001 | 1,527000E-003 | nuclear_fragile_X_mental_retardation_protein_interacting_protein_1 |
| MAT2A | 1,084030E+001 | 0,000000E+001 | 3,810000E-004 | methionine_adenosyltransferase_II_alpha |
| CSNK1A1 | 1,158017E+001 | 2,000000E-006 | 4,317100E-002 | casein_kinase_1_alpha_1 |
| LOH11CR2A | 1,170930E+001 | 1,000000E-006 | 2,716600E-002 | loss_of_heterozygosity_11_chromosomal_region_2_gene_A |
| ALAS1 | 1,190819E+001 | 0,000000E+001 | 4,456000E-003 | aminolevulinate_delta-_synthase_1 |
| TM6SF1 | 1,204094E+001 | 0,000000E+001 | 3,370000E-004 | transmembrane_6_superfamily_member_1 |
| ITGB2 | 1,259112E+001 | 2,000000E-006 | 4,590400E-002 | integrin_beta_2_lymphocyte_function-associated_antigen_1_macrophage_antigen_1 |
| ATF3 | 1,290380E+001 | 1,000000E-006 | 1,272000E-002 | activating_transcription_factor_3 |
| RHOB | 1,301717E+001 | 1,000000E-006 | 2,487600E-002 | ras_homolog_gene_family_member_B |
| GADD45B | 1,340521E+001 | 0,000000E+001 | 3,210000E-004 | growth_arrest_and_DNA-damage-inducible_beta |
| CXCL2 | 1,373533E+001 | 1,000000E-006 | 1,714800E-002 | chemokine_(C-X-C_motif)_ligand_2 |
| HNRPH3 | 1,405303E+001 | 0,000000E+001 | 1,508000E-003 | heterogeneous_nuclear_ribonucleoprotein_H3_(2H9) |
| PCNXL2 | 1,478092E+001 | 0,000000E+001 | 3,716000E-003 | pecanex-like_2_(Drosophila) |
| TNFAIP3 | 1,622234E+001 | 0,000000E+001 | 2,510000E-004 | tumor_necrosis_factor_alpha-induced_protein_3 |
| GAPDH | 1,649720E+001 | 2,000000E-006 | 5,078600E-002 | glyceraldehyde-3-phosphate_dehydrogenase |
| NIT1 | 2,160073E+001 | 1,000000E-006 | 2,751500E-002 | nitrilase_1 |
| VEGFA | 2,216975E+001 | 0,000000E+001 | 6,393000E-003 | vascular_endothelial_growth_factor |
| TXNL4A | 6,015448E+001 | 1,000000E-006 | 1,642200E-002 | thioredoxin-like_4A |
| NOL7 | 6,048953E+001 | 1,000000E-006 | 1,882900E-002 | nucleolar_protein_7_27kDa |

# Supplementary Table 1C: Genes affected by intra-group, inter-individual mRNA expression variances (RA compared to OA):

**higher variances in RA**

| **GeneSymbol** | **variance Fold** | ***P* Brown-Forsythe** | ***P* Bonferroni** | **Full Annotation** |
| --- | --- | --- | --- | --- |
| PLA2G2D | 6,469390E+001 | 0,000000E+001 | 4,851000E-003 | phospholipase_A2_group_IID |
| KCNA3 | 1,969289E+001 | 1,000000E-006 | 2,957800E-002 | potassium_voltage-gated_channel_shaker-related_subfamily_member_3 |
| KCNN3 | 1,779663E+001 | 0,000000E+001 | 6,860000E-004 | potassium_intermediate/small_conductance_calcium-activated_channel_subfamily_N_member_3 |
| THBS4 | 1,692421E+001 | 1,000000E-006 | 2,426800E-002 | thrombospondin_4 |
| TCEB2 | 1,518529E+001 | 0,000000E+001 | 4,900000E-004 | transcription_elongation_factor_B_(SIII)_polypeptide_2_(18kDa_elongin_B) |
| HLA-DRB6 | 1,311316E+001 | 2,000000E-006 | 4,296300E-002 | major_histocompatibility_complex_class_II_DR_beta_6_(pseudogene) |
| PITX1 | 1,250636E+001 | 0,000000E+001 | 1,900000E-005 | paired-like_homeodomain_transcription_factor_1 |
| WARS | 1,134000E+001 | 0,000000E+001 | 4,830000E-004 | tryptophanyl-tRNA_synthetase |
| KCNN3 | 1,070325E+001 | 0,000000E+001 | 8,170000E-004 | potassium_intermediate/small_conductance_calcium-activated_channel_subfamily_N_member_3 |
| TNFAIP3 | 9,802763E000 | 0,000000E+001 | 1,000000E-006 | tumor_necrosis_factor_alpha-induced_protein_3 |
| CRHR1 | 9,800446E000 | 0,000000E+001 | 9,645000E-003 | corticotropin_releasing_hormone_receptor_1 |
| TNRC9 | 9,666757E000 | 0,000000E+001 | 2,320000E-004 | trinucleotide_repeat_containing_9 |
| CSF2RB | 9,204178E000 | 0,000000E+001 | 2,000000E-005 | colony_stimulating_factor_2_receptor_beta_low-affinity_(granulocyte-macrophage) |
| WISP2 | 8,826098E000 | 1,000000E-006 | 2,535300E-002 | WNT1_inducible_signaling_pathway_protein_2 |
| WNT8B | 8,606908E000 | 1,000000E-006 | 2,239300E-002 | DEAD_(Asp-Glu-Ala-Asp)_box_polypeptide_18 |
| LAMP3 | 8,434842E000 | 1,000000E-006 | 1,190800E-002 | lysosomal-associated_membrane_protein_3 |
| ZIC1 | 8,296404E000 | 0,000000E+001 | 1,000000E-006 | Zic_family_member_1_(odd-paired_homolog_Drosophila) |
| HLA-DRB4 | 8,227225E000 | 0,000000E+001 | 1,590000E-004 | major_histocompatibility_complex_class_II_DR_beta_4 |
| NHP2L1 | 7,822835E000 | 0,000000E+001 | 7,906000E-003 | NHP2_non-histone_chromosome_protein_2-like_1_(S,_cerevisiae) |
| ADAM7 | 7,813913E000 | 0,000000E+001 | 2,600000E-004 | a_disintegrin_and_metalloproteinase_domain_7 |
| CLU | 7,331983E000 | 0,000000E+001 | 1,800000E-004 | clusterin |
| APOM | 7,175426E000 | 2,000000E-006 | 4,322800E-002 | apolipoprotein_M |
| IRF1 | 6,811754E000 | 0,000000E+001 | 3,202000E-003 | interferon_regulatory_factor_1 |
| CYLD | 6,754202E000 | 0,000000E+001 | 1,249000E-003 | cylindromatosis_(turban_tumor_syndrome) |
| NCAPH | 6,724059E000 | 1,000000E-006 | 1,281700E-002 | barren_homolog_(Drosophila) |
| MALT1 | 6,558528E000 | 0,000000E+001 | 9,000000E-006 | mucosa_associated_lymphoid_tissue_lymphoma_translocation_gene_1 |
| TM6SF1 | 6,472332E000 | 0,000000E+001 | 1,740000E-004 | transmembrane_6_superfamily_member_1 |
| GPRC5C | 6,388840E000 | 1,000000E-006 | 1,710300E-002 | G_protein-coupled_receptor_family_C_group_5_member_C |
| CIRBP | 6,287515E000 | 0,000000E+001 | 1,930000E-004 | cold_inducible_RNA_binding_protein |
| KAZALD1 | 6,163151E000 | 0,000000E+001 | 3,100000E-005 | Kazal-type_serine_protease_inhibitor_domain_1 |
| LTC4S | 6,148185E000 | 0,000000E+001 | 1,044000E-003 | leukotriene_C4_synthase |
| SULT1E1 | 6,119007E000 | 0,000000E+001 | 4,200000E-005 | sulfotransferase_family_1E_estrogen-preferring_member_1 |
| GAS1 | 5,994534E000 | 0,000000E+001 | 6,370000E-004 | growth_arrest-specific_1 |
| SNN | 5,957509E000 | 0,000000E+001 | 2,600000E-005 | stannin |
| HOXC10 | 5,886958E000 | 0,000000E+001 | 9,110000E-004 | homeo_box_C10 |
| ADAMDEC1 | 5,860312E000 | 0,000000E+001 | 9,600000E-005 | ADAM-like_decysin_1 |
| IRF4 | 5,818071E000 | 0,000000E+001 | 4,980000E-004 | interferon_regulatory_factor_4 |
| WARS | 5,809654E000 | 0,000000E+001 | 6,120000E-004 | tryptophanyl-tRNA_synthetase |
| PKNOX2 | 5,545551E000 | 1,000000E-006 | 2,474900E-002 | PBX/knotted_1_homeobox_2 |
| EFEMP1 | 5,499365E000 | 0,000000E+001 | 4,675000E-003 | EGF-containing_fibulin-like_extracellular_matrix_protein_1 |
| CXCL9 | 5,430652E000 | 0,000000E+001 | 0,000000E+001 | chemokine_(C-X-C_motif)_ligand_9 |
| SLC39A8 | 5,424737E000 | 0,000000E+001 | 4,380000E-004 | solute_carrier_family_39_(zinc_transporter)_member_8 |
| NBN | 5,352402E000 | 0,000000E+001 | 1,000000E-006 | nibrin |
| MLF1 | 5,228450E000 | 0,000000E+001 | 1,900000E-005 | myeloid_leukemia_factor_1 |
| PPP1CB | 5,202059E000 | 0,000000E+001 | 1,670000E-004 | protein_phosphatase_1_catalytic_subunit_beta_isoform |
| HHLA3 | 5,135812E000 | 0,000000E+001 | 2,250000E-003 | HERV-H_LTR-associating_3 |
| MFAP4 | 5,099129E000 | 0,000000E+001 | 3,940000E-004 | microfibrillar-associated_protein_4 |
| KCNK15 | 5,066389E000 | 1,000000E-006 | 2,502600E-002 | potassium_channel_subfamily_K_member_15 |
| NR2F1 | 5,044845E000 | 0,000000E+001 | 5,537000E-003 | Nuclear_receptor_subfamily_2_group_F_member_1 |
| MREG | 5,011391E000 | 0,000000E+001 | 3,613000E-003 | dilute_suppressor |
| CDCA8 | 4,999726E000 | 2,000000E-006 | 5,085700E-002 | cell_division_cycle_associated_8 |
| PKNOX2 | 4,927285E000 | 1,000000E-006 | 1,731100E-002 | PBX/knotted_1_homeobox_2 |
| HOXA9 | 4,835148E000 | 0,000000E+001 | 7,209000E-003 | homeo_box_A9 |
| MALT1 | 4,798284E000 | 0,000000E+001 | 1,381000E-003 | mucosa_associated_lymphoid_tissue_lymphoma_translocation_gene_1 |
| TPD52L1 | 4,785529E000 | 0,000000E+001 | 1,000000E-006 | tumor_protein_D52-like_1 |
| PCK1 | 4,692797E000 | 0,000000E+001 | 1,404000E-003 | phosphoenolpyruvate_carboxykinase_1_(soluble) |
| ABCA8 | 4,631549E000 | 2,000000E-006 | 4,542800E-002 | ATP-binding_cassette_sub-family_A_(ABC1)_member_8 |
| PITX1 | 4,607349E000 | 0,000000E+001 | 1,100000E-005 | paired-like_homeodomain_transcription_factor_1 |
| TNFAIP8 | 4,567634E000 | 0,000000E+001 | 7,965000E-003 | tumor_necrosis_factor_alpha-induced_protein_8 |
| B4GALT1 | 4,559093E000 | 1,000000E-006 | 1,162000E-002 | UDP-Gal:betaGlcNAc_beta_14-_galactosyltransferase_polypeptide_1 |
| PIK3CD | 4,472450E000 | 0,000000E+001 | 7,372000E-003 | phosphoinositide-3-kinase_catalytic_delta_polypeptide |
| NFE2L3 | 4,460414E000 | 0,000000E+001 | 1,412000E-003 | nuclear_factor_(erythroid-derived_2)-like_3 |
| IL7R | 4,443542E000 | 0,000000E+001 | 1,086000E-003 | interleukin_7_receptor |
| TIMM44 | 4,422835E000 | 0,000000E+001 | 4,548000E-003 | translocase_of_inner_mitochondrial_membrane_44_homolog_(yeast) |
| TNIP2 | 4,395087E000 | 0,000000E+001 | 1,392000E-003 | TNFAIP3_interacting_protein_2 |
| DHRS9 | 4,354905E000 | 0,000000E+001 | 7,140000E-003 | dehydrogenase/reductase_(SDR_family)_member_9 |
| GSTM4 | 4,289118E000 | 2,000000E-006 | 3,747700E-002 | glutathione_S-transferase_M4 |
| SIX1 | 4,285978E000 | 0,000000E+001 | 5,500000E-005 | sine_oculis_homeobox_homolog_1_(Drosophila) |
| CLU | 4,121558E000 | 0,000000E+001 | 2,084000E-003 | clusterin |
| MGAT4C | 4,109870E000 | 0,000000E+001 | 4,761000E-003 | UDP-N-acetylglucosamine:a-13-D-mannoside_beta-14-N-acetylglucosaminyltransferase_IV |
| ANXA9 | 4,090696E000 | 1,000000E-006 | 2,915600E-002 | annexin_A9 |
| SH3YL1 | 4,022770E000 | 1,000000E-006 | 3,156400E-002 | SH3_domain_containing_Ysc84-like_1_(S,_cerevisiae) |
| UBD | 3,980045E000 | 0,000000E+001 | 5,000000E-006 | ubiquitin_D |
| IL1RN | 3,929133E000 | 1,000000E-006 | 2,622800E-002 | interleukin_1_receptor_antagonist |
| THTPA | 3,905474E000 | 0,000000E+001 | 1,106000E-003 | thiamine_triphosphatase |
| ATF5 | 3,897849E000 | 1,000000E-006 | 2,286500E-002 | activating_transcription_factor_5 |
| HGF | 3,892156E000 | 0,000000E+001 | 4,669000E-003 | hepatocyte_growth_factor_(hepapoietin_A_scatter_factor) |
| POLD2 | 3,831774E000 | 0,000000E+001 | 3,257000E-003 | polymerase_(DNA_directed)_delta_2_regulatory_subunit_50kDa |
| ITGBL1 | 3,827589E000 | 0,000000E+001 | 2,621000E-003 | integrin_beta-like_1_(with_EGF-like_repeat_domains) |
| MCL1 | 3,818610E000 | 1,000000E-006 | 1,774100E-002 | myeloid_cell_leukemia_sequence_1_(BCL2-related) |
| PNPLA4 | 3,812501E000 | 0,000000E+001 | 8,640000E-004 | patatin-like_phospholipase_domain_containing_4 |
| CCT6B | 3,788596E000 | 2,000000E-006 | 5,067900E-002 | chaperonin_containing_TCP1_subunit_6B_(zeta_2) |
| SSBP2 | 3,785083E000 | 1,000000E-006 | 2,935200E-002 | single-stranded_DNA_binding_protein_2 |
| TMED3 | 3,778502E000 | 1,000000E-006 | 2,205000E-002 | transmembrane_emp24_domain_containing_3 |
| SMAD3 | 3,773900E000 | 0,000000E+001 | 8,514000E-003 | SMAD_mothers_against_DPP_homolog_3_(Drosophila) |
| MOXD1 | 3,730071E000 | 0,000000E+001 | 2,731000E-003 | monooxygenase_DBH-like_1 |
| SLC22A4 | 3,722007E000 | 1,000000E-006 | 2,681500E-002 | solute_carrier_family_22_(organic_cation_transporter)_member_4 |
| ARPC1A | 3,718605E000 | 0,000000E+001 | 2,516000E-003 | actin_related_protein_2/3_complex_subunit_1A_41kDa |
| LCP1 | 3,697149E000 | 0,000000E+001 | 7,820000E-004 | lymphocyte_cytosolic_protein_1_(L-plastin) |
| PPP1R3C | 3,663246E000 | 0,000000E+001 | 5,000000E-005 | protein_phosphatase_1_regulatory_(inhibitor)_subunit_3C |
| TAP1 | 3,649515E000 | 0,000000E+001 | 2,000000E-006 | transporter_1_ATP-binding_cassette_sub-family_B_(MDR/TAP) |
| TCERG1 | 3,647998E000 | 0,000000E+001 | 7,718000E-003 | transcription_elongation_regulator_1 |
| ALDH1A1 | 3,618221E000 | 0,000000E+001 | 2,152000E-003 | aldehyde_dehydrogenase_1_family_member_A1 |
| PLA2G7 | 3,580498E000 | 0,000000E+001 | 1,083100E-002 | phospholipase_A2_group_VII_(platelet-activating_factor_acetylhydrolase_plasma) |
| SLC2A3 | 3,538705E000 | 0,000000E+001 | 2,100000E-005 | solute_carrier_family_2_(facilitated_glucose_transporter)_member_3 |
| RBKS | 3,519675E000 | 0,000000E+001 | 1,000000E-005 | ribokinase |
| FNDC4 | 3,503549E000 | 0,000000E+001 | 5,000000E-006 | fibronectin_type_III_domain_containing_4 |
| GBP1 | 3,502488E000 | 0,000000E+001 | 1,300000E-005 | guanylate_binding_protein_1_interferon-inducible_67kDa |
| POLR3C | 3,466741E000 | 0,000000E+001 | 3,190000E-004 | polymerase_(RNA)_III_(DNA_directed)_polypeptide_C_(62kD) |
| PRR3 | 3,466042E000 | 2,000000E-006 | 4,388000E-002 | proline_rich_3 |
| LRP1B | 3,461648E000 | 0,000000E+001 | 3,610000E-004 | low_density_lipoprotein-related_protein_1B_(deleted_in_tumors) |
| IGHD | 3,452122E000 | 1,000000E-006 | 3,211900E-002 | immunoglobulin_heavy_constant_delta |
| RASA4 | 3,442493E000 | 0,000000E+001 | 1,300000E-005 | RAS_p21_protein_activator_4 |
| ADCY2 | 3,437561E000 | 0,000000E+001 | 3,980000E-004 | adenylate_cyclase_2_(brain) |
| PTP4A3 | 3,424140E000 | 0,000000E+001 | 5,970000E-004 | protein_tyrosine_phosphatase_type_IVA_member_3 |
| MTMR1 | 3,390046E000 | 0,000000E+001 | 6,526000E-003 | myotubularin_related_protein_1 |
| FAM26B | 3,337786E000 | 0,000000E+001 | 2,445000E-003 | family_with_sequence_similarity_26_member_B |
| ZDHHC18 | 3,299067E000 | 0,000000E+001 | 3,677000E-003 | zinc_finger_DHHC-type_containing_18 |
| TNNI3K | 3,295576E000 | 0,000000E+001 | 2,011000E-003 | TNNI3_interacting_kinase |
| PAM | 3,290895E000 | 0,000000E+001 | 1,068000E-003 | peptidylglycine_alpha-amidating_monooxygenase |
| GALNT11 | 3,282993E000 | 0,000000E+001 | 5,090000E-004 | UDP-N-acetyl-alpha-D-galactosamine:polypeptide_N-acetylgalactosaminyltransferase_11 |
| PGCP | 3,281650E000 | 1,000000E-006 | 1,584200E-002 | plasma_glutamate_carboxypeptidase |
| C7 | 3,247114E000 | 0,000000E+001 | 4,500000E-005 | complement_component_7 |
| PINK1 | 3,238510E000 | 0,000000E+001 | 4,244000E-003 | PTEN_induced_putative_kinase_1 |
| HAMP | 3,210482E000 | 1,000000E-006 | 1,718600E-002 | hepcidin_antimicrobial_peptide |
| CXCL10 | 3,208100E000 | 0,000000E+001 | 2,200000E-005 | chemokine_(C-X-C_motif)_ligand_10 |
| SDC1 | 3,203234E000 | 0,000000E+001 | 2,954000E-003 | syndecan_1 |
| SSBP2 | 3,195249E000 | 0,000000E+001 | 8,774000E-003 | single-stranded_DNA_binding_protein_2 |
| ASAH1 | 3,170650E000 | 0,000000E+001 | 1,067000E-003 | N-acylsphingosine_amidohydrolase_(acid_ceramidase)_1 |
| CD72 | 3,162729E000 | 2,000000E-006 | 3,911800E-002 | CD72_antigen |
| UBE2J1 | 3,152830E000 | 1,000000E-006 | 2,357000E-002 | ubiquitin-conjugating_enzyme_E2_J1_(UBC6_homolog_yeast) |
| DIO3 | 3,141390E000 | 0,000000E+001 | 4,080000E-004 | deiodinase_iodothyronine_type_III |
| GLT8D2 | 3,136112E000 | 0,000000E+001 | 1,800000E-005 | glycosyltransferase_8_domain_containing_2 |
| IL16 | 3,125322E000 | 1,000000E-006 | 2,091100E-002 | interleukin_16_(lymphocyte_chemoattractant_factor) |
| LOH11CR2A | 3,119062E000 | 0,000000E+001 | 5,580000E-004 | loss_of_heterozygosity_11_chromosomal_region_2_gene_A |
| SLAMF1 | 3,102489E000 | 0,000000E+001 | 1,016600E-002 | signaling_lymphocytic_activation_molecule_family_member_1 |
| YIPF2 | 3,075723E000 | 0,000000E+001 | 7,949000E-003 | Yip1_domain_family_member_2 |
| PRKD2 | 3,054450E000 | 0,000000E+001 | 1,030000E-004 | protein_kinase_D2 |
| FOLR1 | 3,046637E000 | 0,000000E+001 | 5,000000E-006 | folate_receptor_1_(adult) |
| CD46 | 3,042608E000 | 0,000000E+001 | 5,191000E-003 | membrane_cofactor_protein_(CD46_trophoblast-lymphocyte_cross-reactive_antigen) |
| RPS21 | 3,032577E000 | 0,000000E+001 | 2,615000E-003 | ribosomal_protein_S21 |
| SEMA4D | 3,030941E000 | 0,000000E+001 | 2,030000E-004 | sema_domain_immunoglobulin_domain_transmembrane_domain_and_short_cytoplasmic_domain |
| UNC5C | 3,024006E000 | 0,000000E+001 | 2,000000E-006 | unc-5_homolog_C_(C,_elegans) |
| PRKCB1 | 3,018921E000 | 0,000000E+001 | 2,472000E-003 | protein_kinase_C_beta_1 |
| PTDSR | 3,018228E000 | 1,000000E-006 | 1,404600E-002 | phosphatidylserine_receptor |
| TCF7 | 3,009164E000 | 2,000000E-006 | 4,020800E-002 | transcription_factor_7_(T-cell_specific_HMG-box) |
| LEF1 | 3,005649E000 | 0,000000E+001 | 1,391000E-003 | lymphoid_enhancer-binding_factor_1 |
| PLCG2 | 2,999957E000 | 0,000000E+001 | 2,440000E-004 | phospholipase_C_gamma_2_(phosphatidylinositol-specific) |
| ARL1 | 2,996869E000 | 0,000000E+001 | 1,348000E-003 | ADP-ribosylation_factor-like_1 |
| METTL7A | 2,987278E000 | 0,000000E+001 | 3,864000E-003 | DKFZP586A0522_protein |
| TNFRSF1B | 2,984302E000 | 0,000000E+001 | 3,410000E-004 | tumor_necrosis_factor_receptor_superfamily_member_1B |
| JAK2 | 2,968720E000 | 0,000000E+001 | 5,800000E-005 | Janus_kinase_2_(a_protein_tyrosine_kinase) |
| FBLN5 | 2,937387E000 | 0,000000E+001 | 5,560000E-004 | fibulin_5 |
| CA12 | 2,937105E000 | 0,000000E+001 | 5,839000E-003 | carbonic_anhydrase_XII |
| OPTN | 2,932602E000 | 0,000000E+001 | 6,136000E-003 | optineurin |
| RALA | 2,919641E000 | 1,000000E-006 | 1,306200E-002 | v-ral_simian_leukemia_viral_oncogene_homolog_A_(ras_related) |
| USP14 | 2,894189E000 | 0,000000E+001 | 6,177000E-003 | ubiquitin_specific_protease_14_(tRNA-guanine_transglycosylase) |
| SOCS1 | 2,892461E000 | 2,000000E-006 | 3,410800E-002 | suppressor_of_cytokine_signaling_1 |
| SLC2A3 | 2,854307E000 | 0,000000E+001 | 3,840000E-004 | solute_carrier_family_2_(facilitated_glucose_transporter)_member_3 |
| CD44 | 2,854213E000 | 1,000000E-006 | 1,997400E-002 | CD44_antigen_(homing_function_and_Indian_blood_group_system) |
| RBP4 | 2,846795E000 | 0,000000E+001 | 5,990000E-004 | retinol_binding_protein_4_plasma |
| ECOP | 2,846376E000 | 0,000000E+001 | 2,470000E-004 | EGFR-coamplified_and_overexpressed_protein |
| PMS2L1 | 2,799267E000 | 0,000000E+001 | 4,410000E-004 | postmeiotic_segregation_increased_2-like_1 |
| SIVA1 | 2,795454E000 | 0,000000E+001 | 1,490000E-004 | CD27-binding_(Siva)_protein |
| RAP2C | 2,795145E000 | 0,000000E+001 | 2,790000E-004 | RAP2C_member_of_RAS_oncogene_family |
| PCDH9 | 2,785677E000 | 0,000000E+001 | 9,610000E-004 | protocadherin_9 |
| USP15 | 2,780556E000 | 0,000000E+001 | 3,000000E-006 | ubiquitin_specific_protease_15 |
| PRKX | 2,763227E000 | 0,000000E+001 | 2,420000E-004 | protein_kinase_X-linked |
| GTF2H1 | 2,755327E000 | 0,000000E+001 | 5,140000E-004 | general_transcription_factor_IIH_polypeptide_1_62kDa |
| PRKCB1 | 2,755008E000 | 1,000000E-006 | 1,160600E-002 | Protein_kinase_C_beta_1 |
| HIST1H4B | 2,753683E000 | 1,000000E-006 | 2,012800E-002 | histone_1_H4b |
| HIP1R | 2,725477E000 | 0,000000E+001 | 2,486000E-003 | huntingtin_interacting_protein-1-related |
| BRF2 | 2,692167E000 | 0,000000E+001 | 1,032000E-003 | BRF2_subunit_of_RNA_polymerase_III_transcription_initiation_factor_BRF1-like |
| STC1 | 2,687788E000 | 2,000000E-006 | 4,648600E-002 | stanniocalcin_1 |
| UBE2D4 | 2,679962E000 | 0,000000E+001 | 2,986000E-003 | ubiquitin-conjugating_enzyme_E2D_4_(putative) |
| GBP2 | 2,675133E000 | 0,000000E+001 | 6,800000E-005 | guanylate_binding_protein_2_interferon-inducible |
| MGP | 2,672320E000 | 0,000000E+001 | 7,400000E-005 | matrix_Gla_protein |
| PTTG1 | 2,652748E000 | 2,000000E-006 | 5,013800E-002 | pituitary_tumor-transforming_1 |
| HOXD9 | 2,648367E000 | 0,000000E+001 | 3,617000E-003 | homeo_box_D9 |
| EFEMP1 | 2,644457E000 | 0,000000E+001 | 2,420000E-004 | EGF-containing_fibulin-like_extracellular_matrix_protein_1 |
| IGFBP5 | 2,643939E000 | 0,000000E+001 | 3,770000E-004 | insulin-like_growth_factor_binding_protein_5 |
| ZFPL1 | 2,642131E000 | 0,000000E+001 | 1,949000E-003 | zinc_finger_protein-like_1 |
| DDIT4 | 2,633732E000 | 0,000000E+001 | 4,900000E-005 | DNA-damage-inducible_transcript_4 |
| TNIP2 | 2,628337E000 | 0,000000E+001 | 1,560000E-004 | TNFAIP3_interacting_protein_2 |
| UBE2J1 | 2,620774E000 | 0,000000E+001 | 1,077800E-002 | ubiquitin-conjugating_enzyme_E2_J1_(UBC6_homolog_yeast) |
| IL2RG | 2,610351E000 | 0,000000E+001 | 2,780000E-004 | interleukin_2_receptor_gamma_(severe_combined_immunodeficiency) |
| SLC10A3 | 2,605966E000 | 0,000000E+001 | 4,000000E-006 | solute_carrier_family_10_(sodium/bile_acid_cotransporter_family)_member_3 |
| RNF34 | 2,600061E000 | 1,000000E-006 | 1,173300E-002 | ring_finger_protein_34 |
| CLU | 2,586751E000 | 0,000000E+001 | 1,878000E-003 | clusterin |
| MTF2 | 2,582525E000 | 0,000000E+001 | 0,000000E+001 | metal_response_element_binding_transcription_factor_2 |
| DDX39 | 2,570805E000 | 0,000000E+001 | 1,820000E-003 | DEAD_(Asp-Glu-Ala-Asp)_box_polypeptide_39 |
| TNFAIP3 | 2,566616E000 | 0,000000E+001 | 0,000000E+001 | tumor_necrosis_factor_alpha-induced_protein_3 |
| PCDH17 | 2,553075E000 | 0,000000E+001 | 8,090000E-004 | protocadherin_17 |
| LY75 | 2,538269E000 | 0,000000E+001 | 3,900000E-005 | lymphocyte_antigen_75 |
| KIAA0746 | 2,523162E000 | 1,000000E-006 | 1,232100E-002 | KIAA0746_protein |
| STK10 | 2,512434E000 | 0,000000E+001 | 9,700000E-005 | serine/threonine_kinase_10 |

**higher variances in OA**

| **GeneSymbol** | **variance Fold** | ***P* Brown-Forsythe** | ***P* Bonferroni** | **Full Annotation** |
| --- | --- | --- | --- | --- |
| NPAT | 2,523919E-000 | 2,000000E-006 | 3,497500E-002 | nuclear_protein_ataxia-telangiectasia_locus |
| DUSP5 | 2,530517E-000 | 0,000000E+001 | 0,000000E+001 | dual_specificity_phosphatase_5 |
| ATXN7 | 2,539432E-000 | 0,000000E+001 | 9,000000E-006 | ataxin_7 |
| SETX | 2,541285E-000 | 0,000000E+001 | 2,140000E-004 | amyotrophic_lateral_sclerosis_4 |
| BIN1 | 2,567957E-000 | 1,000000E-006 | 1,751900E-002 | bridging_integrator_1 |
| SUSD4 | 2,594060E-000 | 0,000000E+001 | 1,645000E-003 | sushi_domain_containing_4 |
| NLRP2 | 2,620927E-000 | 0,000000E+001 | 1,600000E-005 | NACHT_leucine_rich_repeat_and_PYD_containing_2 |
| RAB2 | 2,625602E-000 | 0,000000E+001 | 4,584000E-003 | RAB2_member_RAS_oncogene_family |
| ATM | 2,627547E-000 | 0,000000E+001 | 8,341000E-003 | ataxia_telangiectasia_mutated_(includes_complementation_groups_A_C_and_D) |
| ODF2 | 2,628446E-000 | 0,000000E+001 | 5,280000E-004 | outer_dense_fiber_of_sperm_tails_2 |
| TAOK1 | 2,633359E-000 | 1,000000E-006 | 1,207100E-002 | TAO_kinase_1 |
| NENF | 2,641072E-000 | 0,000000E+001 | 5,000000E-006 | neuron_derived_neurotrophic_factor |
| SDHC | 2,643902E-000 | 0,000000E+001 | 3,800000E-005 | succinate_dehydrogenase_complex_subunit_C_integral_membrane_protein_15kDa |
| HIC2 | 2,650803E-000 | 1,000000E-006 | 2,515500E-002 | hypermethylated_in_cancer_2 |
| MRPL11 | 2,657887E-000 | 0,000000E+001 | 8,473000E-003 | mitochondrial_ribosomal_protein_L11 |
| IGFBP5 | 2,667414E-000 | 0,000000E+001 | 1,800000E-005 | insulin-like_growth_factor_binding_protein_5 |
| RNF5 | 2,679591E-000 | 0,000000E+001 | 6,100000E-003 | ring_finger_protein_5 |
| SCARA3 | 2,680818E-000 | 0,000000E+001 | 1,510000E-004 | scavenger_receptor_class_A_member_3 |
| ZZEF1 | 2,685064E-000 | 1,000000E-006 | 3,122400E-002 | zinc_finger_ZZ-type_with_EF-hand_domain_1 |
| CUGBP2 | 2,695814E-000 | 0,000000E+001 | 2,750000E-004 | CUG_triplet_repeat_RNA_binding_protein_2 |
| FTO | 2,699843E-000 | 0,000000E+001 | 1,235000E-003 | fatso |
| TM2D1 | 2,703525E-000 | 2,000000E-006 | 3,423700E-002 | TM2_domain_containing_1 |
| RNASE2 | 2,718444E-000 | 2,000000E-006 | 3,872900E-002 | ribonuclease_RNase_A_family_2_(liver_eosinophil-derived_neurotoxin) |
| PNPLA4 | 2,724237E-000 | 0,000000E+001 | 2,030000E-004 | patatin-like_phospholipase_domain_containing_4 |
| SLC12A8 | 2,741772E-000 | 0,000000E+001 | 4,590000E-004 | solute_carrier_family_12_(potassium/chloride_transporters)_member_8 |
| SERPINF1 | 2,743768E-000 | 0,000000E+001 | 8,326000E-003 | polymerase_(RNA)_II_(DNA_directed)_polypeptide_I_14,5kDa |
| MEOX1 | 2,746781E-000 | 0,000000E+001 | 1,056800E-002 | mesenchyme_homeo_box_1 |
| SLC38A2 | 2,760342E-000 | 1,000000E-006 | 3,330700E-002 | solute_carrier_family_38_member_2 |
| CHRNA3 | 2,788223E-000 | 1,000000E-006 | 2,058200E-002 | cholinergic_receptor_nicotinic_alpha_polypeptide_3 |
| ANKRD12 | 2,799445E-000 | 0,000000E+001 | 2,480000E-004 | ankyrin_repeat_domain_12 |
| CDC14A | 2,812867E-000 | 0,000000E+001 | 1,819000E-003 | CDC14_cell_division_cycle_14_homolog_A_(S,_cerevisiae) |
| PMVK | 2,833975E-000 | 0,000000E+001 | 8,850000E-004 | phosphomevalonate_kinase |
| GNPDA1 | 2,841633E-000 | 1,000000E-006 | 1,326000E-002 | glucosamine-6-phosphate_deaminase_1 |
| KIAA1509 | 2,850211E-000 | 0,000000E+001 | 9,841000E-003 | KIAA1509 |
| CKS1B | 2,875143E-000 | 0,000000E+001 | 1,091000E-003 | CDC28_protein_kinase_regulatory_subunit_1B |
| LSM3 | 2,887749E-000 | 0,000000E+001 | 8,700000E-004 | LSM3_homolog_U6_small_nuclear_RNA_associated_(S,_cerevisiae) |
| RAI14 | 2,922097E-000 | 1,000000E-006 | 2,433600E-002 | retinoic_acid_induced_14 |
| SLC5A3 | 2,935504E-000 | 2,000000E-006 | 4,999100E-002 | solute_carrier_family_5_(inositol_transporters)_member_3 |
| IL15 | 2,939665E-000 | 0,000000E+001 | 3,100000E-005 | interleukin_15 |
| SEC13 | 2,943101E-000 | 0,000000E+001 | 1,078400E-002 | SEC13-like_1_(S,_cerevisiae) |
| ANGPT2 | 2,960996E-000 | 0,000000E+001 | 2,521000E-003 | angiopoietin_2 |
| ITGAX | 2,967052E-000 | 0,000000E+001 | 9,110000E-004 | integrin_alpha_X_(antigen_CD11C_(p150)_alpha_polypeptide) |
| SMARCA5 | 3,009016E-000 | 0,000000E+001 | 2,082000E-003 | SWI/SNF_related_matrix_associated_actin_dependent_regulator_of_chromatin_  subfamily_a_member_5 |
| COPZ2 | 3,024685E-000 | 0,000000E+001 | 0,000000E+001 | coatomer_protein_complex_subunit_zeta_2 |
| ELF4 | 3,026837E-000 | 2,000000E-006 | 4,276300E-002 | E74-like_factor_4_(ets_domain_transcription_factor) |
| PCBP3 | 3,041368E-000 | 0,000000E+001 | 4,400000E-003 | poly(rC)_binding_protein_3 |
| HNMT | 3,091152E-000 | 1,000000E-006 | 3,100100E-002 | histamine_N-methyltransferase |
| CRYL1 | 3,112624E-000 | 0,000000E+001 | 2,106000E-003 | crystallin_lambda_1 |
| NBN | 3,122666E-000 | 0,000000E+001 | 8,474000E-003 | nibrin |
| MYCBP2 | 3,123510E-000 | 1,000000E-006 | 2,748200E-002 | MYC_binding_protein_2 |
| PCDHB11 | 3,134466E-000 | 2,000000E-006 | 4,050200E-002 | protocadherin_beta_11 |
| PRDX2 | 3,135311E-000 | 0,000000E+001 | 3,397000E-003 | peroxiredoxin_2 |
| TRIM22 | 3,178174E-000 | 0,000000E+001 | 4,390000E-004 | tripartite_motif-containing_22 |
| NCOR1 | 3,219254E-000 | 1,000000E-006 | 2,368600E-002 | nuclear_receptor_co-repressor_1 |
| MSRB2 | 3,221697E-000 | 0,000000E+001 | 6,450000E-004 | methionine_sulfoxide_reductase_B2 |
| ATP2A3 | 3,237004E-000 | 0,000000E+001 | 1,600000E-005 | ATPase_Ca++_transporting_ubiquitous |
| POLR2E | 3,261477E-000 | 1,000000E-006 | 1,129500E-002 | polymerase_(RNA)_II_(DNA_directed)_polypeptide_E_25kDa |
| NDUFA8 | 3,267943E-000 | 0,000000E+001 | 6,109000E-003 | NADH_dehydrogenase_(ubiquinone)_1_alpha_subcomplex_8_19kDa |
| MDN1 | 3,271166E-000 | 0,000000E+001 | 8,236000E-003 | MDN1_midasin_homolog_(yeast) |
| CEP350 | 3,276622E-000 | 0,000000E+001 | 2,100000E-005 | centrosome-associated_protein_350 |
| MAPK11 | 3,308783E-000 | 0,000000E+001 | 3,550000E-004 | mitogen-activated_protein_kinase_11 |
| NDUFC1 | 3,326205E-000 | 1,000000E-006 | 1,854800E-002 | NADH_dehydrogenase_(ubiquinone)_1_subcomplex_unknown_1_6kDa |
| WIF1 | 3,335629E-000 | 0,000000E+001 | 4,000000E-006 | WNT_inhibitory_factor_1 |
| OPRS1 | 3,348552E-000 | 1,000000E-006 | 2,415000E-002 | opioid_receptor_sigma_1 |
| ATP5G3 | 3,372982E-000 | 2,000000E-006 | 3,386300E-002 | ATP_synthase_H+_transporting_mitochondrial_F0_complex_subunit_c_(subunit_9)_isoform_3 |
| CUGBP1 | 3,383949E-000 | 1,000000E-006 | 1,736900E-002 | CUG_triplet_repeat_RNA_binding_protein_1 |
| JARID2 | 3,439378E-000 | 0,000000E+001 | 1,739000E-003 | Jumonji_AT_rich_interactive_domain_2 |
| PUM2 | 3,448585E-000 | 0,000000E+001 | 3,192000E-003 | pumilio_homolog_2_(Drosophila) |
| GCHFR | 3,454294E-000 | 0,000000E+001 | 3,000000E-006 | GTP_cyclohydrolase_I_feedback_regulator |
| VTI1B | 3,465492E-000 | 1,000000E-006 | 2,372800E-002 | vesicle_transport_through_interaction_with_t-SNAREs_homolog_1B_(yeast) |
| RASGRP3 | 3,479409E-000 | 0,000000E+001 | 4,500000E-003 | RAS_guanyl_releasing_protein_3_(calcium_and_DAG-regulated) |
| ATP13A3 | 3,487315E-000 | 0,000000E+001 | 3,700000E-004 | ATPase_type_13A3 |
| TMEM30A | 3,489602E-000 | 0,000000E+001 | 3,369000E-003 | transmembrane_protein_30A |
| DNAJC3 | 3,557245E-000 | 0,000000E+001 | 3,099000E-003 | DnaJ_(Hsp40)_homolog_subfamily_C_member_3 |
| IER5 | 3,605483E-000 | 0,000000E+001 | 1,350000E-004 | immediate_early_response_5 |
| BTN2A1 | 3,641211E-000 | 0,000000E+001 | 4,790000E-004 | butyrophilin_subfamily_2_member_A1 |
| APLP2 | 3,643887E-000 | 0,000000E+001 | 1,020000E-004 | amyloid_beta_(A4)_precursor-like_protein_2 |
| SAP18 | 3,664556E-000 | 0,000000E+001 | 9,719000E-003 | sin3-associated_polypeptide_18kDa |
| LILRA2 | 3,678766E-000 | 0,000000E+001 | 5,000000E-005 | leukocyte_immunoglobulin-like_receptor_subfamily_A_(with_TM_domain)_member_2 |
| NELL1 | 3,712027E-000 | 0,000000E+001 | 3,100000E-005 | NEL-like_1_(chicken) |
| ADORA1 | 3,716979E-000 | 1,000000E-006 | 2,593300E-002 | adenosine_A1_receptor |
| ST6GALNAC5 | 3,741742E-000 | 2,000000E-006 | 3,496800E-002 | ST6_(alpha-N-acetyl-neuraminyl-23-beta-galactosyl-13)-N-acetylgalactosaminide_  alpha-26-sialyltransfe |
| DPM3 | 3,745545E-000 | 0,000000E+001 | 1,751000E-003 | dolichyl-phosphate_mannosyltransferase_polypeptide_3 |
| APBA3 | 3,791596E-000 | 1,000000E-006 | 1,501200E-002 | amyloid_beta_(A4)_precursor_protein-binding_family_A_member_3_(X11-like_2) |
| NOTCH1 | 3,819404E-000 | 0,000000E+001 | 2,300000E-005 | Notch_homolog_1_translocation-associated_(Drosophila) |
| MACF1 | 3,823363E-000 | 0,000000E+001 | 8,600000E-004 | microtubule-actin_crosslinking_factor_1 |
| THRA | 3,856725E-000 | 0,000000E+001 | 1,087000E-003 | thyroid_hormone_receptor_alpha_(erythroblastic_leukemia_viral_oncogene_homolog_avian) |
| FAS | 3,858145E-000 | 0,000000E+001 | 2,000000E-005 | Fas_(TNF_receptor_superfamily_member_6) |
| PLXNA1 | 3,876532E-000 | 0,000000E+001 | 2,967000E-003 | plexin_A1 |
| TP53AP1 | 3,900504E-000 | 1,000000E-006 | 2,565500E-002 | TP53_activated_protein_1 |
| ANKH | 3,908728E-000 | 0,000000E+001 | 3,066000E-003 | ankylosis_progressive_homolog_(mouse) |
| ATP2A2 | 3,952549E-000 | 0,000000E+001 | 8,709000E-003 | ATPase_Ca++_transporting_cardiac_muscle_slow_twitch_2 |
| APLP2 | 3,989968E-000 | 1,000000E-006 | 2,409300E-002 | amyloid_beta_(A4)_precursor-like_protein_2 |
| NDUFA13 | 3,992026E-000 | 0,000000E+001 | 6,080000E-004 | NADH_dehydrogenase_(ubiquinone)_1_alpha_subcomplex_13 |
| MACF1 | 4,023548E-000 | 1,000000E-006 | 2,547100E-002 | microtubule-actin_crosslinking_factor_1 |
| NDUFA6 | 4,065492E-000 | 0,000000E+001 | 1,513000E-003 | NADH_dehydrogenase_(ubiquinone)_1_alpha_subcomplex_6_14kDa |
| CAMTA1 | 4,087778E-000 | 0,000000E+001 | 3,900000E-005 | calmodulin_binding_transcription_activator_1 |
| NDUFB6 | 4,189034E-000 | 0,000000E+001 | 3,570000E-004 | NADH_dehydrogenase_(ubiquinone)_1_beta_subcomplex_6_17kDa |
| CHMP2A | 4,255350E-000 | 1,000000E-006 | 2,175700E-002 | chromatin_modifying_protein_2A |
| INTS6 | 4,302092E-000 | 2,000000E-006 | 3,800400E-002 | DEAD/H_(Asp-Glu-Ala-Asp/His)_box_polypeptide_26 |
| CHD8 | 4,308801E-000 | 0,000000E+001 | 2,054000E-003 | chromodomain_helicase_DNA_binding_protein_8 |
| ZFAND5 | 4,358951E-000 | 0,000000E+001 | 2,150000E-004 | zinc_finger_A20_domain_containing_2 |
| GPR153 | 4,455412E-000 | 1,000000E-006 | 1,406700E-002 | G_protein-coupled_receptor_153 |
| DDEF1 | 4,473048E-000 | 0,000000E+001 | 1,441000E-003 | development_and_differentiation_enhancing_factor_1 |
| HSPE1 | 4,477292E-000 | 0,000000E+001 | 1,069000E-003 | heat_shock_10kDa_protein_1_(chaperonin_10) |
| TMEM4 | 4,676431E-000 | 0,000000E+001 | 2,425000E-003 | transmembrane_protein_4 |
| VPS13D | 4,681422E-000 | 0,000000E+001 | 3,710000E-003 | vacuolar_protein_sorting_13D_(yeast) |
| CREB3 | 4,810744E-000 | 0,000000E+001 | 6,386000E-003 | cAMP_responsive_element_binding_protein_3 |
| SBNO1 | 4,872016E-000 | 1,000000E-006 | 2,275500E-002 | sno_strawberry_notch_homolog_1_(Drosophila) |
| ZNF226 | 4,882666E-000 | 2,000000E-006 | 4,853800E-002 | zinc_finger_protein_226 |
| UBN1 | 4,893113E-000 | 1,000000E-006 | 2,844300E-002 | ubinuclein_1 |
| FAM82C | 4,977147E-000 | 0,000000E+001 | 1,000000E-006 | family_with_sequence_similarity_82_member_C |
| MS4A2 | 4,979918E-000 | 0,000000E+001 | 1,392000E-003 | membrane-spanning_4-domains_subfamily_A_member_2 |
| SDS | 5,025659E-000 | 1,000000E-006 | 1,364100E-002 | serine_dehydratase |
| SPTAN1 | 5,212917E-000 | 1,000000E-006 | 2,995300E-002 | spectrin_alpha_non-erythrocytic_1_(alpha-fodrin) |
| HPD | 5,256057E-000 | 0,000000E+001 | 2,127000E-003 | 4-hydroxyphenylpyruvate_dioxygenase |
| FRYL | 5,393599E-000 | 0,000000E+001 | 2,200000E-005 | KIAA0826 |
| RHD | 5,395827E-000 | 0,000000E+001 | 5,210000E-004 | Rhesus_blood_group_D_antigen |
| GTF2A2 | 5,419738E-000 | 0,000000E+001 | 9,000000E-006 | general_transcription_factor_IIA_2_12kDa |
| NDUFB4 | 5,449374E-000 | 0,000000E+001 | 8,440000E-004 | NADH_dehydrogenase_(ubiquinone)_1_beta_subcomplex_4_15kDa |
| DCTN3 | 5,516932E-000 | 0,000000E+001 | 3,655000E-003 | dynactin_3_(p22) |
| PUM2 | 5,834646E-000 | 0,000000E+001 | 5,512000E-003 | pumilio_homolog_2_(Drosophila) |
| BRP44L | 5,898513E-000 | 1,000000E-006 | 3,050800E-002 | brain_protein_44-like |
| VRK3 | 5,920353E-000 | 0,000000E+001 | 1,150000E-004 | vaccinia_related_kinase_3 |
| EPS8L3 | 5,939377E-000 | 0,000000E+001 | 3,610000E-004 | EPS8-like_3 |
| SGCA | 6,012134E-000 | 0,000000E+001 | 0,000000E+001 | sarcoglycan_alpha_(50kDa_dystrophin-associated_glycoprotein) |
| GFRA2 | 6,486527E-000 | 1,000000E-006 | 3,288500E-002 | GDNF_family_receptor_alpha_2 |
| NDUFB2 | 6,523115E-000 | 2,000000E-006 | 4,795400E-002 | NADH_dehydrogenase_(ubiquinone)_1_beta_subcomplex_2_8kDa |
| WWC3 | 6,560006E-000 | 0,000000E+001 | 1,030000E-004 | KIAA1280_protein |
| ST3GAL2 | 6,592453E-000 | 0,000000E+001 | 9,300000E-005 | ST3_beta-galactoside_alpha-23-sialyltransferase_2 |
| METTL5 | 6,594451E-000 | 1,000000E-006 | 2,259600E-002 | methyltransferase_like_5 |
| GPR153 | 6,730341E-000 | 1,000000E-006 | 3,152300E-002 | G_protein-coupled_receptor_153 |
| WDR61 | 6,929225E-000 | 0,000000E+001 | 4,520000E-003 | WD_repeat_domain_61 |
| MKI67 | 6,939629E-000 | 1,000000E-006 | 2,210600E-002 | antigen_identified_by_monoclonal_antibody_Ki-67 |
| COX5B | 7,368363E-000 | 2,000000E-006 | 3,673500E-002 | cytochrome_c_oxidase_subunit_Vb |
| KBTBD2 | 7,475550E-000 | 0,000000E+001 | 4,319000E-003 | kelch_repeat_and_BTB_(POZ)_domain_containing_2 |
| SYCP2 | 7,628805E-000 | 0,000000E+001 | 8,220000E-004 | synaptonemal_complex_protein_2 |
| CYLD | 7,832717E-000 | 1,000000E-006 | 2,500300E-002 | cylindromatosis_(turban_tumor_syndrome) |
| SNX26 | 9,047922E-000 | 0,000000E+001 | 1,013600E-002 | sorting_nexin_26 |
| EPYC | 9,210312E-000 | 0,000000E+001 | 2,500000E-005 | dermatan_sulfate_proteoglycan_3 |
| LILRA2 | 9,873937E-000 | 1,000000E-006 | 3,196700E-002 | leukocyte_immunoglobulin-like_receptor_subfamily_A_(with_TM_domain)_member_2 |
| ANGPT2 | 1,083244E+001 | 0,000000E+001 | 5,440000E-004 | angiopoietin_2 |
| ASCC3 | 1,171554E+001 | 2,000000E-006 | 5,032800E-002 | activating_signal_cointegrator_1_complex_subunit_3 |
| STMN2 | 1,189912E+001 | 0,000000E+001 | 6,600000E-005 | stathmin-like_2 |
| ULK2 | 1,246917E+001 | 2,000000E-006 | 4,296300E-002 | Unc-51-like_kinase_2_(C,_elegans) |
| CRYBB1 | 1,260065E+001 | 0,000000E+001 | 1,238000E-003 | crystallin_beta_B1 |
| CLDN15 | 1,295216E+001 | 1,000000E-006 | 2,131000E-002 | claudin_15 |
